# Cellular Processes Involved in Jurkat Cells Exposed to Nanosecond Pulsed Electric Field

**DOI:** 10.3390/ijms20235847

**Published:** 2019-11-21

**Authors:** Huijuan Li, Shibin Liu, Xue Yang, Yongqian Du, Jiezhang Luo, Jie Tan, Yulong Sun

**Affiliations:** 1 School of Electronics and Information, Northwestern Polytechnical University, Xi’an 710072, China; lihuijuan@mail.nwpu.edu.cn (H.L.); yangx@mail.nwpu.edu.cn (X.Y.); duyongqian@nwpu.edu.cn (Y.D.); 18829237964@163.com (J.L.); jietan@mail.nwpu.edu.cn (J.T.); 2 Key Laboratory for Space Biosciences & Biotechnology, School of Life Sciences, Northwestern Polytechnical University, Xi’an 710072, China

**Keywords:** nanosecond pulsed electric field (nsPEF), Jurkat, microarray, bioinformatics analysis, molecular dynamics simulation

## Abstract

Recently, nanosecond pulsed electric field (nsPEF) has been considered as a new tool for tumor therapy, but its molecular mechanism of function remains to be fully elucidated. Here, we explored the cellular processes of Jurkat cells exposed to nanosecond pulsed electric field. Differentially expressed genes (DEGs) were acquired from the GEO2R, followed by analysis with a series of bioinformatics tools. Subsequently, 3D protein models of hub genes were modeled by Modeller 9.21 and Rosetta 3.9. Then, a 100 ns molecular dynamics simulation for each hub protein was performed with GROMACS 2018.2. Finally, three kinds of nsPEF voltages (0.01, 0.05, and 0.5 mV/mm) were used to simulate the molecular dynamics of hub proteins for 100 ns. A total of 1769 DEGs and eight hub genes were obtained. Molecular dynamic analysis, including root mean square deviation (RMSD), root mean square fluctuation (RMSF), and the R*g*, demonstrated that the 3D structure of hub proteins was built, and the structural characteristics of hub proteins under different nsPEFs were acquired. In conclusion, we explored the effect of nsPEF on Jurkat cell signaling pathway from the perspective of molecular informatics, which will be helpful in understanding the complex effects of nsPEF on acute T-cell leukemia Jurkat cells.

## 1. Introduction

Leukemia is a malignant cancer of blood cells that usually starts in the bone marrow and results in excessive numbers of aberrant blood cells. Due to the lack of normal blood cells, leukemia patients often have symptoms including fever, feeling tired, bleeding and bruising, and are prone to infections. At present, the incidence of leukemia in the world is gradually increasing, which seriously affects the labor force and imposes a heavy burden on medical care in various countries [1]. In 2019, there will be 61,780 new leukemia patients in the United States. Leukemia is the most common type of cancer for teenagers before the age of 14, and approximately 92% of leukemia patients are diagnosed before the age of 20 [2]. Therefore, the treatment of leukemia is one of the key issues that needs to be solved urgently in the medical field.

As a malignant disease of the blood system, leukemia has a huge classification system. From a pathological point of view, the type of leukemia is mainly distinguished by the type of immature white blood cells in the blood. Clinically, leukemia is generally classified into acute and chronic leukemia. Among the many subtypes of leukemia, acute leukemia accounts for more than 70% of children [1,2]. Jurkat cells are a leukemia cell line isolated from the peripheral blood of a boy with acute T lymphocytic leukemia, which greatly facilitates the study of the T-cell acute lymphoblastic leukemia (T-ALL).

T-cell acute lymphoblastic leukemia (T-ALL) is a type of acute leukemia that progresses rapidly. It invades the lymphoid-cell-generating stem cells, in particular a type of white blood cell named T lymphocytes. The clinical symptoms of patients with T-ALL are variable, including the large mediastinal mass, high white blood cells count, central nervous system (CNS) invasion and organomegaly [3]. Current treatments for T-ALL patients include: (1) biotechnological treatment, (2) bone marrow transplantation, and (3) chemotherapy drugs. The method of biotechnology treatment is to change the genetic information in the patient’s body to achieve therapeutic purposes by means of “gene editing”, which is a promising approach, but the safety and stability of the method remains to be observed. Allogenic bone marrow transplantation is currently the only way to cure the disease, but treatment opportunities are extremely low and expensive. Chemotherapy, the only treatment approach currently used by the vast majority of patients, is used to reduce or even completely relieve symptoms by administering chemical small molecule drugs to the patient to kill leukemia cells. The pathogenesis of T-ALL is a complex network, which involves the regulation and mutual regulation of multiple signaling pathways. Therefore, the current research on the pathogenesis of T-ALL mainly includes the following components: PI3K-AKT-mTOR, IL7R, BRD4/MYC, NOTCH1, BCL2, Cyclin D3 (CDK4/CDK6), SINE, and rationale combinations of above approaches [3]. Overall, an in-depth study of the signaling pathway of T-ALL can help to understand its pathogenesis and develop corresponding drugs.

The application of electric field in cancer treatment, previously uncharted, has become an emerging field recently [3]. The use of the electric field as a physical stimulus to treat tumor cells not only provides experimental data for the electrochemical treatment of tumors but also lays the foundation for the development of more efficient anti-tumor therapies in the future. Hence, electrochemical therapy of tumors has become one of the promising concerns of current clinical anti-tumor therapy.

Regarding the influence of electric field on leukemia cells, predecessors have performed interesting progress. After being treated by electric field strengths from 500 to 800 V/cm, the morphology and volume of human leukemia cell k562 was changed significantly [4]. Leukemia cells co-treated by cationic peptide and pulsed electric field are more prone to cell membrane rupture than cells treated only by pulsed electric field, suggesting that cationic peptides may be an effective cofactor for electrochemotherapy of leukemia [5]. Moreover, electric field exposure of leukemia cell-derived RNA would be an efficient tool to load dendritic cells with tumor antigens to develop the dendritic cell-based cancer immunotherapy [6]. In addition, the electric field can be used not only for the development of platforms to sort the leukemia cells [7], but also for the display of individual organelles of leukemia cells [8].

However, most studies mainly focus on investigating cell behavior changes, such as proliferation, migration, and electrotaxis, whereas there are rare reports on the signaling pathway of leukemia cells under electric field. Moreover, according to our best knowledge, no research has yet been reported focusing on constructing the qualified 3D protein models of electric field-sensitive proteins, which seriously delays the development of pharmacological therapeutic drugs against these proteins.

**Figure 1:**
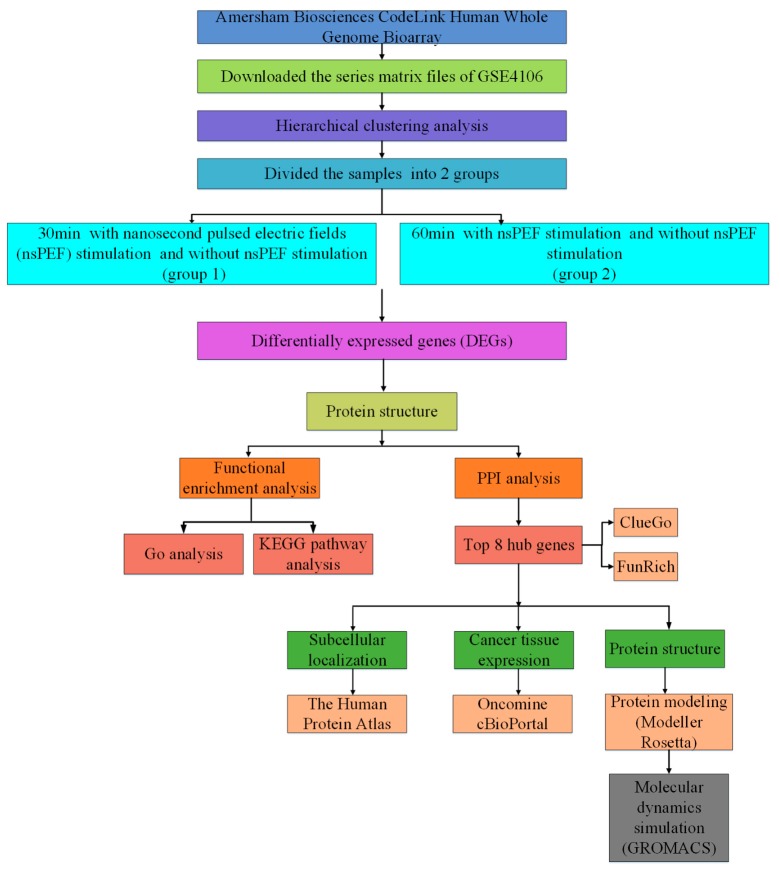
Schematic overview of the workflow.

In the present study, the nsPEF-sensitive genes of leukemia cells were acquired with integrated bioinformatic analysis from Jurkat microarray data. Next, hub proteins were subjected to molecular dynamics simulation (at least 100 ns) to acquire detailed structural information characteristics and to establish their 3D protein models. The aim of this study is to: (1) acquire nsPEFs-sensitive genes and investigate their cell-level signaling pathways; (2) construct three-dimensional protein structure models of the hub genes; (3) explore the structural characteristics of hub proteins on a microscopic scale (Figure 1). In summary, we sought to identify nsPEF-sensitive genes in Jurkat cells from the perspective of molecular informatics, which provided a preliminary exploration for understanding the effect of nsPEF on Jurkat cells.

## 2. Results

### 2.1. Identification of DEGs

The workflow for this article is shown in Figure 1. The microarray dataset GSE4106 was downloaded from the Gene Expression Omnibus (GEO) database. A total of 1669 DEGs were identified in group 1, which contained 173 up-regulated and 1496 down-regulated genes. Meanwhile, group 2 consisted of 1676 DEGs, which contained 171 up-regulated and 1505 down-regulated genes.

**Figure 2:**
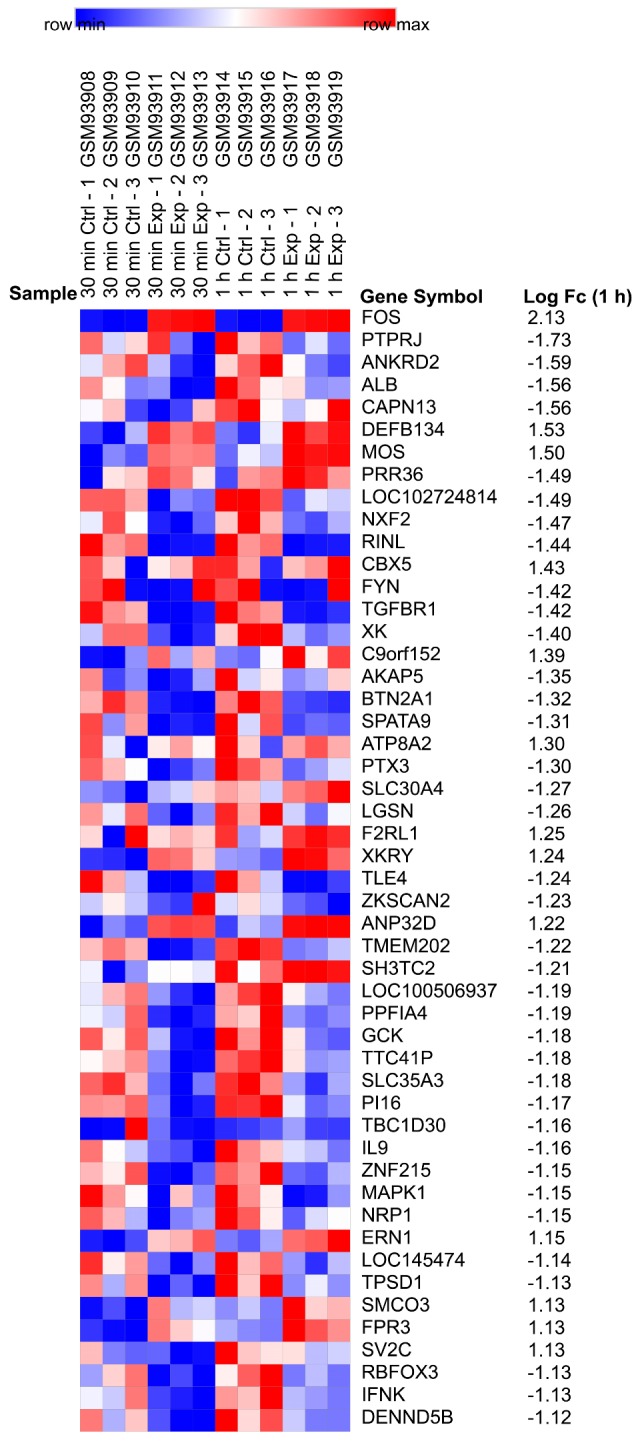
Heat map of the top 50 differentially expressed genes (DEGs). These genes were selected in descending order of their absolute values of LogFC of DEGs. Data from Jurkat cells treated with nanosecond pulsed electric field (nsPEF) for 60 min were shown.

Based on their absolute values of LogFC, a total of 50 genes were selected from the DEGs, followed by analyzing with the Morpheus (https://software.broadinstitute.org/morpheus/) online software to construct the heat map (Figure 2) [9]. Furthermore, the biological processes functional enrichment and pathway enrichment analyses for DEGs were investigated using FunRich (http://www.funrich.org) software [10]. As shown in Figure S1a,b, the interactions nodes less than 10 were removed from group 1 and 2.

### 2.2. Functional and Pathway Enrichment Analysis of Identified Modules Associated with DEGs

**Figure 3:**
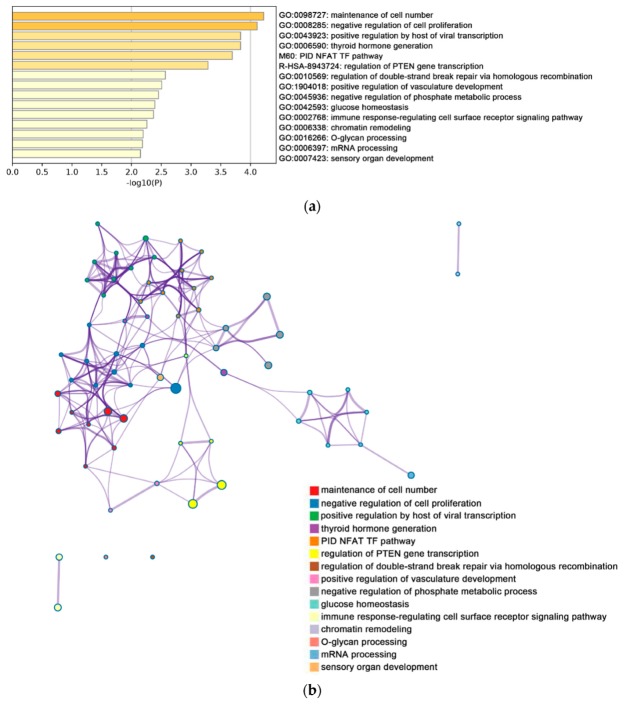

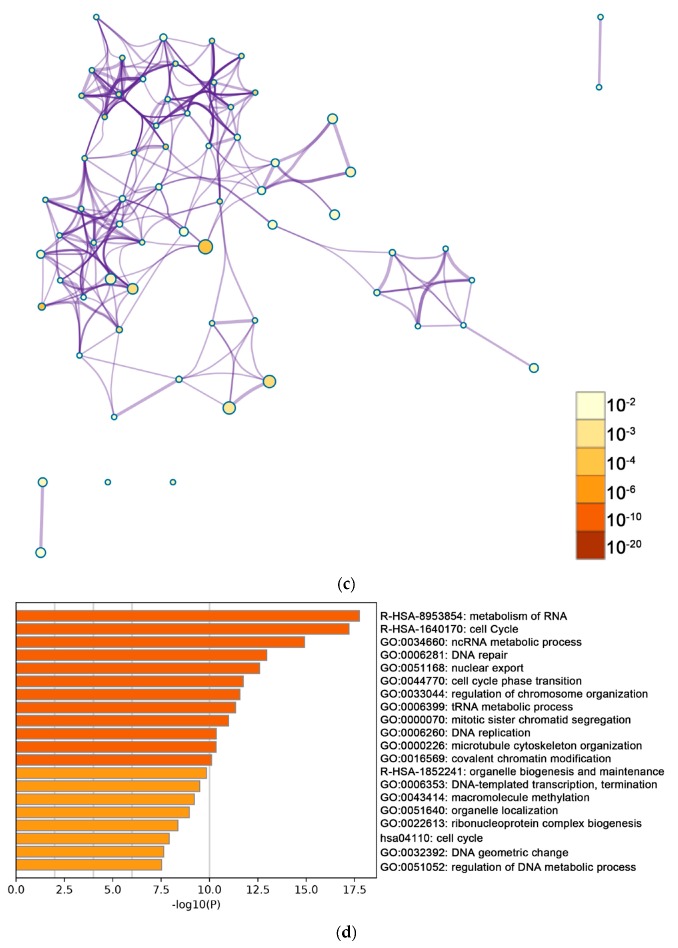

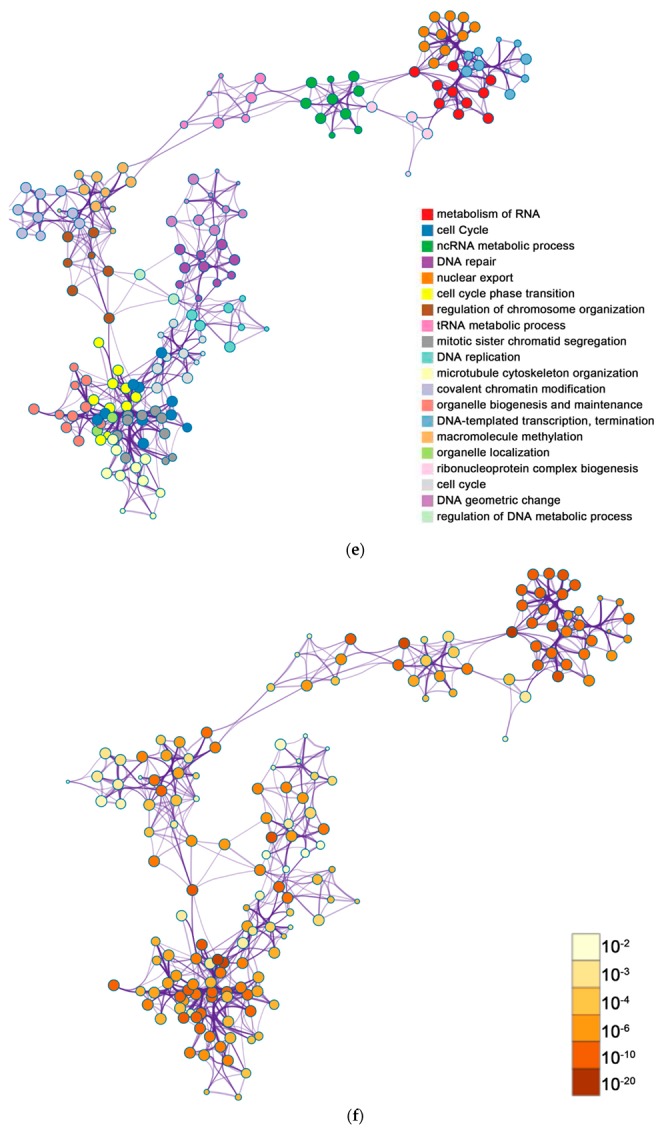

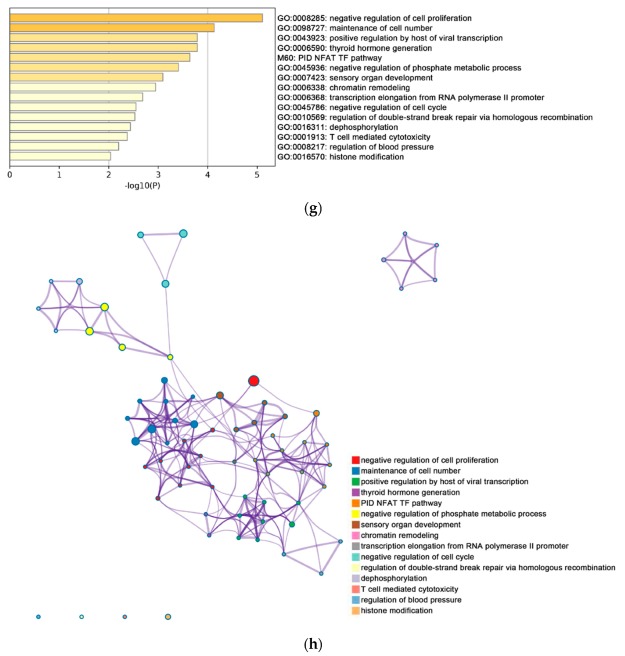

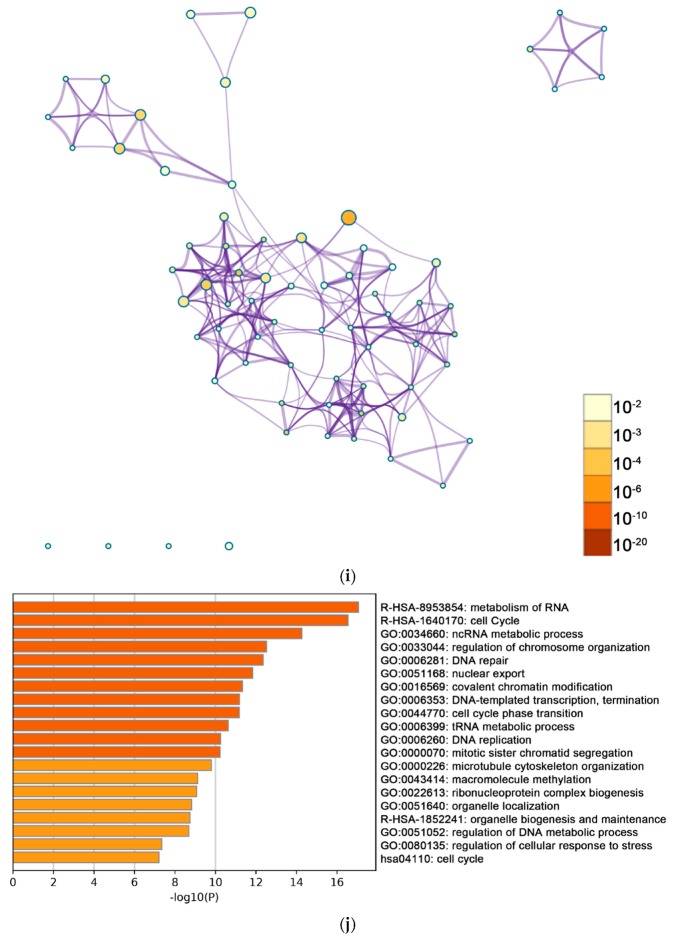

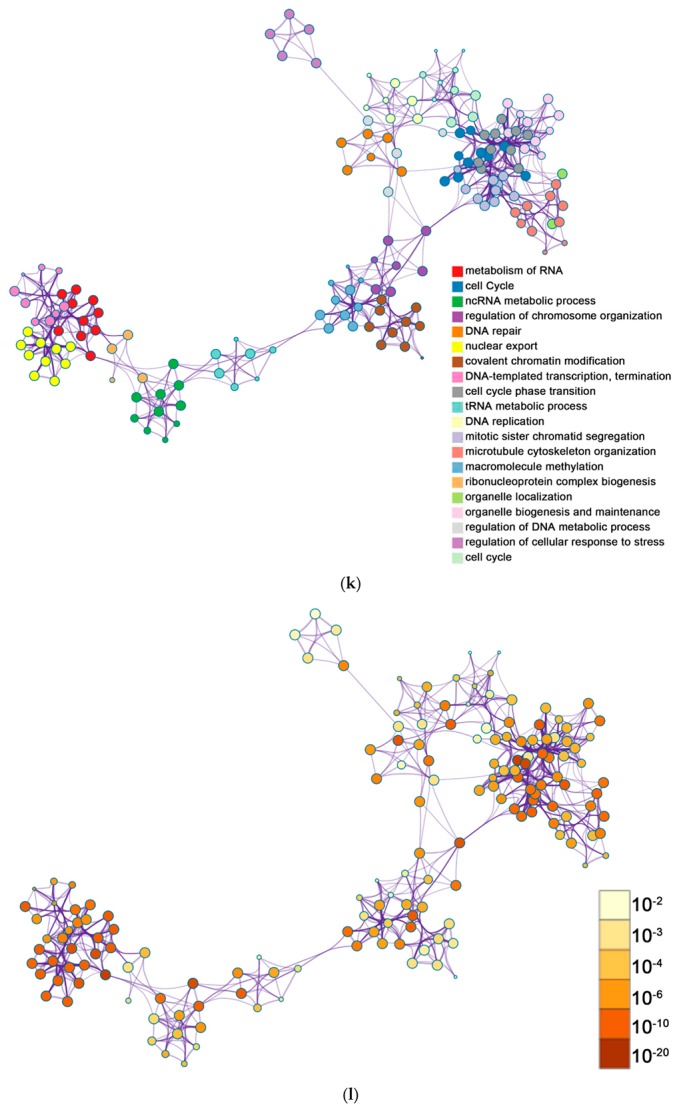
The enrichment analysis was performed by Metascape. (**a**) Bar graph demonstrating biological processes enrichment analysis of up-regulated genes of group 1. For down-regulated genes of group 1, Metascape only visualized the top 15 clusters by their colors. Enriched terms were identified according to the Kappa similarity >0.3. Each node represents an enriched term, and the nodes are colored by their cluster IDs (**b**) and *p*-values (**c**) separately. The down-regulated genes of group 1 are illustrated in the same way, as (**d**) shows the bar graph of biological processes enrichment analysis. The top 20 clusters are colored by their cluster IDs, while the *p*-values are displayed in (**e**,**f**) separately. For up-regulated genes of group 2, (**g**) shows the bar graph of biological processes enrichment analysis. The top 15 clusters are colored by their cluster IDs and *p*-values, which are displayed in (**h**,**i**), respectively. For down-regulated genes of group 2, (**j**) gives the bar graph of biological processes enrichment analysis. The top 20 clusters are colored by their cluster IDs and their *p*-values are illustrated in (**k**,**l**), respectively. All graphs were generated by Metascape (http://metascape.org/gp/index.html#/main/step1).

Function and pathway enrichment analyses were performed based on the online bioinformatics database Metascape. As shown in Figure 3, the enriched terms related to DEGs of group 1 were analyzed by Metascape.

In group 1, the enrichment pathway of up-regulated genes mainly included maintenance of cell number and negative regulation of cell proliferation (Figure 3a), while the enrichment pathway of down-regulated genes mainly included metabolism of RNA, cell cycle, and ncRNA metabolic process (Figure 3d). In addition, the enriched terms were collected and listed based on their *p*-values and cluster (Figure 3b,c,e,f). For group 2, the significantly enriched pathways of up-regulated genes included maintenance of cell number and negative regulation of cell proliferation, whereas the enrichment pathway of down-regulated genes included metabolism of RNA, cell cycle, and ncRNA metabolic process. Enrichment processes were highly connected and aggregated together by *p*-value and clusters (Figure 3g–l).

As shown in Table 1, after Jurkat cells were stimulated with nsPEF for 30 min and 60 min, most enrichment pathways of the down-regulated genes in group 1 and group 2 were identical, whereas the lots of enrichment pathways of up-regulated genes were different. The change hinted a change in the signal pathways of Jurkat cells.

**Figure 4:**
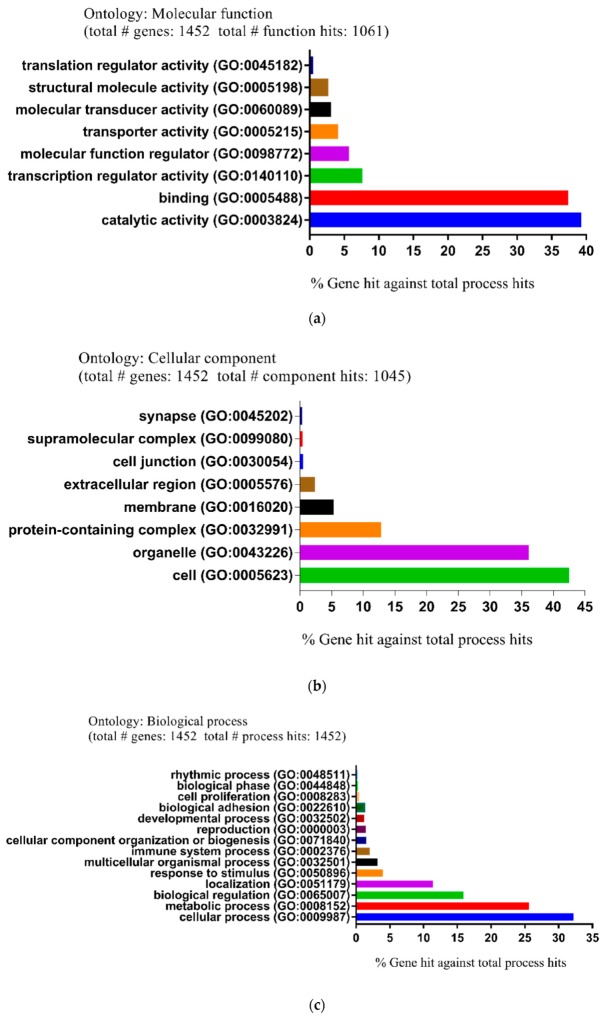

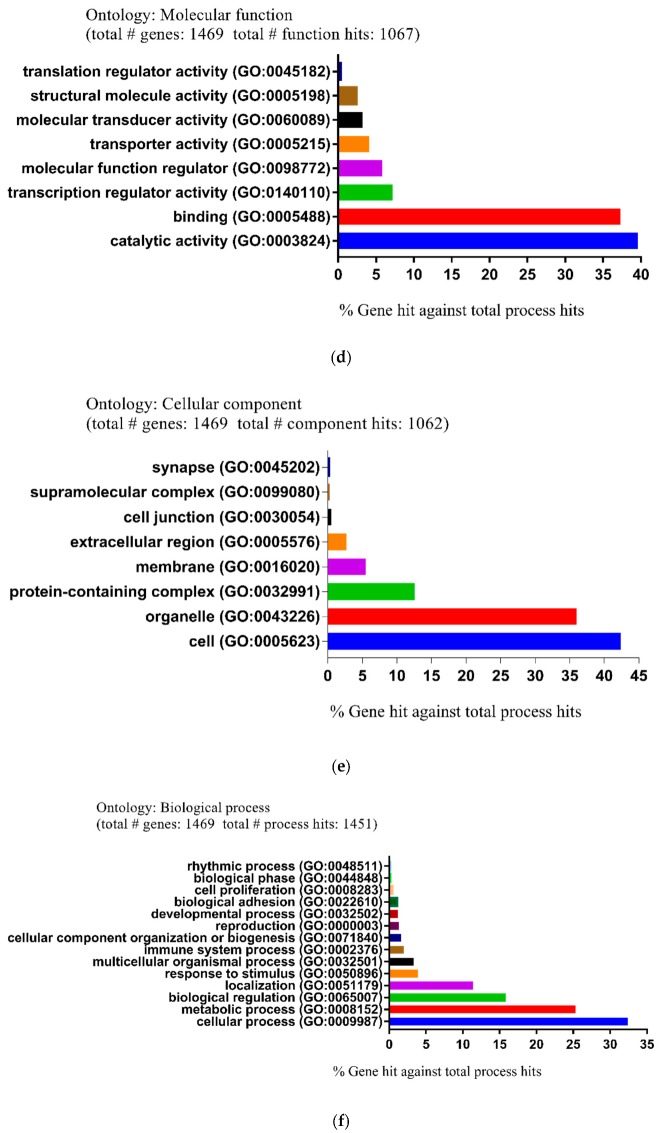
Functional and pathway enrichment analysis of identified modules associated with DEGs. The DEGs were subjected to gene ontology (GO) classification using the PANTHER GO classification system. Group 1: (**a**) Molecular function (MF). (**b**) Cellular component (CC). (**c**) Biological process (BP). Group 2: (**d**) Molecular function (MF). (**e**) Cellular component (CC). (**f**) Biological process (BP).

Moreover, the biological functions and pathways of DEGs were also investigated by using the PANTHER GO classification system, which consisted of three main gene ontology (GO) categories: Molecular function (MF), cellular component (CC), and biological process (BP). In group 1, for the MF, the main differential expression proteins were involved in catalytic activity (39.3%), binding (37.4%), and transcription regulator activity (7.6%) (Figure 4a). For the CC, the majority of proteins anticipated in processes such as cell (42.5%), organelle (36.1%), and protein-containing complex (12.8%) (Figure 4b). For the BP, proteins were associated with cellular process (32.2%), metabolic process (25.6%), and biological regulation (15.9%) (Figure 4c).

In group 2, for the MF, the main differential expression proteins anticipated in catalytic activity (39.6%), binding (37.3%), and transcription regulator activity (7.2%) (Figure 4d). For the CC, the majority of proteins were involved in cell (42.4%), organelle (36%), and protein-containing complex (12.6%) (Figure 4e). For the BP, proteins were closely linked to cellular process (32.4%), metabolic process (25.3%), and biological regulation (15.8%) (Figure 4f). Furthermore, the most significantly enriched pathways of down- and up-regulated DEGs in group 1 and 2 were analyzed by the Kyoto Encyclopedia of Genes and Genomes (KEGG) website (http://www.genome.jp/kegg/) (Table 2). 

### 2.3. Module Screening from the PPI Network

Protein–protein interactions (PPIs) between DEGs were predicted by using the online server Search Tool for the Retrieval of Interacting Genes (STRING). For each group, the top three sub-networks were selected for the subsequent pathway enrichment analysis (Figure S2).

In group 1, a total of 433 nodes and 1778 edges were identified using plugin MCODE, and the enrichment data showed that the following processes were activated: RNA splicing, protein ubiquitination, and microtubule cytoskeleton organization. Meanwhile, a total of 733 nodes and 3580 edges were found in group 2, indicating that the following pathways were stimulated: RNA splicing, nuclear division, and microtubule cytoskeleton organization.

Subsequently, with the aid of the cytoHubba plug-in, the top eight hub nodes (*SUGP1*, *DHX16*, *FUS*, *HNRNPR*, *DHX15*, *NAA38*, *SKIV2L2* and *PLRG1*) with higher degrees were obtained.

Finally, the top 100 genes were selected from the two groups based on *p*-values, followed by pathway enrichment analyses with ClueGO and CluePedia plugins. The kappa coefficient of 0.4 and *p*-value ≤ 0.05 were considered as reliable threshold values. In group 1, the results showed that 17 pathways could be divided into seven categories, which included regulation of axon regeneration, ER to Golgi transport vesicle membrane, and so on (Figure S3). For group 2, there were 13 pathways that could be classified into seven categories, such as regulation of neuron projection regeneration; histone methyltransferase activity, etc. (Figure S4).

### 2.4. Sub-Localization Expression Analysis of Hub Genes

With the help of the open-source database Human Protein Atlas (HPA) (http://www.proteinatlas.org), the experimental evidence of the sub-localization of hub genes was acquired, including *SUGP1*, *DHX16*, *FUS*, *HNRNPR*, *DHX15*, *NAA38*, *SKIV2L2*, and *PLRG1*.

The sub-localization of *SUGP1* in human cells demonstrated that SUGP1 protein existed at the nucleoplasm of A-431, U-2, and U-251 MG cells (Figure S5). As shown in Supplementary Materials (Figures S6–S12), the other hub genes distributed at different zones of the cells (Table 3).

### 2.5. Mining Genetic Alterations Connected with Hub Genes by cBioportal

To further explore the clinical significance of these hub genes (*SUGP1*, *DHX16*, *FUS*, *HNRNPR*, *DHX15*, *NAA38*, *SKIV2L2*, and *PLRG1*), we used a series of online tools to evaluate the clinical expression of these hub genes in leukemia.

As demonstrated in Figure S13a, amplification and deep deletion usually happened in *SUGP1*, *DHX16*, *FUS*, *HNPNPR*, *DHX15*, and *NAA38*. Meanwhile, the following genes mainly have deep deletion and missense mutation: *FUS*, *DHX15*, *PLRG1*, and *MTREX*. Moreover, the alterations of eight hub genes ranged from 0.5% to 1.5% in 13 leukemia tissues [11] (Figure S13b). In addition, the significance of these hub genes in tumor survival time and their distribution in leukemia tissues have also been studied (Figures S13c–e and S14–S20).

### 2.6. The Eight Hub Genes Expressed in Leukemia by Using Oncomine

Given the importance of Oncomine in the tumor field, the expression of these eight hub genes was also evaluated by using Oncomine. As demonstrated in Figure S21, the mRNA expression of *HNRNPR* in T-cell acute lymphoblastic leukemia was higher than in bone marrow. Moreover, the expression of other hub genes was also presented (Figures S22–S27). It is worth noting that *SUGP1* and *PLRG1* were not available in the Oncomine databases, hinting that these two genes may have little to do with tumor cell proliferation. In addition, there is no significant difference in the expression of several genes in tumor tissues and normal tissues, including *DHX16*, *FUS*, *DHX15*, *NAA38*, and *SKIV2L2* [12].

### 2.7. Protein Modeling

In order to construct the protein structure of the hub genes, 3D protein structures of hub genes were modeled with the Modeller (9v21) with multi-template-based protein modeling approaches. For all proteins, the 3D protein models were generated by homology modeling (except for SUGP1, FUS, HNRNPR, and NAA38) (Table 4). The best model for each protein with the lowest DOPE score (DHX16: −55625.39453; DHX15: −92022.80469; SKIV2L2: −118925.88281; and PLRG1: −41182.91406) was selected for further investigation.

Given the unsatisfactory data from NCBI Blast alignment analysis, the 3D models of four DEGs (SUGP1, FUS, HNRNPR, and NAA38) were modeled de novo with the Rosetta Macromolecular Modeling software package V3.9. One thousand candidate models were constructed for each protein, and model with the lowest score (SUGP1: −51.651; FUS: −116.163; HNRNPR: −96.081; and NAA38: −106.23) was chosen as the best one for the following molecular dynamics (MD) simulation study.

**Figure 5:**
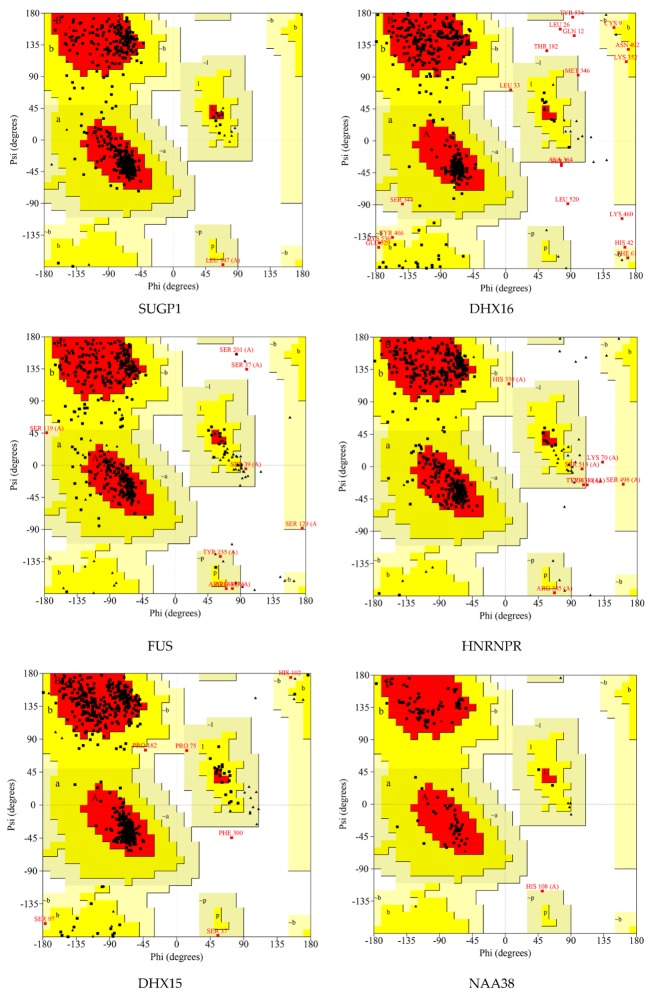

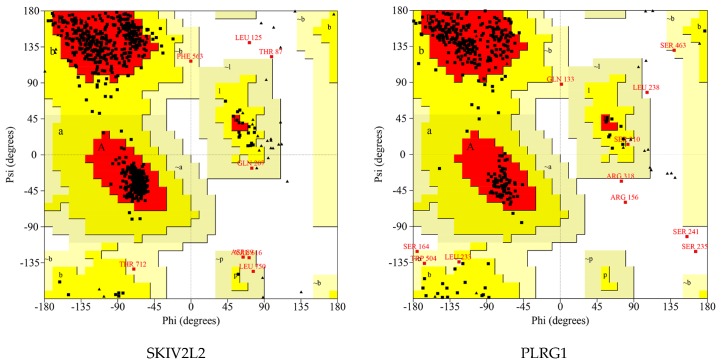
Ramachandran plot of the selected homology modeled 3D protein structures of hub genes. The different colored areas show ‘disallowed’ (beige), ‘generously allowed’ (yellow) and ‘most favored’ (red) regions.

Ramachandran plots were drawn to evaluate the quality of protein models. As shown in Figure 5 and Table 5, these residues existed in outlier regions and ranged from 0% to 2.0% (SUGP1 0.0%, DHX16 2.0%, FUS 0.6%, HNRNPR 0.5%, DHX15 0.1%, NAA38 0.0%, SKIV2L2 0.2%, and PLRG1 1.4%), suggesting that the modeling quality of the protein is good.

### 2.8. Molecular Dynamics and Simulation

**Figure 6:**
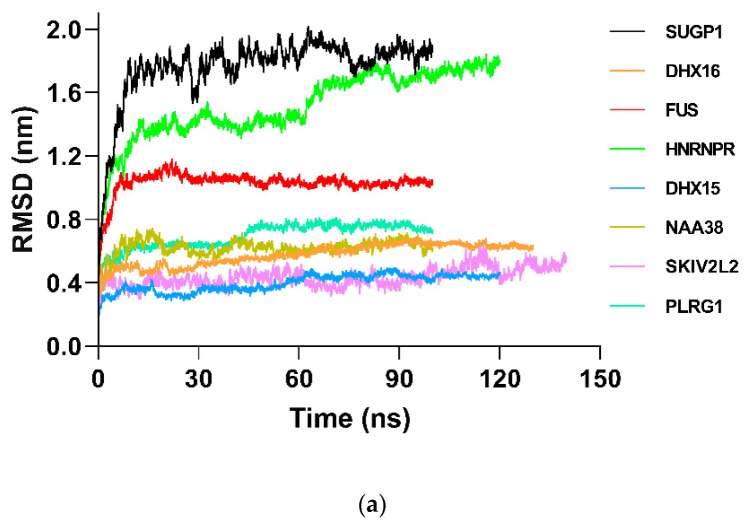

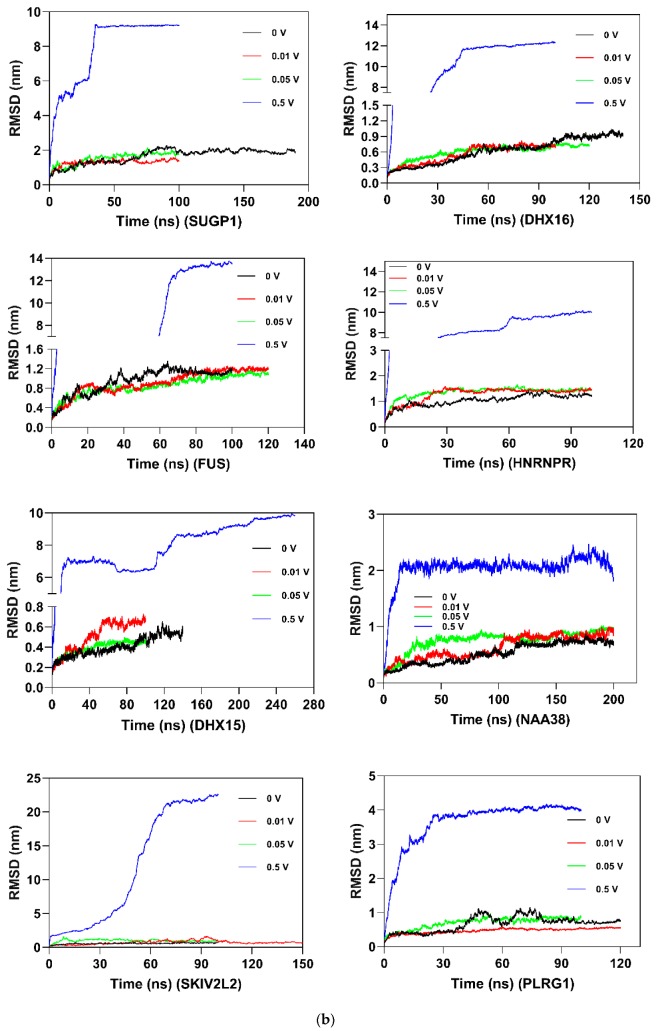
Root mean square deviation (RMSD) comparison plots of backbone Cα atoms during molecular dynamics simulation (at least >100 ns). In order to show the deviations of hub proteins clearly, the RMSD plots are shown. (**a**) The RMSD of each hub protein under molecular dynamics (MD) of “protein in water”. (**b**) The RMSD of selected hub proteins under MD of “protein under nsPEFs”. For each protein, 0 V (black), 0.01 V (red), 0.05 V (green), and 0.5 V (blue) are displayed on one map.

In order to further study the structural behavior of hub gene proteins on a microscopic scale, 100 ns MD simulation was performed for each protein. The structural convergence included root mean square deviation (RMSD) (Figure 6), root mean square fluctuation (RMSF) (Figure S28), and gyrate radius (Figure S29).

RMSD analysis was used to investigate the structure and dynamics of proteins. For the non-nsPEF treatment groups (Figure 6 and Table S1), backbone atoms started initial equilibration up to 5 ns (SUGP1 4.9 ns, DHX16 5.48 ns, FUS 5.36 ns, HNRNPR 5.16 ns, DHX15 5.12 ns, NAA38 5.36 ns, SKIV2L2 1.08 ns, and PLRG1 7.76 ns), and the protein structure started to converge after different times (SUGP1 5.00 ns, DHX16 5.50 ns, FUS 5.40 ns, HNRNPR 5.30 ns, DHX15 5.20 ns, NAA38 5.40 ns, SKIV2L2 1.10 ns, and PLRG1 7.80 ns). Subsequently, models showed stable conformation with an RMSD until the end of the MD production run (SUGP1 1.27–1.97, DHX16 0.48–0.68, FUS 0.98–1.18, HNRNPR 1.14–1.80, DHX15 0.32–0.44, NAA38 0.61–0.71, SKIV2L2 0.38–0.63, and PLRG1 0.58–0.75). For the nsPEF treatment groups, nsPEF application changed the protein atom movements, and this change enhanced with the increase of nsPEF intensity. Hence, these data suggest that the nsPEF markedly affects the atomic motion of the protein in a current intensity-dependent way.

The RMSF of the atoms of proteins investigated the flexibility of the protein (Figure S28 and Table S2). The low RMSF values of residues depicted less flexibility, whereas high RMSF values indicated more motions during simulation in relation to their average position. For the non-nsPEF treatment groups, the average RMSF value of hub proteins was from 0.2165 to 0.6416 (SUGP1 0.6416, DHX16 0.2550, FUS 0.3450, HNRNPR 0.6293, DHX15 0.2026, NAA38 0.2504, SKIV2L2 0.2165, and PLRG1 0.2569). For the nsPEF treatment groups, the average RMSF value for hub proteins of 0 V varied from 0.2220 to 0.4874 (DHX16 0.3628, FUS 0.4874, DHX15 0.2220, and PLRG1 0.3955), and the RMSF values of the cells showed a tendency to decrease after treatment of 0.01 V and 0.05 V. Interestingly, the RMSF value of the 0.5 mV/mm group increased markedly to 0.51–13.20, showing a significant decrease of the simulation system. Similar to RMSD, nsPEF stimulation changed the fluctuation of the protein residues, and the atomic fluctuation amplitude enhanced with the increase of the nsPEF. Overall, these data hinted that the nsPEF-induced protein atoms fluctuation was enhanced with the increase of nsPEF intensity.

The radius of gyration (R*g*) showed the level of compactness of the protein structure. R*g* values reduction indicated enhanced system stability (Figure S29 and Table S3). For the non-nsPEF treatment groups, the average R*g* value of hub gene proteins was from 1.473 to 3.436 (SUGP1 3.436, DHX16 2.512, FUS 2.646, HNRNPR 2.911, DHX15 2.945, NAA38 1.473, SKIV2L2 3.251, and PLRG1 2.235). For the nsPEF treatment groups, the average R*g* value for hub gene proteins of 0 V was from 2.414 to 2.999 (DHX16 2.662, FUS 2.641, DHX15 2.999, and PLRG1 2.414), and the R*g* values of the cells had a tendency to decrease after 0.01 V and 0.05 V exposure. It is worth noting that for the 0.5 mV/mm simulation group, the R*g* value increased significantly to 4.58–14.74, indicating an obvious decrease in the stability of the simulated system.

**Figure 7:**
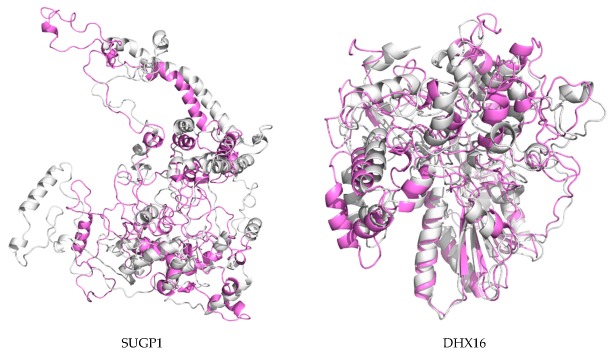

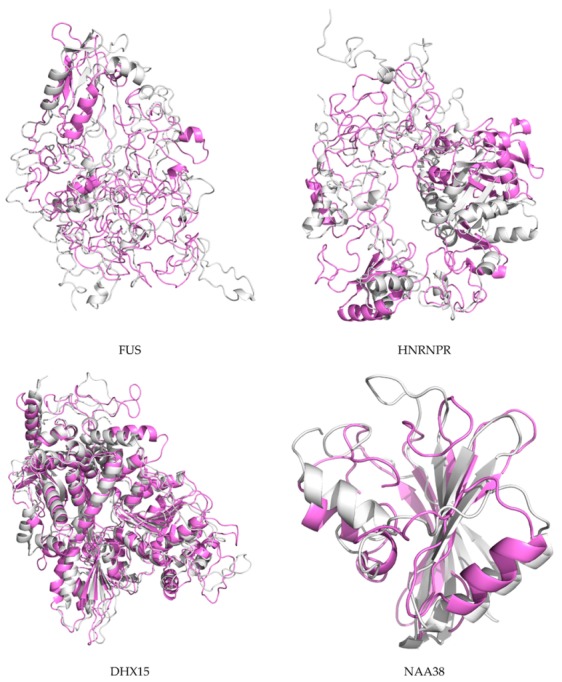

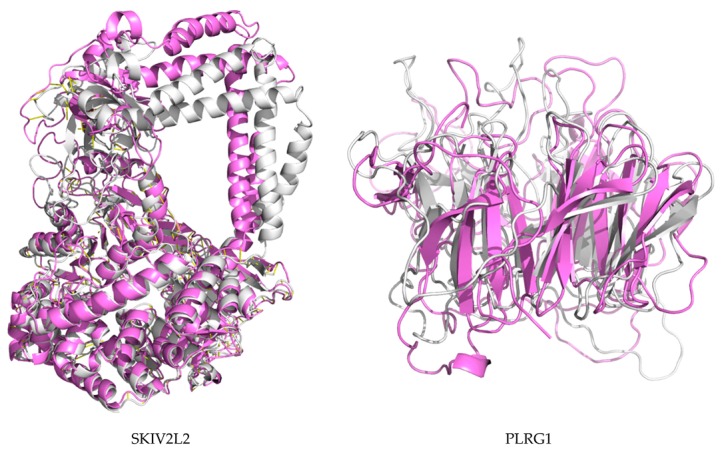
Superposition of the primarily modeled structure (gray) and the MD-optimized protein structure (violet). Yellow: partially mixed area.

**Figure 8:**
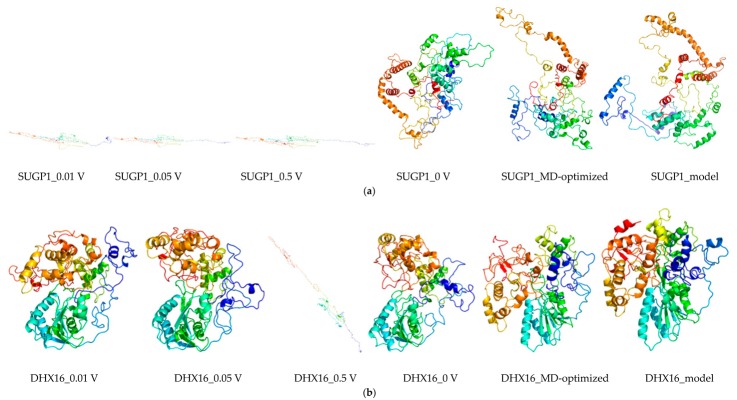

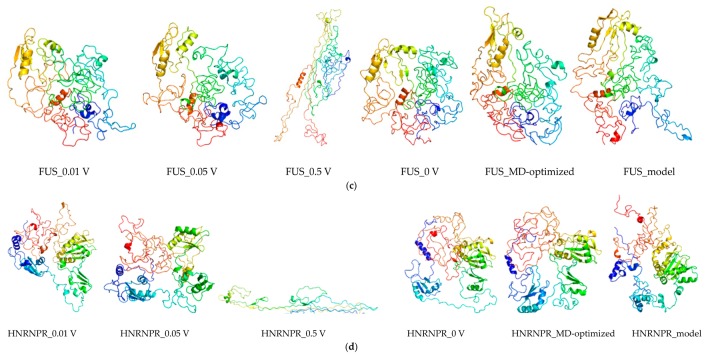

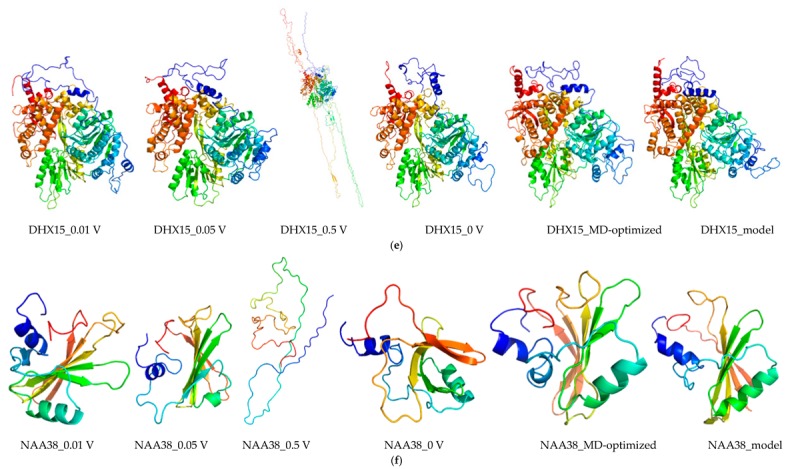

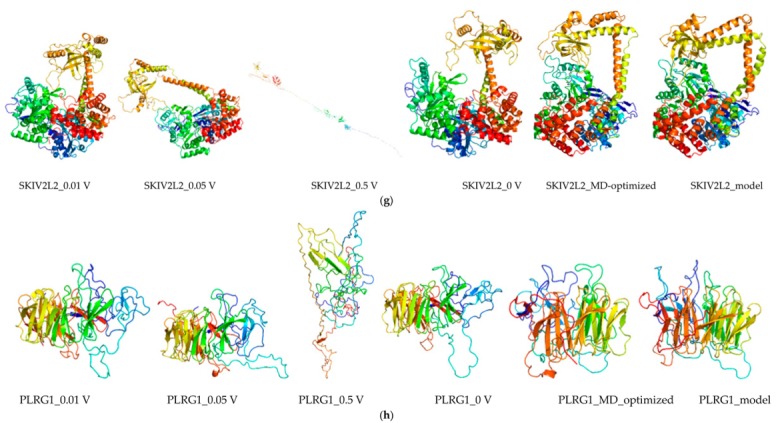
The structure of the 3D protein of hub proteins optimized by molecular dynamics. (**a**) SUGP1_model: the three-dimensional structure of the SUGP1 protein obtained by modeling; SUGP1_MD-optimized: after at least 100 ns molecular dynamics simulation, the lowest energy protein conformation of SUPG1 protein was obtained (based on the three-dimensional structure of the primary modeling) and was subsequently used for subsequent molecular dynamics simulations. After simulation of different electric field conditions, including 0 V (SUGP1_0 V), 0.01 V (SUGP1_0.01 V), 0.05 V (SUGP1_0.05 V) and 0.5 V (SUGP1_0.5 V), the lowest energy protein of SUGP1 protein were obtained respectively. Other proteins (**b**–**h**) were treated similarly to the SUPG1 protein. The pictures were drawn by the Visual Molecular Dynamics (VMD) software and the color map of the protein structure was shown in terms of protein secondary structure.

The 3D models of MD-optimized protein models were shown in Figure 7, Figure 8, Figure S30, and Table S4, with RMSD of origin protein models and MD-optimized models. For most proteins, an increase in current caused a significant increase in the structural changes of the protein. Collectively, these data showed that the stability of the protein was gradually decreased as the nsPEF enhanced.

## 3. Discussion

With the increasing incidence of leukemia, this disease is currently considered to have a largely unmet medical treatment requirement. At present, various methods including bioinformatics are used to explore the treatment of leukemia and have made a series of advancements. In this study, we used a series of bioinformatics and molecular dynamic methods to investigate the effects of nsPEF on a kind of acute T-cell leukemia cell strain-Jurkat, especially its signal pathway. Although this study only provides an exploration of the effects of nsPEF on Jurkat cells from the perspective of molecular informatics, we believe that this study is still worthy of attention in the following aspects.

### 3.1. Electric Field: Jurkat Cells

The Jurkat cell was originally isolated from the peripheral blood of a boy suffering from T-cell leukemia [13]. Due to their good model role in leukemia and T cells, Jurkat cells have been used in the study of the biological effects of electric fields, and interesting advances have been made. Keiko Morotomi-Yano et al. show that exposure to nanosecond pulsed electric fields (nsPEFs) (1 Hz) results in apoptosis of Jurkat in an extracellular calcium-independent way, whereas leukemia cells K562 cells undergo necrosis-associated poly(ADP-ribose) (PAR), hinting that nsPEFs induce cell leukemia cell death in a cell type-dependent manner [14]. Diganta Dutta et al. have made systematic contributions for this field. By treating the Jurkat cells with a single nsPEF of low (15 kV/cm) and high (60 kV/cm), they found that the action cytoskeleton plays a critical role in cellular morphology [15]. Moreover, they showed that nsPEF treatment changes the cell surface charge density of Jurkat cells [16]. In addition, they also mapped the energy dissipation of Jurkat cells by using a single 60 nsPEF treatment [17]. Recently, Esin B. et al. demonstrated that high-intensity nsPEF (10 mV/m, 5 ns) permeabilizes the membrane of Jurkat cells with pores of 0.7–0.9 nm, suggesting that nsPEF can effectively kill leukemia cells [18]. Collectively, nsPEF comprehensively affects many aspects of leukemia Jurkat cells and can effectively kill tumor cells, which provides a valuable experimental basis for an in-depth understanding of the effects of nsPEF in Jurkat cells.

### 3.2. Electric Field: Jurkat Cell Signal Pathway Change

Unexpectedly, shifts in signal pathways were observed after Jurkat cells were stimulated with nsPEF for 30 and 60 min. As shown in Table 1, after being stimulated by nsPEF for 30 and 60 min, the signaling pathways of the down-regulated gene were mostly the same. Only the signal pathway “DNA geometric change” at 30 min was changed into “regulation of cellular response to stress” at 60 min, which might be the response of the Jurkat cells to the “stress” of nsPEF exposure.

In contrast, the signaling pathways activated by the up-regulated genes after 30 and 60 min of nsPEF treatment were significantly altered. The transformation of this signaling pathway was mainly changed from the biochemical pathway at 30 min (glucose homeostasis immune, response-regulating cell surface receptor signaling pathway) to the gene transcriptional pathway and the cell cycle pathway at 60 min (transcription elongation from RNA polymerase II promoter, negative regulation of cell cycle). These observations suggest that the Jurkat cells may respond to nsPEF stimulation in this order: First, the regulation of biochemical metabolic pathways occurs, followed by regulation of gene expression and adjustment of growth cycle. To the best of our knowledge, no studies have been performed on the changes of signaling pathways in nsPEF-stimulated cells. This unexpected observation may provide a clue to the subsequent study of the complex effects of nsPEF on cells.

*PTEN* (Phosphatase and Tensin Homolog) is considered as a multi-functional tumor suppressor that is very commonly mutated (lost) in a large number of tumors at high frequency (leukemia, melanoma, colorectal, breast, lung, thyroid, and bladder). The protein encoded by *PTEN* is phosphatidylinositol-3,4,5-trisphosphate 3-phosphatase, which contains a tensin-like domain as the catalytic domain similar to that of the protein tyrosine phosphatases. *PTEN* regulates the PI3K/AKT activation and TCR signaling pathway [19].

To date, accumulating evidence shows that *PTEN* functions are of fundamental importance in modulating physiological processes in healthy T cells. In healthy individuals, *PTEN* negatively regulates the PI3K/AKT pathway and maintains moderate T cell proliferation. Meanwhile, in patients with T-ALL, loss of *PTEN* results in sustained activation of the PI3K/AKT pathway and eventually leads to leukemia. Therefore, in various therapeutic mechanisms of leukemia, increasing *PTEN* levels is beneficial for the treatment of T-ALL patients [20,21].

In this study, the signaling pathway of *PTEN* was up-regulated after nsPEF stimulation (30 min), suggesting that nsPEF stimulation may increase the *PTEN* level on Jurkat cells. This result may have positive implications for the treatment of T-ALL leukemia. Interestingly, the expression of *PTEN* pathway returned to normal after 60 min of nsPEF stimulation, hinting that the *PTEN* pathway in Jurkat cells recovered. It should be noted that, due to the lack of experimental validation in this study, the above results only provide a clue for subsequent research.

In addition, because the functions of the “general pathway” are too broad, we have not found a specific consistency between the up-regulated and down-regulated pathways, indicating that the results of the function and pathway enrichment analysis may require subsequent experiments to verify. In our opinion, the enrichment and analysis of the signal pathway itself is a preliminary analysis with high throughput and high background noise, which can only provide clues for further experimental research on the microarray data.

### 3.3. Electric Field: Hub Gene Analysis

Electrochemical therapy for leukemia has been regarded as a novel method of cancer therapy; however, its molecular mechanism is less understood and deserves further exploration. Here, by using the expression data from nsPEF-treated Jurkat cells (GSE4106), we investigated the effects of nsPEF on transcriptomics of leukemia cells and their underlying signaling pathways.

In this study, a total of 1769 DGEs were obtained. After a series of integrated bioinformatics analysis, eight hub genes were obtained: *SUGP1*, *DHX16*, *FUS*, *HNRNPR*, *DHX15*, *NAA38*, *SKIV2L2*, and *PLRG1* (Table 6 and Table 7).

*SUGP1* is 2566 bp in length and is located on chromosome 19. As a protein-coding gene, *Fcgr1* encodes a protein called SURP and G-patch domain containing 1. *SUGP1* is essential for lipid and cholesterol metabolism in the liver. Kim, M. J. et al. showed that *SUGP1* contributes to the splicing of rs10401969 and blood lipids through alternate regulation, hinting that *SUGP1* would be a novel modulator in cholesterol metabolism [22]. In the present study, electric field exposure increased the expression of *SUGP1* in Jurkat cells 25.32-fold compared to the untreated group (Supplementary File 1), suggesting that *SUGP1*-mediated cholesterol metabolism may be involved in the signal response of Jurkat cells to the pulsed electric field.

*DHX15* and *DHX16* belong to a family called DEAD-box proteins, which anticipate in a series of cellular processes including alteration of ribosome RNA, spliceosome, and assembly secondary structure. *DHX15* and *DHX16* are located on chromosomes 4 and 6, respectively. They encode two different proteins: DEAH-box helicase 15 and DEAH-box helicase 16.

*DHX15* is involved in the regulation of tumor cell growth and is thereby identified as a coactivator of prostate tumor progression [28]. In addition, *DHX15* activation plays a positive role in bone defect regeneration [29]. *DHX16* has been found to be involved in human pre-mRNA splicing [23]. Moreover, in the nuclei of human cells, mutant *DHX16* induces a defective spliceosome to keep unspliced gene transcripts, hinting that *DHX16* plays a key role in gene expression [24]. In our data, nanosecond pulsed electric field exposure provoked significant increases of *DHX15* and *DHX16* expression in Jurkat cells, indicating that nanosecond pulsed electric fields treatment may affect the gene expression of leukemia cells.

*FUS* is 5119 bp in length and is located on chromosome 16, which encodes a protein called *FUS* RNA binding protein. Like *DHX16*, *FUS* plays a role in different cellular processes, including DNA repair, RNA splicing, transcription regulation, damage response, and RNA transport. *FUS* has long been considered as a key player in neuronal cell-related diseases, and a recent study revealed that FUS protein mislocalization in amyotrophic lateral sclerosis (ALS) could be an underlying mechanism for this disorder [25]. Here, nanosecond pulsed electric fields exposure causes Jurkat cells *FUS* levels to increase at least 65.23-fold, indicating that current stimulation may affect the differentiation of leukemia cells.

*HNRNPR*, a member of the heterogeneous nuclear ribonucleoprotein family, is an RNA-binding protein [26]. *HNRNPR* plays a pivotal role in processing the pre-mRNA in the cell nucleus, and *HNRNPR* mutation could cause multi-system congenital disorders [27]. In recent years, the role of *HNRNPR* in the immune system has also received attention. For example, *HNRNPR* is identified as a general positive modulator of MHC class I expression [26]. In our study, the transcription level of *HNRNPR* in leukemia cells was significantly increased after treatment with nsPEF, suggesting that the nsPEF may affect the immune differentiation of Jurkat cells.

*NAA38* is located on chromosome 17 and has a length of 999 bp. It encoded the protein of N (alpha)-acetyltransferase 38. Until now, there have been fewer reports on the biological function of *NAA38*, and it is believed that *NAA38* is related to the pathways of Golgi-to-ER retrograde transport and vesicle-induced transport [30].

*SKIV2L2* is 3795 bp in length and is located on chromosome 6, and it encodes a protein called Ski2-like RNA helicase 2. *SKIV2L2* also belongs to the DEAD-box protein family, which is implicated in cellular processes such as alteration of spliceosome assembly, RNA structure and ribosome. The report shows that *SKIV2L2* deficiency hinders mitotic progression and histone mRNA turnover in mouse cells, suggesting that *SKIV2L2* regulates the cell proliferation [31].

*PLRG1* is located on chromosome 4 and has a length of 3317 bp, which encodes a protein called pleiotropic regulator 1 (*PLRG1*). *PLRG1* plays critical roles in gene expression, such as alternative splice site selection, and is considered as a critical nuclear modulator of p53-dependent cell cycle apoptosis and progression [33]. At the molecular level, *PLRG1* interacts directly with the hnRNA-M protein and hence regulates the alternative splice site selection of gene expression [32]. In our study, nanosecond pulsed electric fields induced obvious *PLRG1* gene expression increase, indicating that nanosecond pulsed electric fields may affect the cell proliferation of leukemia cells.

In recent years, a series of advances have been made in the effects of nsPEF on proteins. The Stacey L. Martens team used adult human dermal fibroblasts as a cellular model to study the effects of nsPEF on cells. After stimulation of cells with nsPEF (100, 10 ns pulses at 150 kV/cm, 1 Hz), the mRNA of the endoplasmic reticulum stress gene did not change. Moreover, the intracellular unfolded protein response (UPR) protein also did not alter [34]. On the contrary, in the present study, these above eight hub genes of Jurkat cells have significant responses to nsPEF stimulation. These above findings hinted that the effects of nsPEF on different types may not be the same and may vary depending on the cell types.

### 3.4. Electric Field: Jurkat Cell Gene Expression Change

Regarding the reasons why nsPEF were capable of changing gene expression of cells, to the best of our knowledge, there is no specific study on this aspect yet. In this study, we speculated that the following reasons may be responsible for it.

Firstly, nsPEF may affect the replication and transcription of genes within cells. The mechanical factor is involved in the normal replication and transcriptional processes of DNA. For example, the DNA helicases unwind the double strands of DNA so that the DNA replication can proceed normally [35]. Therefore, the force generated by nsPEF may interfere with the process, which in turn alters the expression of certain genes.

Secondly, nsPEF may alter the intracellular solution ion environment. The cells are in a dynamically balanced electrolyte steady state environment, which is required for normal replication and transcription of the gene. In the present study, nsPEF may interfere with the normal expression of the gene by altering the ions and charges in the cell solution. We sincerely hope that the follow-up experimental research will help to solve this question.

### 3.5. Electric Field: The Effects of nsPEF on Cancer Cells

Currently, nsPEF is developing rapidly throughout the field of cancer treatment. In the case of breast cancer, the combination of nsPEF and low-dosage paclitaxel enhances the killing effect on breast cancer cells MDA-MB-231 [36]. In glioma, glioma cell line U87 is very sensitive to nsPEF treatment [37]. For the squamous carcinoma, compared to the sham and single-train treatments groups, tumors being sensitized with nsPEF (150 + 150, 300 ns pulses, 6.4 kV, 20 Hz) lead to a four- and two-fold tumor volume reduction in mice, respectively, which shows that nsPEF could be developed to assist in vivo cancer treatment [38]. For lung cancer and liver cancer, nsPEF (40 kV/cm, 500 pulses at 1 Hz) suppresses lung metastasis on both murine transplanted hepatocellular tumors and canine spontaneous osteosarcoma [39]. Collectively, nsPEF is a promising non-thermal therapeutic anti-tumor approach in pre-clinical studies.

### 3.6. The Limitations of This Study

First of all, this study only performed an in silico analysis of the response of a leukemia cell strain to nsPEF, which has limited implications for the understanding of leukemia as a whole. Due to the complexity of leukemia classification, we should be consciously aware that the progress of basic research in the treatment of a subtype of leukemia does not mean that the method may be effective against other subtypes of leukemia. At the level of mechanism study, the underlying molecular events that contribute to different types of leukemia are not the same. Furthermore, different leukemia cell lines do not respond the same to one treatment (such as nsPEF).

Secondly, in silico data provide limited reference value for real clinical studies and requires further experimental validation. In this study, on the one hand, we have difficulty in contacting the original experimental group and requesting the experimental samples; on the other hand, the specific device for generating nanosecond pulses is a highly professional job. Therefore, we have not been able to further conduct biological validation in the study. As an in silico study, from the perspective of molecular informatics, our study sought to explore changes in the gene expression profile of nanosecond pulses-treated Jurkat cells and attempted to analyze their underlying mechanism, which was intended to provide some insights into the understanding of Jurkat cells’ response to external current stimuli. Overall, the analysis results obtained in this study still require a large amount of experimental data to verify.

Thirdly, changes in mRNA levels provided by gene chips do not accurately reflect changes in final protein levels because gene expression includes multiple levels of regulation, such as transcription, post-transcriptional regulation, translation, and post-translational regulation, especially as intracellular membranes are disrupted and RNA is no longer correctly compartmentalized, hence the gene chip can only provide the expression of the gene at the mRNA level. Therefore, the corresponding detection of protein levels is still needed to support in silico analysis of protein levels. From the perspective of molecular informatics, our work attempted to analyze the characteristics of hub proteins, which may provide a preliminary reference for subsequent experimental research.

Finally, the accuracy of gene chip technology in detecting gene expression levels remains to be further developed. Since its invention, gene chip technology has been widely used for its advantages of fastness, efficiency, sensitivity, and high throughput. However, since the physiological state of the biological sample is inevitably affected or even destroyed during the preparation of the biological samples, the ability of gene chip technology in detecting the gene expression of cells in the natural condition still needs further improvement. Collectively, in actual clinical research, gene chip detection should be used in conjunction with other technologies to ensure that accurate information is acquired.

## 4. Materials and Methods

### 4.1. Main Steps

Briefly, this study mainly included the following three steps:

Step 1: The nsPEFs-sensitive genes were acquired from Jurkat cells by using the R limma package, followed by comprehensive bioinformatics analysis to investigate these hub genes’ signaling mechanisms. In brief, the expression data (GSE4106) were acquired from the Gene Expression Omnibus (GEO, http //:www.ncbi.nlm.nih.gov/geo/), and 12 samples were divided into 2 groups (30 min treatment group and 60 min treatment group) for subsequent analysis. Next, the differentially expressed genes (DEGs) were obtained with the GEO2R tool, followed by gene ontology (GO) and pathway enrichment analyses. Then, PPI networks were produced by STRING (http://string-db.org/), Cytoscape, and FunRich software, and 8 hub genes were finally obtained. Finally, by analyzing the 8 hub genes with various tools including the Human Protein Atlas, Oncomine, Kaplan–Meier, and cBioPortal, the possible mechanism of leukemia cells under nsPEFs was studied.

Step 2: The three-dimensional protein structure of the 8 hub genes was generated. With the help of Modeller 9.21 and Rosetta 3.9, three-dimensional structural models for all hub proteins were originally screened out. Then, a 100 ns molecular dynamics simulation for each hub protein was performed with the GROMACS 2018.2 software package [40], and the optimal kinetic protein structures of hub proteins were finally established.

Step 3: Molecular dynamics (at least 100 ns) for 8 hub proteins were conducted to simulate the molecular motion at three nsPEFs strengths (0.01, 0.05, 0.5 mV/mm). Subsequently, a series of molecular motion characteristics of hub proteins was acquired, such as RMSF, RMSD, and gyration radius of atoms.

### 4.2. Microarray Data

Gene expression profiling of GSE4106 was obtained from the publicly available GEO database (https://www.ncbi.nlm.nih.gov/geo/). According to whether Jurkat cells were stimulated by nsPEFs, the cells were divided into nsPEFs treatment group (experimental group) and untreated group (control group). Each group contained 6 samples, and each time point contained 3 biological replicates. The gene expression of Jurkat cells after 30 and 60 min of nsPEFs exposure was studied.

### 4.3. Identification of DEGs

After obtaining these raw data from the platform of GPL2895, the limma package in R [41] was used to investigate significant DEGs by linear models. After the *t*-test, only those DEGs with a *p* < 0.05 were selected, and a |logFC| > 0.5 needed to be met. The analysis classified the DEGs into two groups with distinct expression profiles. DEGs were ordered based on the fold change (LogFC) in the expression value. This information was demonstrated as a heat map plot (Figure 2).

### 4.4. Functional Enrichment Analysis for DEGs

To interpret the specific functions and pathways associated with DEGs, genes in selected modules were analyzed using the online bioinformatics database Metascape (http://metascape.org/gp/index.html#/main/step1) [42]. In brief, DEGs lists were divided into two groups according to different treatments of Jurkat cells (Table 8). Subsequently, the enrichment analysis was carried out with the help of the Metascape online tool. Finally, the PANTHER GO classification system (http://www.pantherdb.org/) [43] was used to investigate the biological functions and pathways of DEGs.

### 4.5. Protein–Protein Interaction (PPI) Network Construction and Module Analysis

The PPIs of DEGs were predicted with the aid of the Search Tool for the Retrieval of Interacting Genes (STRING) database (https://string-db.org/) [44]. Only the minimum required interaction score > 0.4 was chosen as significant. Subsequently, PPI network was performed by Cytoscape software version 3.7.1 (https://cytoscape.org/). The Molecular Complex Detection (MCODE) clustering analysis was performed to screen the modules of PPI network in Cytoscape. In order to exclude relatively small clusters, the parameters were set as follows: MCODE scores > 3 and number of nodes > 4. Furthermore, enrichment analyses were conducted for DEGs in each module. The threshold was set at *p* < 0.05 [45]. To obtain the balance between the DEGs and identify hub genes, the cytoHubba plugin was used to score all proteins in the PPI network by 12 methods (EPC, MCC, DMNC, MNC, Closeness, Degree, BottleNeck, Betweenness, Stress, EcCentricity, Radiality, and Clustering Coefficient) [46,47].

In addition, ClueGO and Cluepedia (http://apps.cytoscape.org/apps/cluego), another plugin program of cytoscape, were used to further reveal the involvement of multiple molecular pathways that may be associated with the identified DEGs. Kappa coefficient of 0.4 and *p*-value of <0.05 were selected as threshold values. In the present study, 100 genes were selected from the two groups according to their *p*-values.

A novel Cytoscape plugin CytoHubba (Ver 0.1) was employed to screen the hub genes by analyzing the interaction between the top 40 genes from 12 topological analysis methods. Both group1 and group2 were analyzed, and 8 genes that were coincident were finally selected as the hub genes.

### 4.6. Exploring Sub-Localization Expression of Hub Genes by Human Protein Atlas

The Human Protein Atlas (HPA) (http://www.proteinatlas.org) is a comprehensive database that provides the protein expression profiles for a variety of human proteins [11]. Immunohistology of most human tissues is presented in images through the online tool. The expression of the hub genes was analyzed when uploading them to HPA separately.

### 4.7. Exploring Jurkat Genomics Data by cBio Cancer Genomics Portal

The cBio Cancer Genomics Portal (http://cbioportal.org) is a free platform that provides a flexible yet straightforward interface to intuitive visualization options, integrated datasets and a programmatic web interface, all of which can help researchers in translating cancer genomic data into potential clinical and biologic insights [48].

With the cBio Portal, the hub genes were explored across the genetic databases of several cancer-related studies [49]. In the present study, using the portal search function, all of the hub genes were classified as altered or not altered. At the same time, the relative expression level of the hub genes were also presented.

### 4.8. The Hub Genes Analysis by Using the Oncomine Database

The Oncomine database (www.oncomine.org) is a publicly accessible online database and web-based data mining platform for enabling discovery from genome-wide expression analyses [50].

To determine the expression pattern of hub genes in Jurkat cells, the mRNA expression profiles of Jurkat cells were identified by comparing major types of cancer vs. normal analysis. Statistical analysis was performed with the Oncomine algorithms. Statistical calculations and details of standardized normalization techniques were provided by using the web-based Oncomine platform [51].

### 4.9. Protein Modeling

Protein sequences were obtained from the NCBI nucleotide database (https://www.ncbi.nlm.nih.gov/protein), and the Blast module was used to align the protein sequences with the PDB database [52]. For every protein, three templates (query cover > 30%) were selected as templates for subsequent homology modeling (Table 9). Next, 3D homology models of hub proteins were generated with the Modeler (9v21) [53]. Multiple-template modeling approaches including Salign, Align2d, and Model modules were utilized for the modeling process. One thousand candidate models of each protein were produced, and the best model was acquired based on scores calculated from discrete optimized protein energy (DOPE).

### 4.10. De Novo Modeling of Proteins

Considering that the blast results of several proteins (SUGP1, FUS, HNRNPR, and NAA38) from NCBI (query cover < 30%) are too low to generate qualified protein models by using Modeller (9v21), the ROSETTA3.9 (de novo modeling script) [54] was used to construct 3D protein models for these proteins. One thousand candidate models for each protein were generated and the one with the lowest score was chosen as the best theoretical protein model.

### 4.11. Molecular Dynamics Simulations

MD simulation of the hub proteins was performed with the GROMACS2018.2 package [40] in Linux environment.

#### 4.11.1. Molecular Dynamic Simulation: Protein in Water

Molecular dynamics simulation was run with the Amber99SB ILDN force field [55] force field, and the simulation for proteins was carried out at the similar condition with various minor modifications. Proteins were fully solvated in an octahedron box with simple point charge (SPC) water molecules (1.0 nm). The system was neutralized by adding Na^+^ or Cl^−^ ions, and periodic boundary conditions were used in all directions. Energy minimization of the protein was conducted with 50,000 steps of steepest descent with max force (less than 100 KJ/mol). Subsequently, the equilibration phase was started by using NVT (50 ps, 300 K) and NPT (100 ps, 300 K, 1.0 bar) respectively. Molecular dynamics simulation was conducted at least 100 ns for every protein.

#### 4.11.2. Molecular Dynamic Simulation: Protein under nsPEFs

The molecular dynamics simulation was performed with the CHARMM36 force field [56], and at least 100 ns was simulated in the NPT stage. The nsPEF limiting condition of different voltages (0.01, 0.05, and 0.5 mV/mm) was set to mimic the nsPEF, and other conditions remained the same as “protein in water”.

#### 4.11.3. Molecular Dynamic Simulation Analysis

The MD trajectory of MD simulation was analyzed with GROMACS utilities to obtain the RMSF, RMSD, and radius of gyration. Xmgrace tool was utilized to draw various plots. Ramachandran plots were drawn with PROCHECK Ramachandran plots [57] (http://www.ebi.ac.uk/thornton-srv/databases/pdbsum/Generate.html). The 3D protein structure of protein models was produced by the Visual Molecular Dynamics (VMD) software [58].

## 5. Conclusions

In the present study, with the help of bioinformatics and molecular dynamics, we explored the signal pathways of Jurkat cells in response to nsPEF-like physical stimuli. NsPEF caused significant changes in the signaling pathway of Jurkat cells. Further studies showed that the effect of nsPEF on the signaling pathway of Jurkat cells presented two characteristics: The up-regulated signaling pathways varied with stimulation time, whereas the type of down-regulated signaling pathways was basically unchanged. Moreover, the *PTEN* signaling pathway was up-regulated after nsPEF stimulation (30 min), which hinted that nsPEF stimulation for 30 min may have a growth-inhibiting effect on Jurkat cells. In addition, the 3D structure of the hub proteins was modeled and optimized by molecular dynamics to obtain the lowest energy conformation of these hub proteins, which laid the foundation for the development of potential inhibitors.

Collectively, from a molecular information perspective, our data explored the effects of nsPEF on the cellular processes of Jurkat cells, which not only did a meaningful exploration for the in-depth understanding of T-ALL leukemia cells in response to external physical stimuli (such as nsPEF) but also provided promising clues for the clinical therapy of T-ALL patients in the future.

## Figures and Tables

**Table 1:** Functional and pathway enrichment analysis of identified modules associated with DEGs.

Pathway Type (Up-Regulated)	Up-Regulation (Group 1)	Up-Regulation (Group 2)	Pathway Type (Down-Regulated)	Down-Regulation (Group 1)	Down-Regulation (Group 2)
General (biological process—BP)	negative regulation of cell proliferation	negative regulation of cell proliferation	General (BP)	metabolism of RNA	metabolism of RNA
Specific (BP)	PID NFAT TF pathway	PID NFAT TF pathway	General (BP)	cell cycle	cell cycle
General (molecular function—MF)	regulation of double-strand break repair via homologous recombination	regulation of double-strand break repair via homologous recombination	General (MF)	ncRNA metabolic process	ncRNA metabolic process
General (MF)	negative regulation of phosphate metabolic process	negative regulation of phosphate metabolic process	General (BP)	DNA repair	DNA repair
General (BP)	chromatin remodeling	chromatin remodeling	General (BP)	nuclear export	nuclear export
General (MF)	maintenance of cell number	maintenance of cell number	General (MF)	cell cycle phase transition	cell cycle phase transition
Specific (BP)	O-glycan processing	T cell mediated cytotoxicity	General (MF)	regulation of chromosome organization	regulation of chromosome organization
General (BP)	mRNA processing	regulation of blood pressure	General (MF)	tRNA metabolic process	tRNA metabolic process
Specific (BP)	regulation of PTEN gene transcription	histone modification	General (BP)	mitotic sister chromatid segregation	mitotic sister chromatid segregation
General (MF)	glucose homeostasis	dephosphorylation	General (BP)	DNA replication	DNA replication
General (MF)	immune response-regulating cell surface receptor signaling pathway	transcription elongation from RNA polymerase II promoter	General (cellular component—CC)	microtubule cytoskeleton organization	microtubule cytoskeleton organization
		negative regulation of cell cycle	General (BP)	covalent chromatin modification	covalent chromatin modification
			General (BP)	organelle biogenesis and maintenance	organelle biogenesis and maintenance
			General (MF)	DNA-templated transcription, termination	DNA-templated transcription, termination
			General (CC)	macromolecule methylation	macromolecule methylation
			General (BP)	organelle localization	organelle localization
			General (BP)	ribonucleoprotein complex biogenesis	ribonucleoprotein complex biogenesis
			General (BP)	cell cycle	cell cycle
			General (BP)	regulation of DNA metabolic process	regulation of DNA metabolic process
			General (BP)	DNA geometric change	regulation of cellular response to stress

For up-regulated/down-regulated genes, the table background colors for the different signal pathways at both 30 min and 60 min were noted.

**Table 2:** Kyoto Encyclopedia of Genes and Genomes (KEGG) pathway analysis of differentially expressed genes associated with leukemia.

Groups	Expression	Pathway ID	Name	Gene Count	%	Genes
Group 1	Down-regulated	hsa01100	Metabolic pathways	117	9.04	*NAMPT, INMT, CHKA, GPAT4, CTPS1, DGKA, DLAT, DLD, DNMT1, EPRS, ALAS1, EXT1, ACSL3, FASN, FECH, SEPHS2, AKR1B1, GANAB, ACSL6, MLYCD, ETHE1, SHPK, PISD, KDSR, GART, GCNT1, GLDC, GLO1, GMDS, GOT2, GPI, PIGW, RIMKLA, GSTM3, HADHA, HK1, HMGCR, IDI1, INPP4A, ACADVL, STT3A, LSS, LTA4H, MAN2A2, MDH2, MGAT5, MPI, ASNS, NDUFA10, ATIC, NDUFB4, NDUFS1, OAT, ODC1, ACO2, OXCT1, PCCA, SEPSECS, PDE8A, INPP5K, PFKP, PGM1, ATP6V1B2, PIK3CD, PI4KA, PI4KB, PLCG1, PLCG2, PGM2, PPT1, ETNK1, SMPD4, CNDP2, AGK, CMAS, INPP5E, AGPAT4, QARS, BCAT1, RPN1, RPN2, SCD, GALNT11, RBKS, SGSH, SHMT1, SORD, TK2, UCK2, ALDH5A1, GALNT12, ALG9, SCD5, PGAP1, UXS1, PCBD2, ACSS1, DGKZ, DGKD, ALG2, AGPS, B4GALT4, CBS, CDS2, SUCLG2, GMPS, ALDH1A2, ST3GAL5, MTMR2, PAPSS1, CERS5, MARS2, PIGS, PIGB, PGS1, ENTPD4, FIG4*
		hsa05200	Pathways in cancer	37	2.86	*CDK2, RASGRP2, CHUK, CRKL, AKT1, MTOR, GSTM3, MSH6, HDAC2, HSP90AB1, FAS, IKBKB, IL3RA, IL13, JAK1, LAMA5, LAMB1, SMAD2, MLH1, PIK3CD, PLCG1, PLCG2, PRKCA, MAPK9, MAP2K1, STAT5B, TGFBR1, TPR, TRAF1, TRAF3, ZBTB17, CXCR4, AXIN1, RASSF5, CASP9, CUL1, CCNE2*
		hsa03013	RNA transport	27	2.09	*NXF1, RPP30, POP1, EIF2B1, EIF4EBP2, NUP210, GEMIN5, CYFIP2, KPNB1, NUP88, NUP98, NUP133, NUP107, UPF1, ELAC2, TPR, UBE2I, NUP85, FXR1, THOC5, EIF3C, PABPC4, NUP155, EIF5B, TGS1, NUP93, THOC1*
	Up-regulated	hsa01100	Metabolic pathways	8	5.80	*AK2, LCLAT1, PGM2L1, IDS, G6PC2, GALNT14, NT5C1A, H6PD*
		hsa04010	MAPK signaling pathway	5	3.62	*DUSP1, DUSP2, FOS, JUN, STK4, STK4*
		has05200	Pathways in cancer	5	3.62	*FOS, DLL1, IL12A, JUN, STK4*
		hsa05202	Transcriptional misregulation in cancer	4	2.90	*FUS, PBX1, SS18, CCNT1*
Group 2	Down-regulated	hsa01100	Metabolic pathways	114	8.74	*NAMPT, INMT, CHKA, GPAT4, CTPS1, DGKA, DCTD, DLAT, DNMT1, EPRS, ALAS1, EXT1, ACSL3, FASN, FECH, SEPHS2, AKR1B1, GANAB, ACSL6, MLYCD, ETHE1, SHPK, PISD, KDSR, GART, GCNT1, GLDC, GLO1, GMDS, GOT2, GPI, PIGW, RIMKLA, GSTM3, HADHA, HK1, HMGCR, IDI1, INPP4A, ACADVL, STT3A, LSS, LTA4H, MAN2A2, MDH2, MPI, ASNS, NDUFA10, ATIC, NDUFB4, NDUFS1, OAT, ODC1, ACO2, OXCT1, PCCA, SEPSECS, PDE8A, PFKP, PGM1, ATP6V1B2, PIK3CD, PI4KB, PLCG1, PLCG2, PGM2, PPT1, SMPD4, CNDP2, AGK, CMAS, INPP5E, AGPAT4, QARS, BCAT1, RPN1, RPN2, SCD, GALNT11, RBKS, SGSH, SHMT1, SORD, TK2, UCK2, ALDH5A1, GALNT12, ALG9, SCD5, DGLUCY, PGAP1, UXS1, PCBD2, ACSS1, DGKZ, DGKD, ALG2, AGPS, B4GALT4, CBS, CDS2, SUCLG2, GMPS, ALDH1A2, ST3GAL5, MTMR2, PAPSS1, CERS5, MARS2, PIGS, PIGB, PGS1, ENTPD4, FIG4*
		hsa05200	Pathways in cancer	37	2.84	*CDK2, RASGRP2, CRKL, AKT1, MTOR, GSTM3, MSH6, HDAC2, HSP90AB1, FAS, IKBKB, IL3RA, IL13, JAK1, LAMA5, LAMB1, SMAD2, MLH1, PIK3CD, PLCG1, PLCG2, PRKCA, PRKCB, MAPK9, MAP2K1, STAT5B, TGFBR1, TPR, TRAF1, TRAF3, ZBTB17, CXCR4, AXIN1, RASSF5, CASP9, CUL1, CCNE2*
		hsa03013	RNA transport	27	2.07	*PRMT5, NXF1, RPP30, POP1, EIF2B1, EIF4EBP2, NUP210, GEMIN5, CYFIP2, KPNB1, NUP88, NUP98, NUP133, NUP107, UPF1, ELAC2, TPR, UBE2I, NUP85, FXR1, THOC5, EIF3C, PABPC4, NUP155, EIF5B, TGS1, NUP93*
	Up-regulated	hsa01100	Metabolic pathways	9	6.21	*CYP2C9, AK2, LCLAT1, PGM2L1, IDS, G6PC2, GALNT14, NT5C1A, H6PD*
		hsa05200	Pathways in cancer	6	4.14	*FOS, DLL1, IL12A, JUN, STK4, CCNA1*
		hsa04010	MAPK signaling pathway	5	3.45	*DUSP1, DUSP2, FOS, JUN, STK4*
		hsa05166	Human T-cell leukemia virus 1 infection	5	3.45	*EGR1, FOS, JUN, VAC14, CCNA1*

**Table 3:** The distribution of the hub genes in cells.

Gene Name	Cell Lines	Main Location
*SUGP1*	A-431, U-2OS, U-251MG	nucleoplasm
*DHX16*	HeLa, MCF7, U-2 OS	nucleoplasm
*FUS*	A-431, U-2 OS, U-251MG	nucleoplasm
*HNRNPR*	A-431, U-2 OS, U-251MG	nucleoplasm
*DHX15*	A-431, HEK 293, U-2 OS	nuclear speckles
*NAA38*	HEK 293, PC-3, U-2 OS	nucleus
*SKIV2L2*	A-431, U-2 OS, U-251MG	nucleus
*PLRG1*	A-431, U-2 OS, U-251MG	nuclear speckles, nuclear membrane

**Table 4:** Protein modeling.

Proteins	Species	Protein Length (aa)	Model Templates (Query Cover, Identify)
SUGP1	*Homo sapiens*	645	de novo
DHX16	*Homo sapiens*	560	5Z58_XX (94%, 99%) 5MQF_QQ (90%, 56%) 6FA9_A (89%, 55%)
FUS	*Homo sapiens*	522	de novo
HNRNPR	*Homo sapiens*	535	de novo
DHX15	*Homo sapiens*	795	5XDR_A (86%, 99%) 3KX2_B (84%, 66%) 2XAU_A (84%, 66%)
NAA38	*Homo sapiens*	125	de novo
SKIV2L2	*Homo sapiens*	1042	6D6Q_M (100%, 100%) 6C90_A (70%, 100%) 2XGJ_A (89%, 56%)
PLRG1	*Homo sapiens*	514	6FF4_D (100%, 100%) 5MQF_D (100%, 99%) 4YVD_A (72%, 100%)

**Table 5:** Ramachandran plot analysis.

Proteins	Number of Residues in Favored Region	Number of Residues in Allowed Region	Number of Residues in Disallowed Region
SUGP1	448 (93.3%)	32 (6.7%)	0 (0.0%)
DHX16	423 (84.9%)	65 (13.0%)	10 (2.0%)
FUS	284 (82.3%)	59 (17.1%)	2 (0.6%)
HNRNPR	379 (90.2%)	39 (9.3%)	2 (0.5%)
DHX15	658 (92.4%)	53 (7.4%)	1 (0.1%)
NAA38	94 (89.5%)	11 (10.5%)	0 (0.0%)
SKIV2L2	870 (93.2%)	61 (6.5%)	2 (0.2%)
PLRG1	305 (84.3%)	52 (14.4%)	5 (1.4%)

For SUGP1 and PLRG1, part of the N-terminus was removed for subsequent molecular dynamics simulation.

**Table 6:** Electric field-sensitive hub genes.

Name and Ensembl ID	Species Gene Type	Location Length	Function	Refs
*SUGP1* (SURP and G-patch domain containing 1) (ENSG00000105705)	*Homo sapiens* Protein coding	Chr 19 (2566 bp)	A novel modulator in cholesterol metabolism	[22]
*DHX16* (DEAH-box helicase 16) (ENSG00000204560)	*Homo sapiens* Protein coding	Chr 6 (3406 bp)	Involved in the human pre-mRNA splicing	[23,24]
*FUS* (FUS RNA binding protein) (ENSG00000089280)	*Homo sapiens* Protein coding	Chr 16 (5119 bp)	A key player in neuronal cell-related diseases	[25]
*HNRNPR* (Heterogeneous nuclear ribonucleoprotein R) (ENSG00000125944)	*Homo sapiens* Protein coding	Chr 1 (7751 bp)	Is involved in processing the pre-mRNA in cell nucleus identified and is considered as a general positive modulator of MHC class I expression	[26,27]
*DHX15* (DEAH-box helicase 15) (ENSG00000109606)	*Homo sapiens* Protein coding	Chr 4 (2998 bp)	Is involved in the regulation of tumor cell growth, such as prostate cancer progression and bone defect regeneration	[28,29]
*NAA38* (N(alpha)-acetyltransferase 38, NatC auxiliary subunit) (ENSG00000183011)	*Homo sapiens* Protein coding	Chr 17 (999 bp)	Is related to the pathways of Golgi-to-ER retrograde transport and vesicle-induced transport	[30]
*SKIV2L2* (Ski2-like RNA helicase 2) (ENSG00000204351)	*Homo sapiens* Protein coding	Chr 6 (3795 bp)	Regulates the cell proliferation	[31]
*PLRG1* (Pleiotropic regulator 1) (ENSG00000171566)	*Homo sapiens* Protein coding	Chr 4 (3317 bp)	Regulates the cell proliferation	[32,33]

**Table 7:** GO analysis of hub genes.

Gene	GO Analysis [30]
*SUGP1*	MF: nucleic acid binding; RNA binding; protein binding
	BP: mRNA splicing, via spliceosome; RNA processing; mRNA processing; RNA splicing
	CC: nucleus; nucleoplasm; spliceosomal complex
*DHX16*	MF: nucleic acid binding; RNA binding; RNA helicase activity; helicase activity; protein binding
	BP: mRNA splicing, via spliceosome; mRNA processing; RNA splicing
	CC: nucleus; nucleoplasm; spliceosomal complex; U2-type precatalytic spliceosome
*FUS*	MF: nucleic acid binding; DNA binding; chromatin binding; transcription coactivator activity; RNA binding
	BP: mRNA splicing, via spliceosome; regulation of transcription, DNA-templated; regulation of transcription by RNA polymerase II; RNA splicing; regulation of RNA splicing
	CC: nucleus; nucleoplasm; cytoplasm; polysome; dendrite
*HNRNPR*	MF: nucleic acid binding; RNA binding; mRNA binding; mRNA 3’-UTR binding; protein binding
	BP: nucleus; nucleoplasm; spliceosomal complex; NOT nucleolus; cytoplasm
	CC: mRNA splicing, via spliceosome; mRNA processing; circadian rhythm; RNA splicing; RNA metabolic process
*DHX15*	MF: nucleic acid binding; RNA binding; RNA helicase activity; double-stranded RNA binding; helicase activity
	BP: mRNA splicing, via spliceosome; mRNA processing; RNA splicing; response to toxic substance; response to alkaloid
	CC: nucleus; nucleoplasm; U12-type spliceosomal complex; nucleolus; nuclear speck
*NAA38*	MF: protein binding
	BP: negative regulation of apoptotic process
	CC: nucleus; cytoplasm; colocalizes_with polysome; NatC complex
*SKIV2L2*	MF: nucleic acid binding; RNA binding; RNA helicase activity; ATP-dependent RNA helicase activity; helicase activity
	BP: RNA catabolic process; exonucleolytic nuclear-transcribed mRNA catabolic process involved in deadenylation-dependent decay; nuclear-transcribed mRNA catabolic process, 3’-5’ exonucleolytic nonsense-mediated decay
	CC: nucleus; cytoplasm; cytosol; Ski complex
*PLRG1*	MF: protein binding
	BP: mRNA splicing, via spliceosome; mRNA processing; RNA splicing; protein localization to nucleus; positive regulation of G1/S transition of mitotic cell cycle
	CC: Prp19 complex; fibrillar center; nucleus; nucleoplasm; colocalizes_with DNA replication factor A complex

**Table 8:** Experimental grouping.

Group	Experimental Grouping
Group 1	Control 30 min: Jurkat cells were cultured for 30 min (without nsPEF) Experiment 30 min: Jurkat cells were cultured for 30 min (exposed to nsPEF)
Group 2	Control 60 min: Jurkat cells were cultured for 60 min (without nsPEF) Experiment 60 min: Jurkat cells were cultured for 60 min (exposed to nsPEF)

**Table 9:** Protein modeling.

Proteins	Species	Protein Length (aa)	Model Templates (Query Cover, Identify)
SUGP1	*Homo sapiens*	645	de novo
DHX16	*Homo sapiens*	560	5Z58_XX (94%, 99%) 5MQF_QQ (90%, 56%) 6FA9_A (89%, 55%)
FUS	*Homo sapiens*	522	de novo
HNRNPR	*Homo sapiens*	535	de novo
DHX15	*Homo sapiens*	795	5XDR_A (86%, 99%) 3KX2_B (84%, 66%) 2XAU_A (84%, 66%)
NAA38	*Homo sapiens*	125	de novo
SKIV2L2	*Homo sapiens*	1042	6D6Q_M (100%, 100%) 6C90_A (70%, 100%) 2XGJ_A (89%, 56%)
PLRG1	*Homo sapiens*	514	6FF4_D (100%, 100%) 5MQF_D (100%, 99%) 4YVD_A (72%, 100%)

## Supplementary Materials

Supplementary materials can be found at https://www.mdpi.com/1422-0067/20/23/5847/s1. Supplementary file 1: Figures; Supplementary file 2: Tables.

## Abbreviations

BP	biological process
BRD4/MYC	bromodomain-containing protein 4/MYC proto-oncogene
cBioPortal	cBio Cancer Genomics Portal
CC	cell component
DEGs	differentially expressed genes
FC	fold control
GO	gene ontology
IL7R	interleukin-7 receptor
KEGG	Kyoto Encyclopedia of Genes and Genomes pathway
MCODE	molecular complex detection
MF	molecular function
NOTCH1	notch receptor 1
nsPEF	nanosecond pulsed electric field
PPI	protein–protein interaction
PTEN	phosphatase and tensin homolog
RMSD	molecular dynamic analysis including root mean square deviation
RMSF	root mean square fluctuation
SINE	selective inhibitor of nuclear export
nsPEF	nanosecond pulsed electric field
