# Supplementary material for: Cellular Processes Involved in Jurkat Cells Exposed to Nanosecond Pulsed Electric Field
Source: Int J Mol Sci. 2019 Nov 21;20(23):5847. doi: 10.3390/ijms20235847 (PMC6929111; doi:10.3390/ijms20235847)
Supplement: Supplementary file 1 [file ijms-20-05847-s001.zip › Supplementary file 1-figures_proof1.pdf]

Supplementary Information

Cellular processes involved in Jurkat cells exposed to nanosecond pulsed electric field

Huijuan Li [1], Shibin Liu [1,*], Xue Yang [1], Yongqian Du [1], Jiezhang Luo [1], Jie Tan [1], and Yulong Sun [2,*]

1 School of Electronics and Information, Northwestern Polytechnical University, Xi'an, 710072, China; lihuijuan@mail.nwpu.edu.cn (H.J.L); liushibin@nwpu.edu.cn (S.B.L); yangx@mail.nwpu.edu.cn (X.Y); duyongqian@nwpu.edu.cn (Y.Q.D); 18829237964@163.com (J.Z.L); jietan@mail.nwpu.edu.cn (J.T)

2 Key Laboratory for Space Biosciences & Biotechnology, School of Life Sciences, Northwestern Polytechnical University, Xi'an, 710072, China; yulongsun@nwpu.edu.cn (Y.L.S)

* Correspondence: liushibin@nwpu.edu.cn (S.B.L); yulongsun@nwpu.edu.cn (Y.L.S);

Tel.: +86 29 88491653 (S.B.L); +86 29 88460332 (Y.L.S)

Address: School of Life Sciences, Northwestern Polytechnical University, 127 Youyi Xilu, Xi' an Shaan Xi Province, 710072, PR China

Tel.: +86 29 88460332, E-mail: yulongsun@nwpu.edu.cn

Figure of contents

1. **Figures**

(a)

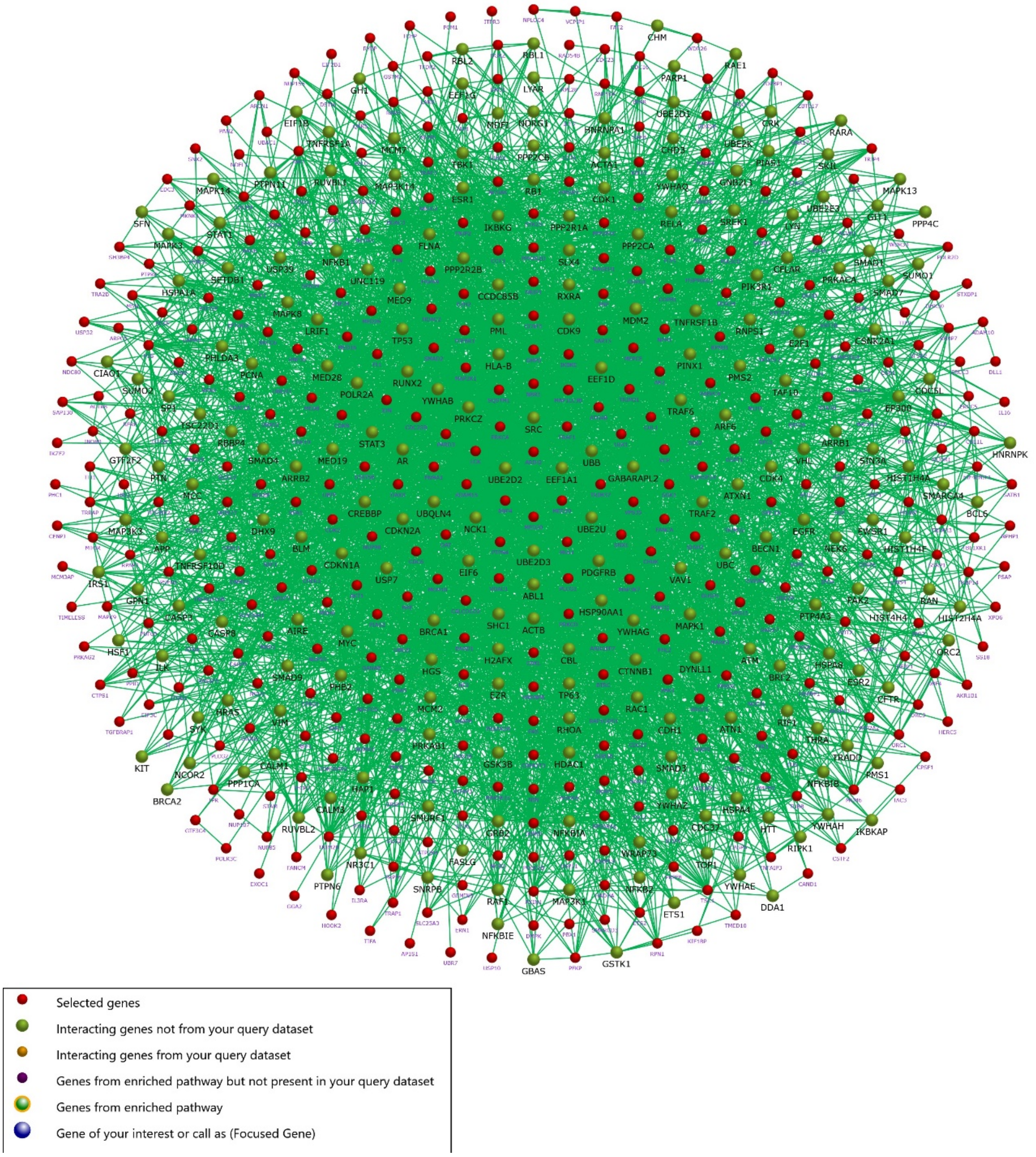
Selected genes
Interacting genes not from your query dataset
Interacting genes from your query dataset
Genes from enriched pathway but not present in your query dataset
Genes from enriched pathway
Gene of your interest or call as (Focused Gene)

(b)

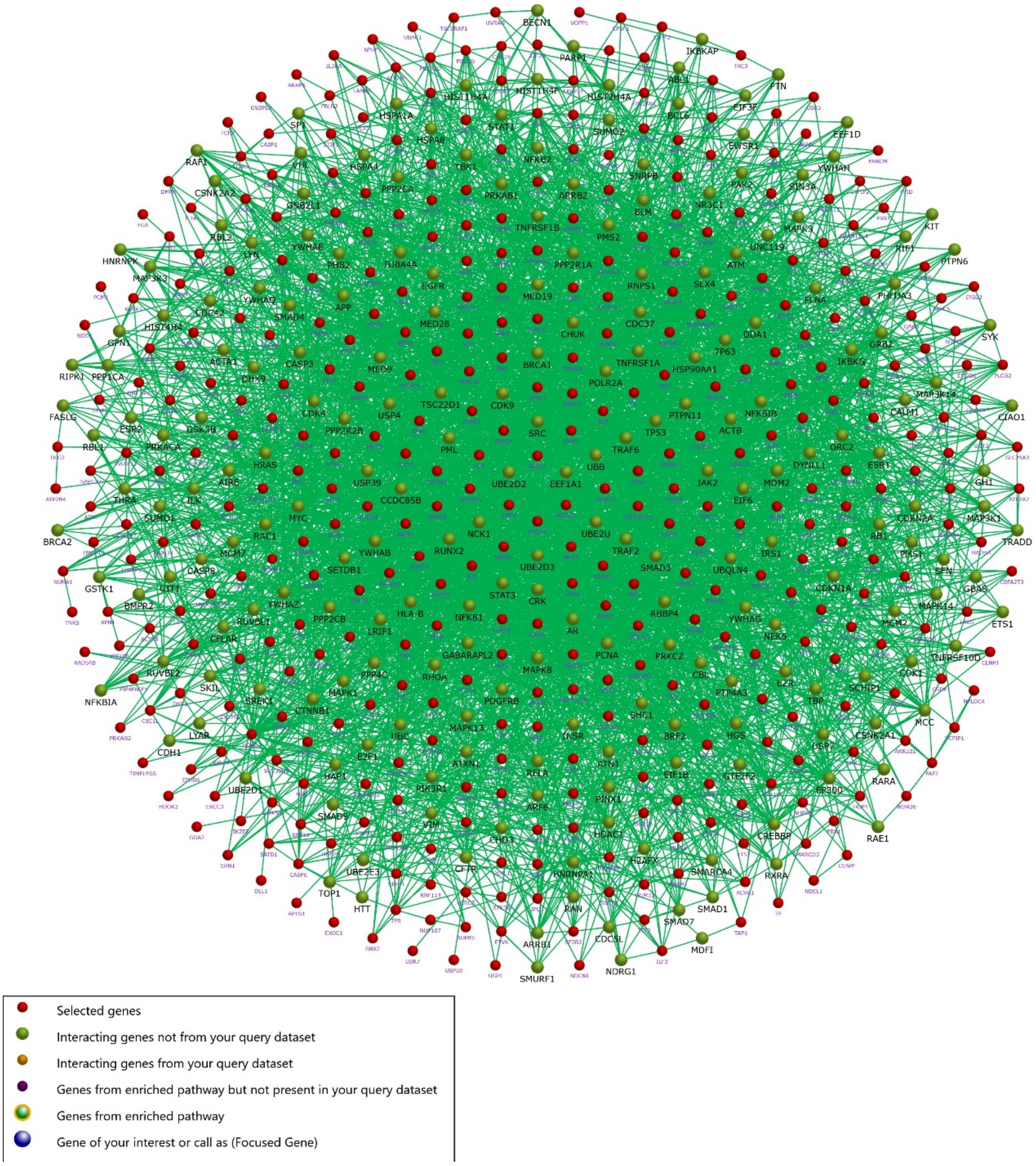

Figure S1. The interaction network analysis of differentially expressed genes for group 1 (a) and group 2 (b). Nodes less than 10 were removed because too many nodes will affect the display of data.

(a)

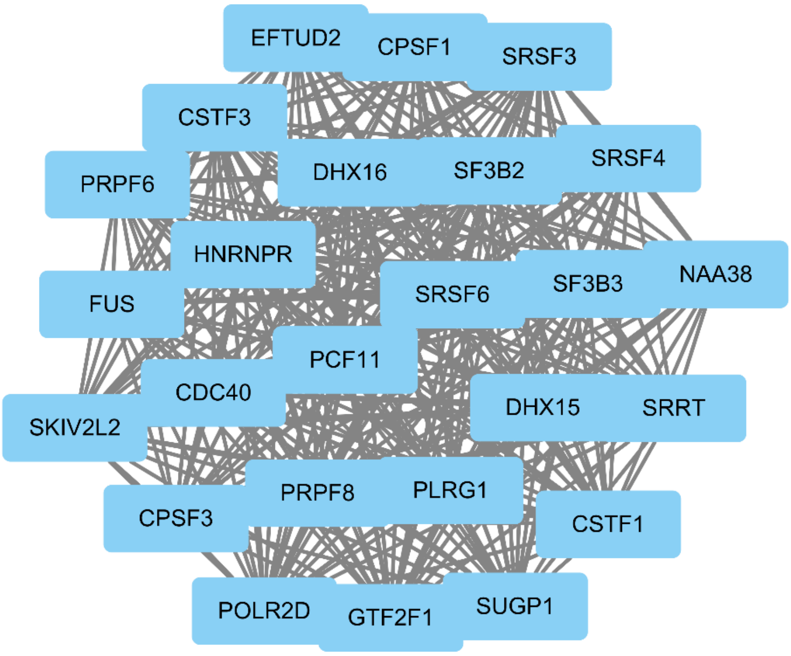

(b)

GeneSet	P-value	Correct p-value	Nodes
RNA splicing	8.73E-39	1.12E-36	*SF3B2 SF3B3 CPSF1 FUS CSTF3 CPSF3 HNRNPR PLRG1 GTF2F1 PRPF8 CDC40 EFTUD2 PRPF6 PCF11 POLR2D CSTF1 DHX15 DHX16 SRSF3 SRSF4 NAA38 SRSF6 SKIV2L2*
RNA processing	1.47E-34	9.39E-33	*SF3B2 SF3B3 CPSF1 FUS CSTF3 CPSF3 SRRT HNRNPR PLRG1 GTF2F1 PRPF8 CDC40 EFTUD2 PRPF6 PCF11 POLR2D CSTF1 DHX15 DHX16 SRSF3 SRSF4 NAA38 SRSF6 SKIV2L2*
mRNA processing	2.60E-30	1.11E-28	*SF3B2 SF3B3 CPSF1 CSTF3 CPSF3 HNRNPR PLRG1 PRPF8 CDC40 EFTUD2 PRPF6 PCF11 CSTF1 DHX15 DHX16 SRSF3 SRSF4 NAA38 SRSF6 SKIV2L2*

(c)

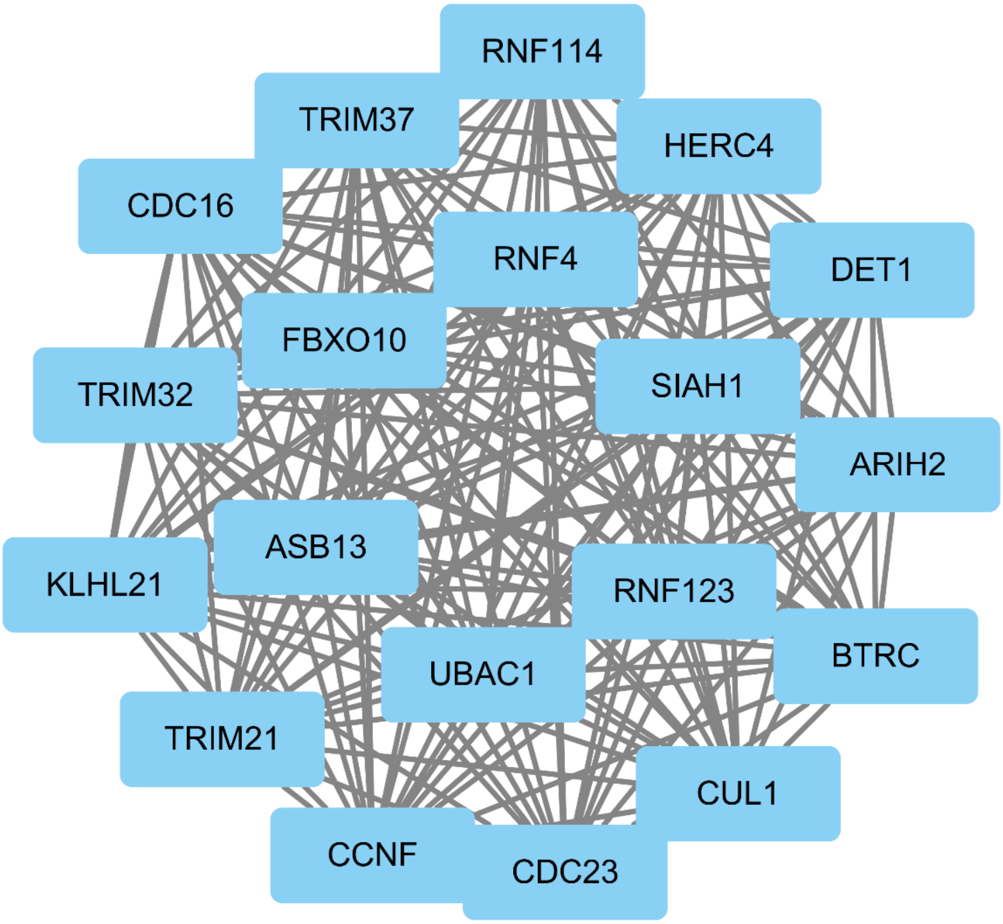

(d)

GeneSet	P-value	Correct p-value	Nodes
protein ubiquitination	5.62E-19	1.66E-16	*CDC23 CCNF SIAH1 CDC16 CUL1 KLHL21 ARIH2 RNF4 FBXO10 TRIM21 TRIM32*
protein modification by small protein conjugation	1.48E-18	2.18E-16	*CDC23 CCNF SIAH1 CDC16 CUL1 KLHL21 ARIH2 RNF4 FBXO10 TRIM21 TRIM32*
protein modification by small protein conjugation or removal	1.62E-17	1.59E-15	*CDC23 CCNF SIAH1 CDC16 CUL1 KLHL21 ARIH2 RNF4 FBXO10 TRIM21 TRIM32*

(e)

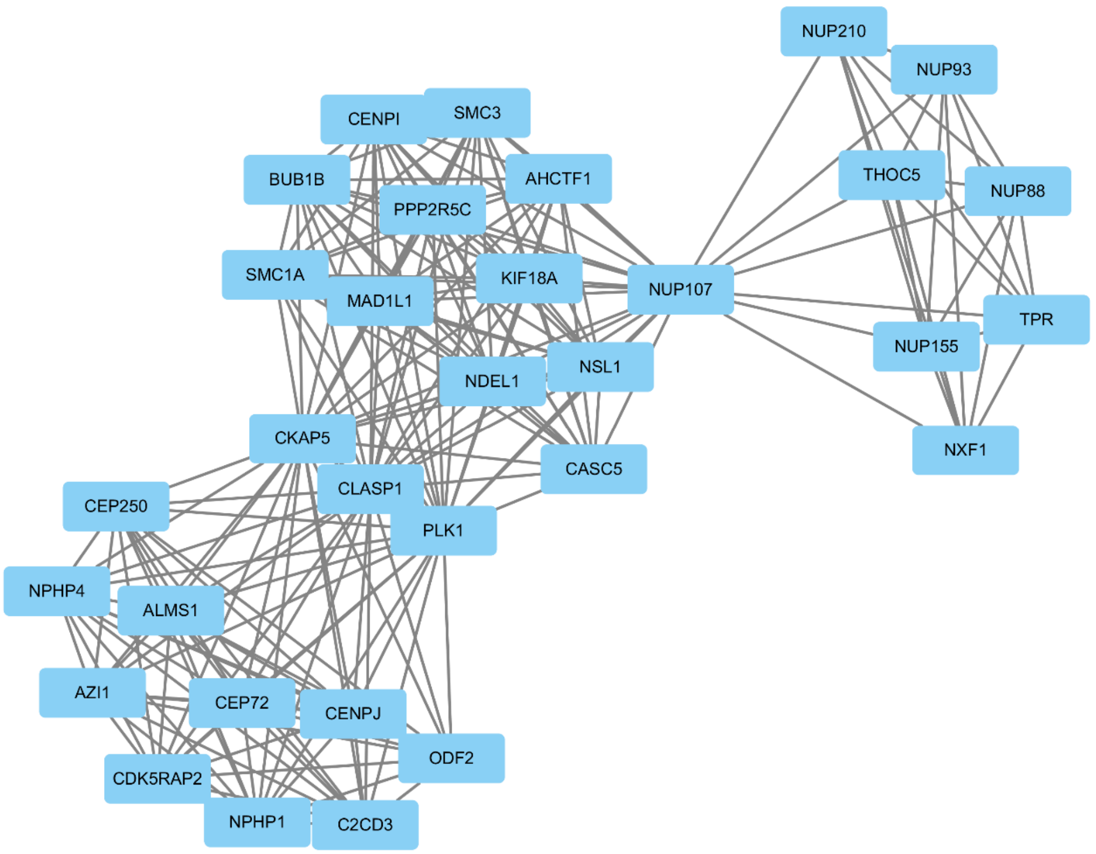

(f)

GeneSet	P-value	Correct p-value	Nodes
microtubule cytoskeleton organization	8.50E-15	1.58E-12	*KIF18A CENPJ BUB1B CASC5 CEP72 CEP250 SMC3 SMC1A CKAP5 CLASP1 NDEL1*
establishment of RNA localization	1.11E-14	1.58E-12	*NUP93 NXF1 AHCTF1 NUP107 NUP155 NUP210 TPR NUP88 THOC5 CKAP5*
nucleic acid transport	1.11E-14	1.58E-12	*NUP93 NXF1 AHCTF1 NUP107 NUP155 NUP210 TPR NUP88 THOC5 CKAP5*

(g)

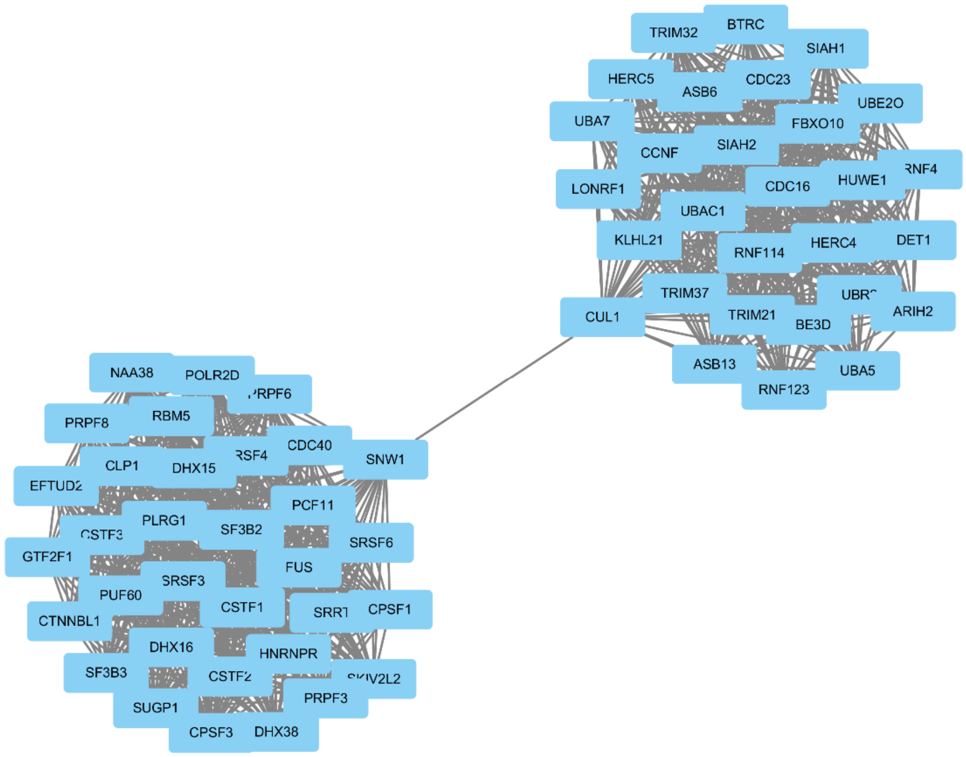

(h)

GeneSet	P-value	Correct p-value	Nodes
RNA splicing	5.04E-37	2.23E-34	*SF3B2 SF3B3 CSTF3 CSTF2 HNRNPR PRPF8 EFTUD2 PUF60 PCF11 CLP1 POLR2D DHX15 CSTF1 DHX16 DHX38 RBM5 CPSF1 FUS CPSF3 PLRG1 GTF2F1 CDC40 PRPF6 SNW1 PRPF3 SRSF3 SRSF4 NAA38 SRSF6 SKIV2L2*
mRNA processing	7.63E-31	1.69E-28	*SF3B2 SF3B3 CSTF3 CSTF2 HNRNPR PRPF8 EFTUD2 PUF60 PCF11 CLP1 DHX15 CSTF1 DHX16 DHX38 RBM5 CPSF1 CPSF3 PLRG1 CDC40 PRPF6 SNW1 PRPF3 SRSF3 SRSF4 NAA38 SRSF6 SKIV2L2*

RNA processing	1.68E-29	2.49E-27	*SF3B2 SF3B3 CSTF3 CSTF2 SRRT HNRNPR PRPF8 EFTUD2 PUF60 PCF11 CLP1 POLR2D DHX15 CSTF1 DHX16 DHX38 RBM5 CPSF1 FUS CPSF3 PLRG1 GTF2F1 CDC40 PRPF6 SNW1 PRPF3 SRSF3 SRSF4 NAA38 SRSF6 SKIV2L2*

(i)

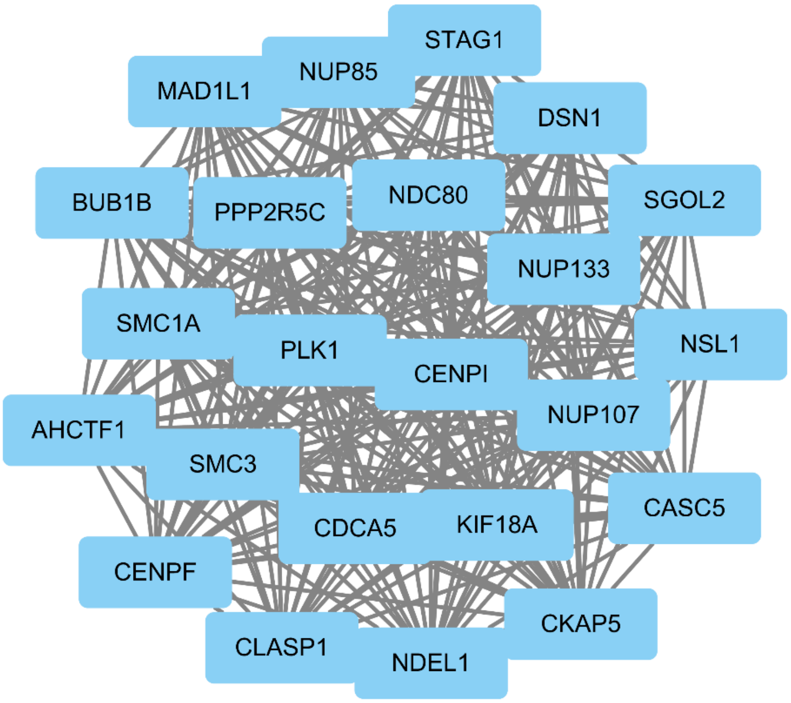

(j)

GeneSet	P-value	Correct p-value	Nodes
nuclear division	3.92E-22	6.73E-20	*CDCA5 PLK1 BUB1B CASC5 SMC1A SMC3 CKAP5 NSL1 NDC80 CENPF STAG1 DSN1 KIF18A CLASP1 MAD1L1*
mitosis	3.92E-22	6.73E-20	*CDCA5 PLK1 BUB1B CASC5 SMC1A SMC3 CKAP5 NSL1 NDC80 CENPF STAG1 DSN1 KIF18A CLASP1 MAD1L1*
cell division	4.24E-22	6.73E-20	*AHCTF1 CDCA5 PLK1 BUB1B CASC5 SMC1A SMC3 CKAP5 NSL1 NDC80 CENPF STAG1 SGOL2 DSN1 CLASP1 MAD1L1*

(k)

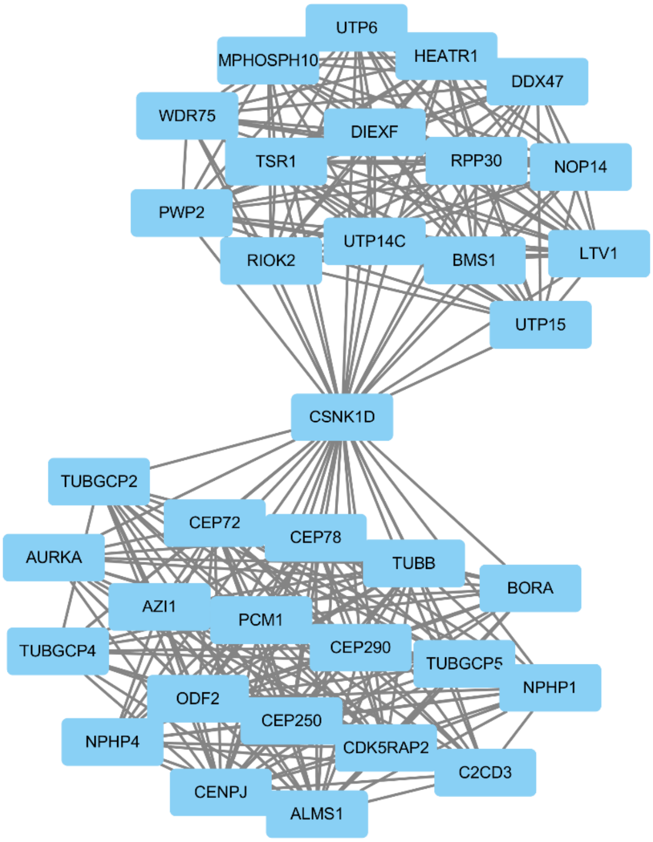

(l)

GeneSet	P-value	Correct p-value	Nodes
microtubule cytoskeleton organization	1.05E-11	4.34E-09	*PCM1 TUBGCP2 TUBB CENPJ TUBGCP5 CEP250 CEP72 TUBGCP4 AURKA*
ribosome biogenesis	1.97E-10	4.08E-08	*UTP15 NOP14 UTP6 BMS1 HEATR1 MPHOSPH10 TSR1 UTP14C*
cellular component biogenesis	3.86E-10	5.31E-08	*UTP15 NOP14 UTP6 C2CD3 TUBGCP2 TUBB HEATR1 PCM1 BMS1 CENPJ ALMS1 MPHOSPH10 TSR1 TUBGCP4 UTP14C*

Figure S2. The 3 sub-networks from the protein–protein interaction network.Group 1: (a) Module 1. (b) The enriched pathways of module 1. (c) Module 2. (d) The enriched pathways of module 2. (e) Module 3. (f) The enriched pathways of module 3. Group 2: (g) Module 1. (h) The enriched pathways of module 1. (i) Module 2. (j) The enriched pathways of module 2. (k) Module 3. (l) The enriched pathways of module 3.

(a)

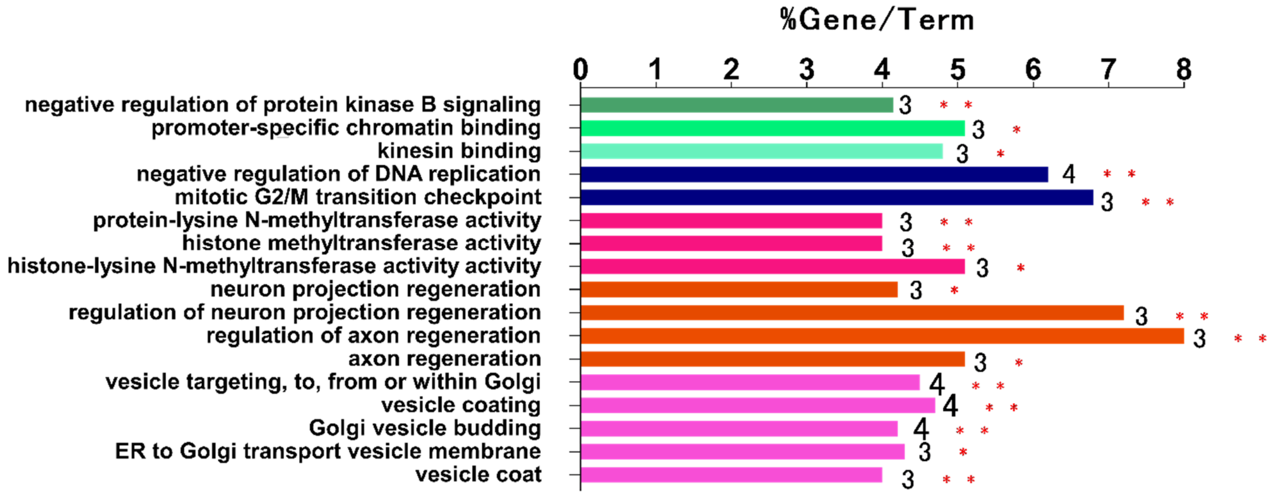
%Gene/Term
0
1
2
3
4
5
6
7
8
negative regulation of protein kinase B signaling
promoter-specific chromatin binding
kinesin binding
negative regulation of DNA replication
mitotic G2/M transition checkpoint
protein-lysine N-methyltransferase activity
histone methyltransferase activity
histone-lysine N-methyltransferase activity activity
neuron projection regeneration
regulation of neuron projection regeneration
regulation of axon regeneration
axon regeneration
vesicle targeting, to, from or within Golgi
vesicle coating
Golgi vesicle budding
ER to Golgi transport vesicle membrane
vesicle coat
3 * *
3 *
3 *
4 * *
3 * *
3 * *
3 * *
3 *
3 *
3 * *
3 * *
3 *
4 * *
4 * *
4 * *
3 *
3 * *

(b)

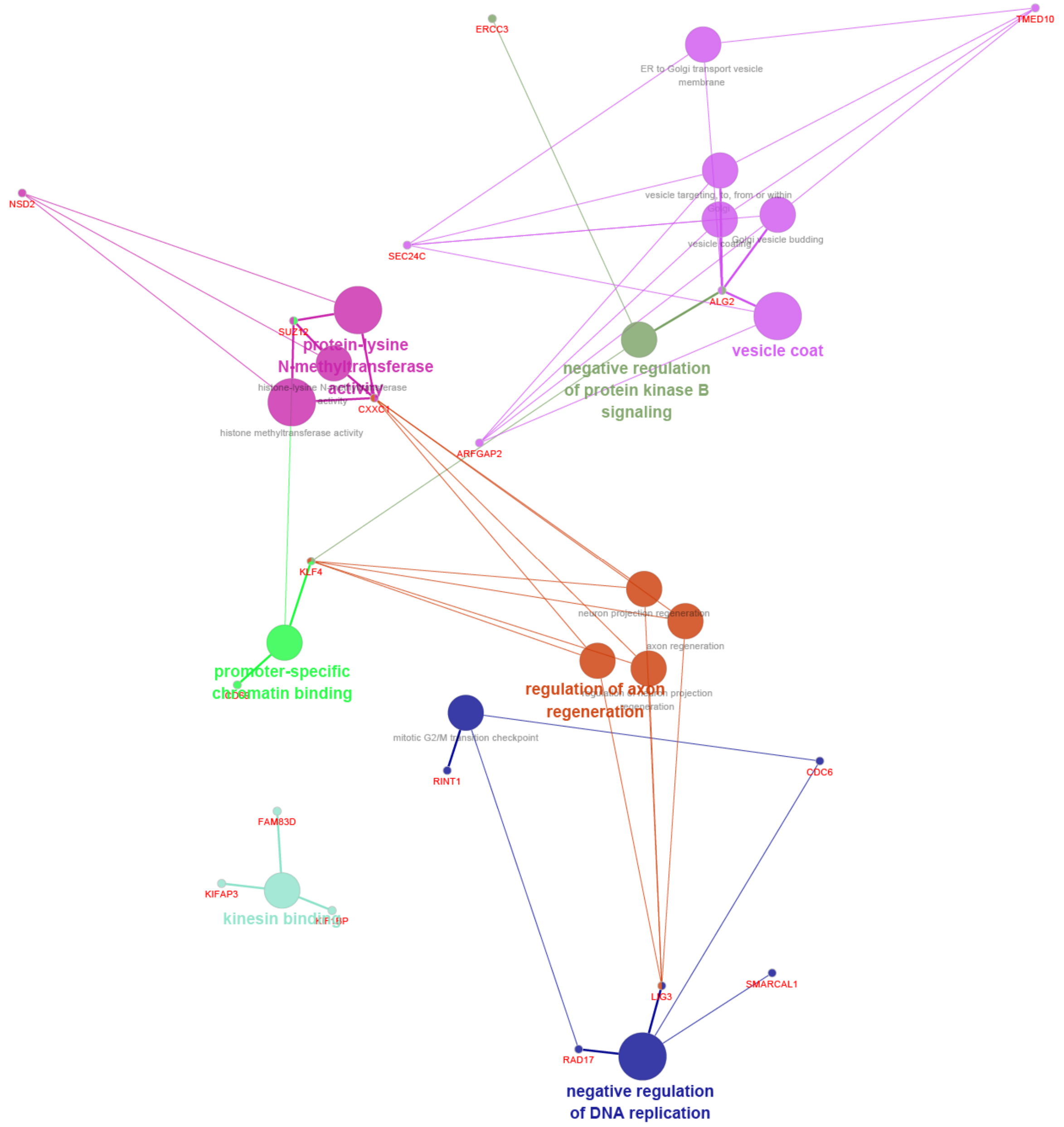

Figure S3. The top 100 genes were performed functional enrichment analysis using ClueGo tool of group 1. (a) Enriched pathways by the 100 genes. Pathways were colored based on classification (The terms were categorized in two groups shown in the two colors.). (b) Pathway-enrichment analysis using ClueGO for the top 100 genes.

(a)

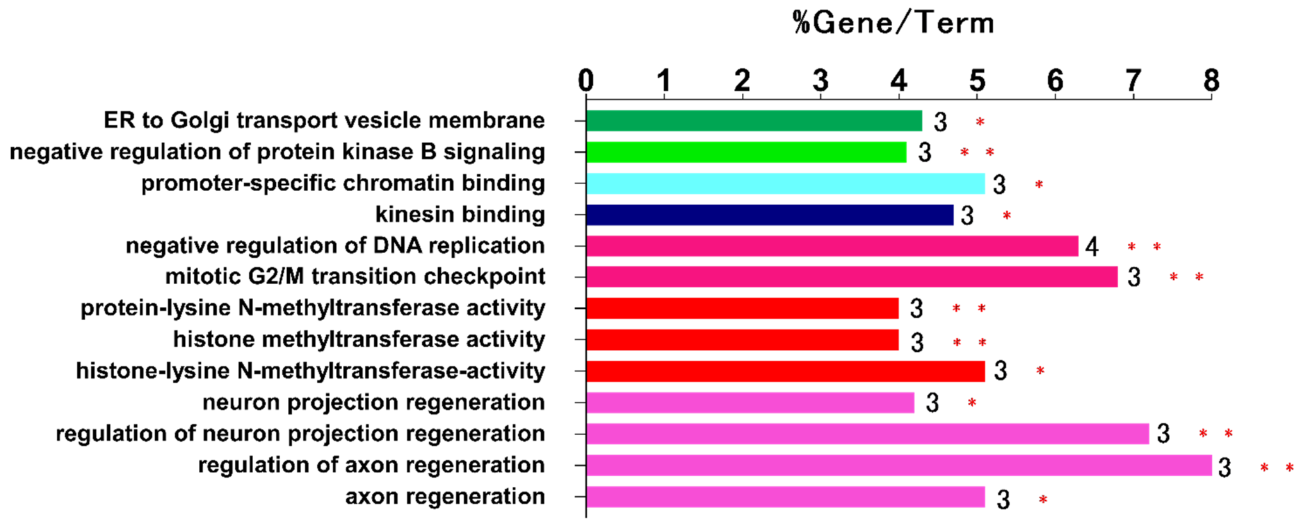

%Gene/Term
0
1
2
3
4
5
6
7
8
ER to Golgi transport vesicle membrane
negative regulation of protein kinase B signaling
promoter-specific chromatin binding
kinesin binding
negative regulation of DNA replication
mitotic G2/M transition checkpoint
protein-lysine N-methyltransferase activity
histone methyltransferase activity
histone-lysine N-methyltransferase-activity
neuron projection regeneration
regulation of neuron projection regeneration
regulation of axon regeneration
axon regeneration
3 *
3 * *
3 *
3 *
4 * *
3 * *
3 * *
3 * *
3 *
3 *
3 * *
3 * *
3 *

(b)

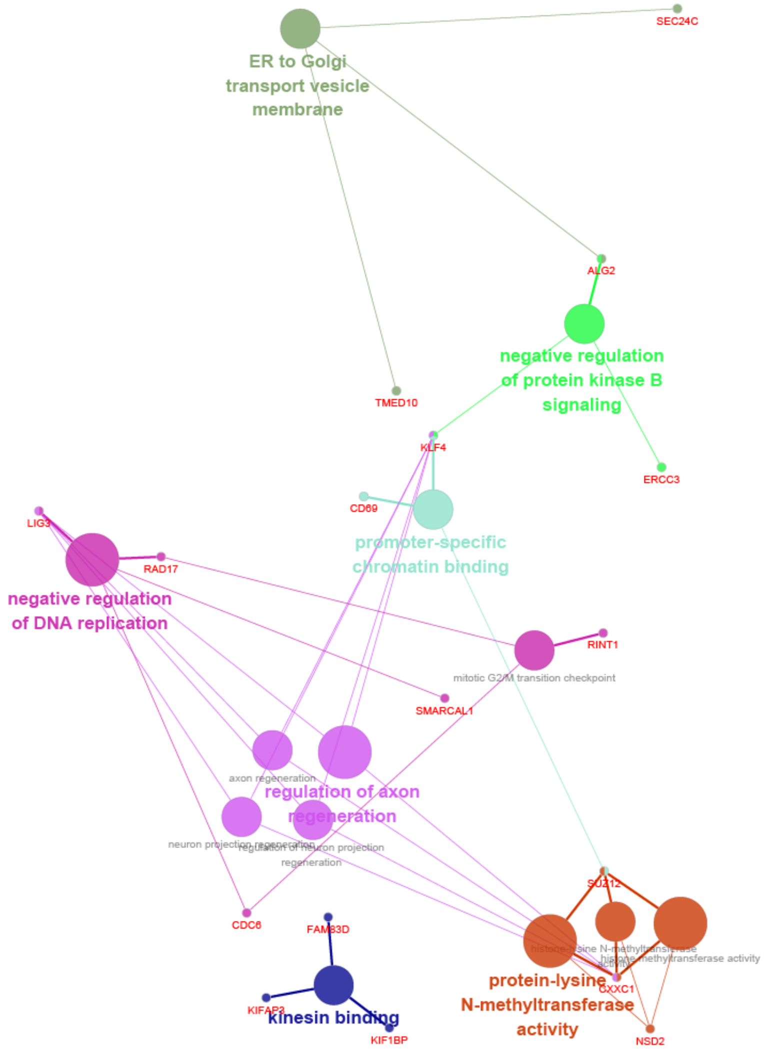

Figure S4. The top 100 genes were performed functional enrichment analysis using ClueGo tool of group 2. (a) Enriched pathways by the 100 genes. Pathways were colored based on classification (The terms were categorized in two groups shown in the two colors.). (b) Pathway-enrichment analysis using ClueGO for the top 100 genes.

(a)

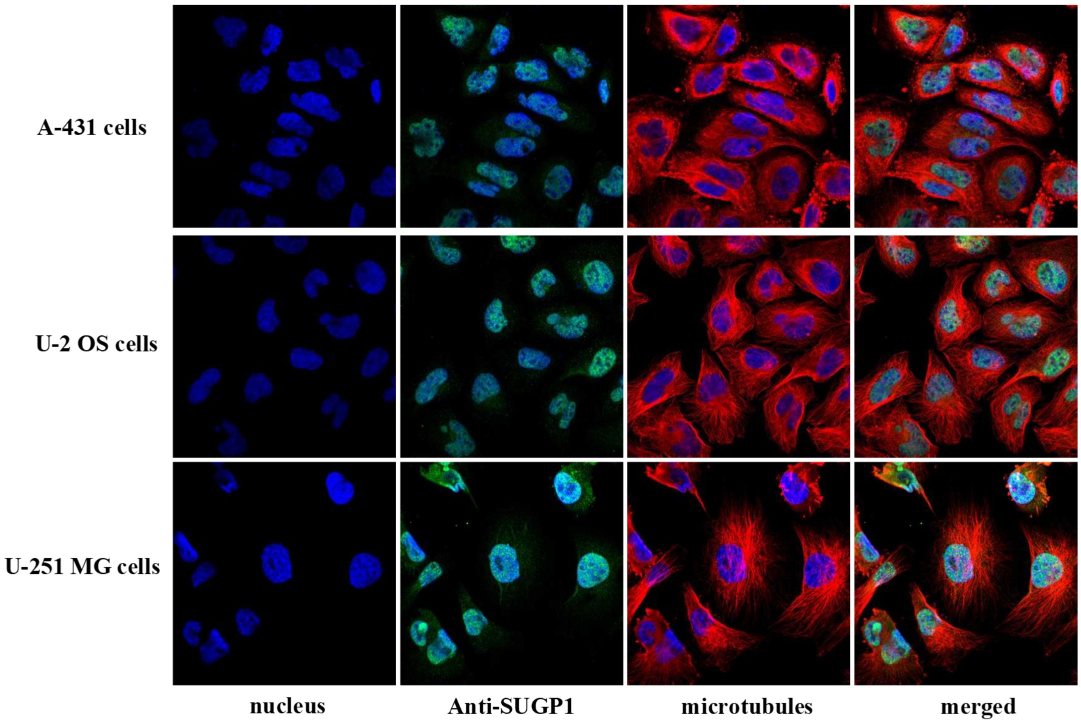

(b)

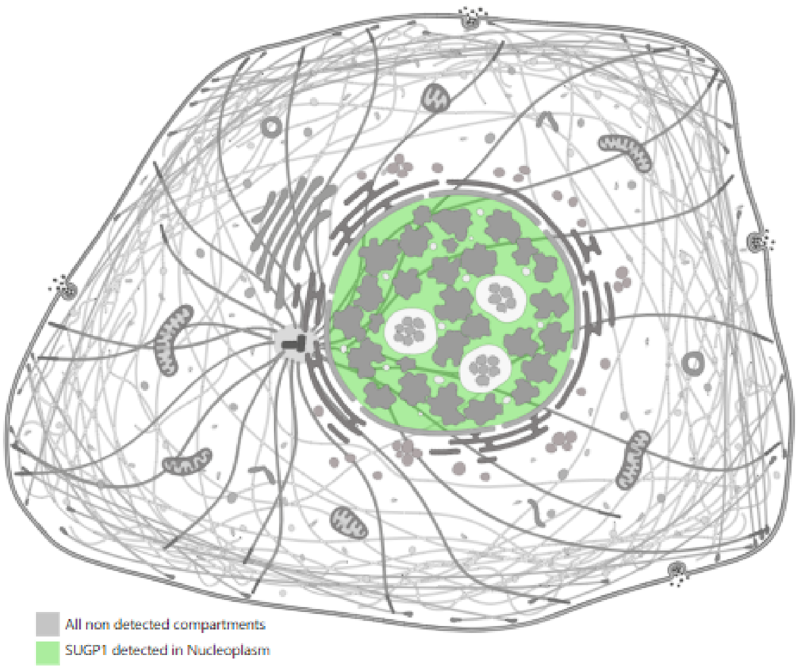

Figure S5. *SUGP1* localization in human cells (https://www.proteinatlas.org/ENSG00000146648-SUGP1/cell). (a) The localization of *SUGP1* protein in human cells. Blue: nucleus; Green: *SUGP1*; Red: microtubules. (b): Proposed schematic outline of SUGP1 in human cells (The Human Protein Atlas images are licensed under CC BY-SA 3.0 (https://creativecommons.org/licenses/by-sa/3.0)).

(a)

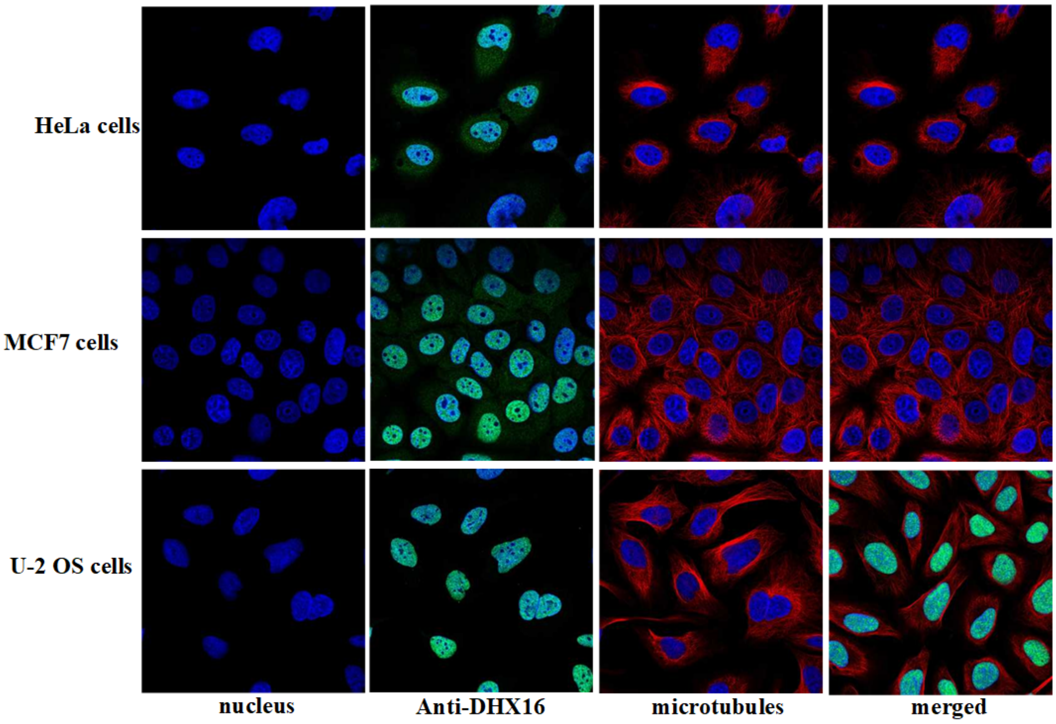

(b)

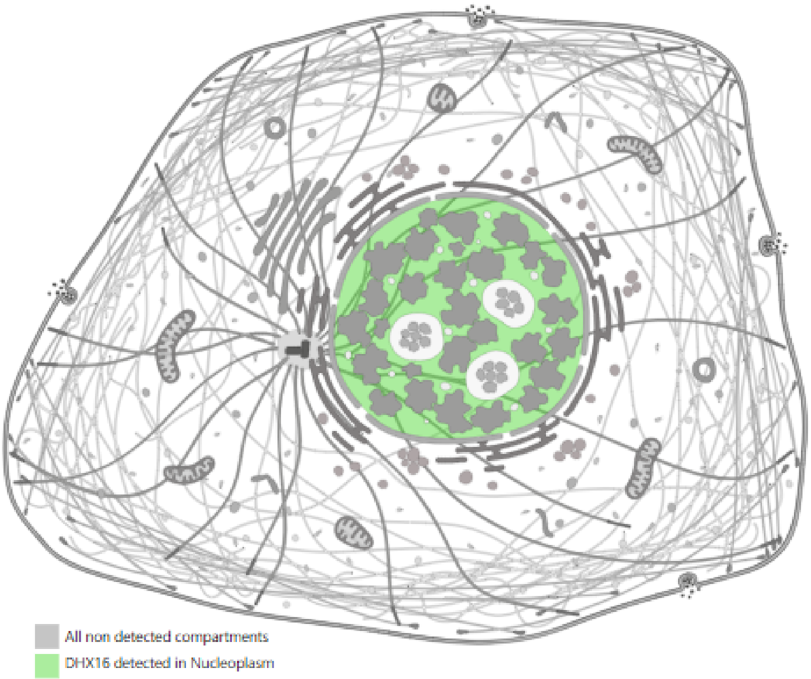

Figure S6. *DHX16* localization in human cells (https://www.proteinatlas.org/search/DHX16). (a) The localization of *DHX16* protein in human cells. Blue: nucleus; Green: *DHX16*; Red: microtubules. (b): Proposed schematic outline of *DHX16* in human cells (The Human Protein Atlas images are licensed under CC BY-SA 3.0 (https://creativecommons.org/licenses/by-sa/3.0)).

(a)

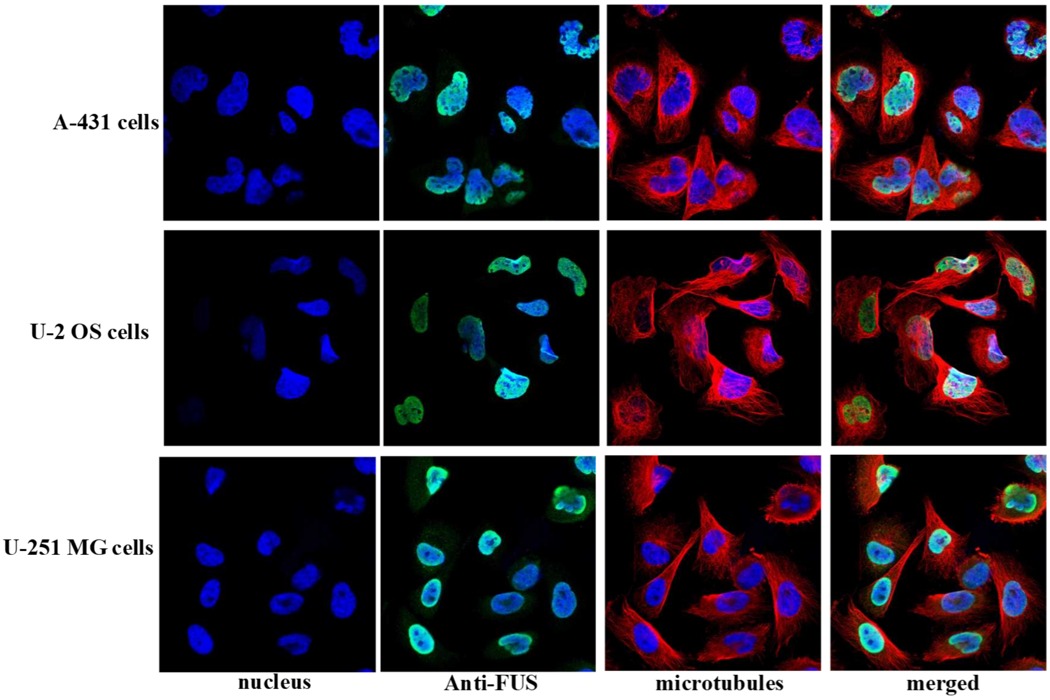

(b)

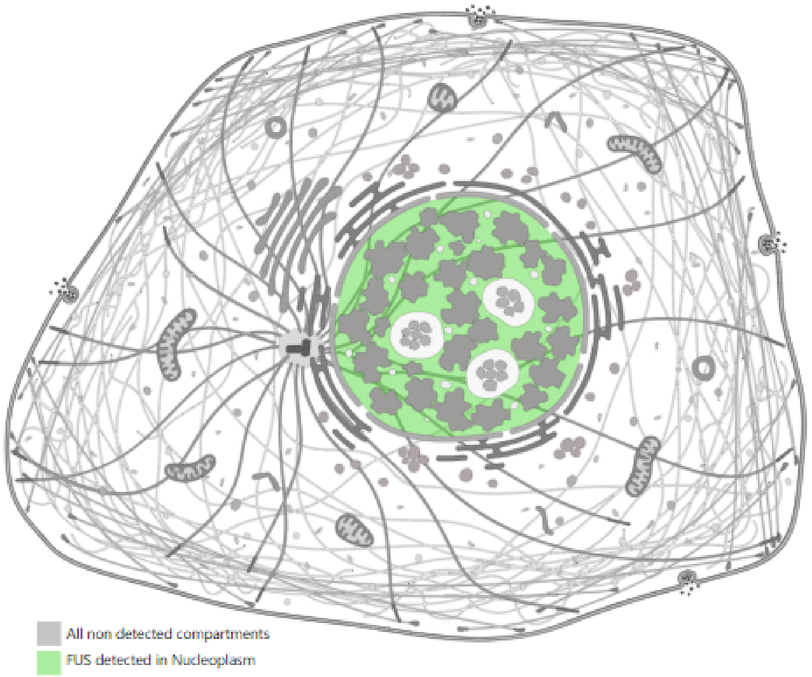

Figure S7. *FUS* localization in human cells (https://www.proteinatlas.org/search/FUS). (a) The localization of *FUS* protein in human cells. Blue: nucleus; Green: *FUS*; Red: microtubules. (b): Proposed schematic outline of *FUS* in human cells (The Human Protein Atlas images are licensed under CC BY-SA 3.0 (https://creativecommons.org/licenses/by-sa/3.0).

(a)

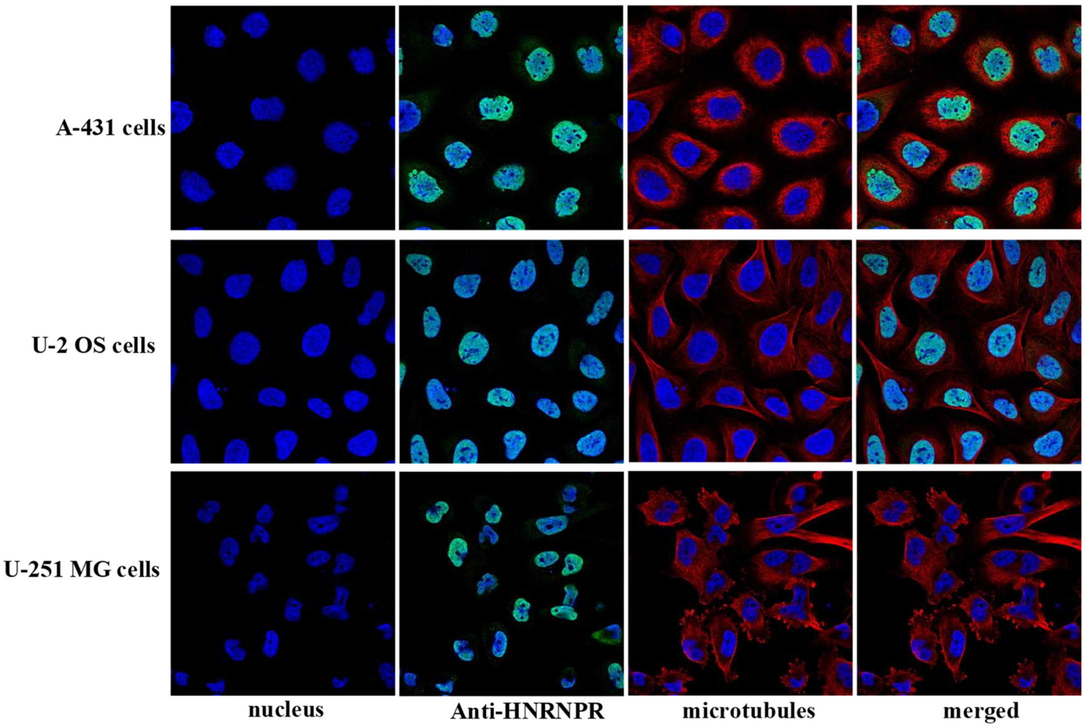

(b)

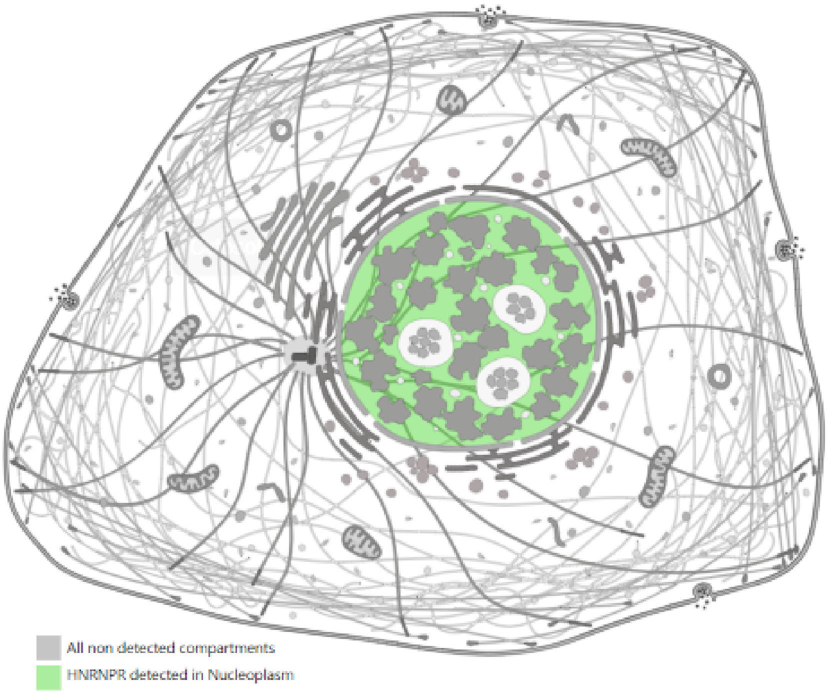

Figure S8. *HNRNPR* localization in human cells (https://www.proteinatlas.org/search/HNRNPR). (a) The localization of *HNRNPR* protein in human cells. Blue: nucleus; Green: *HNRNPR*; Red: microtubules. (b): Proposed schematic outline of *HNRNPR* in human cells (The Human Protein Atlas images are licensed under CC BY-SA 3.0 (https://creativecommons.org/licenses/by-sa/3.0).

(a)

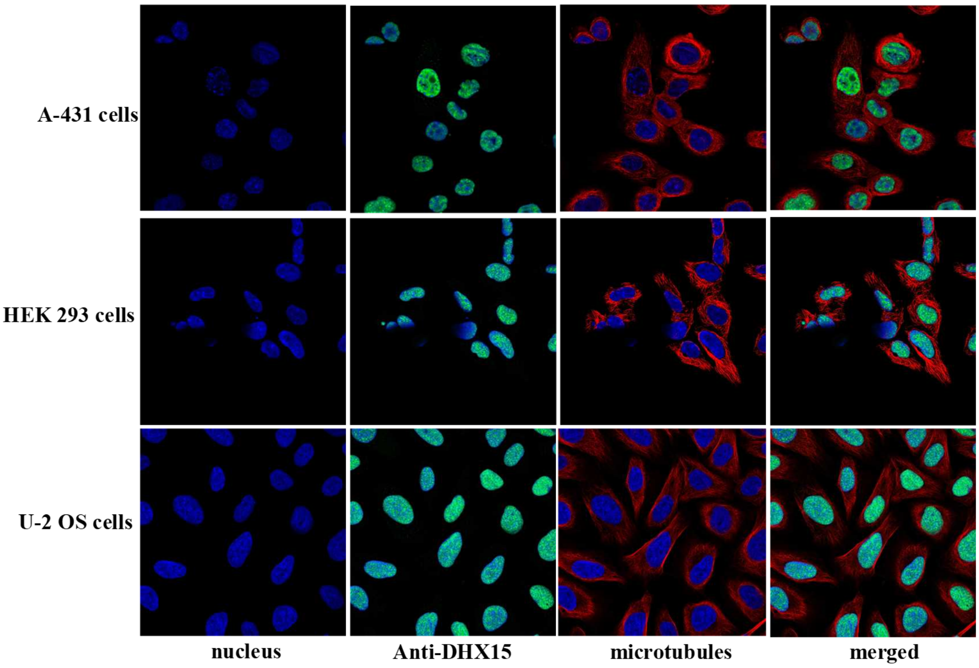

(b)

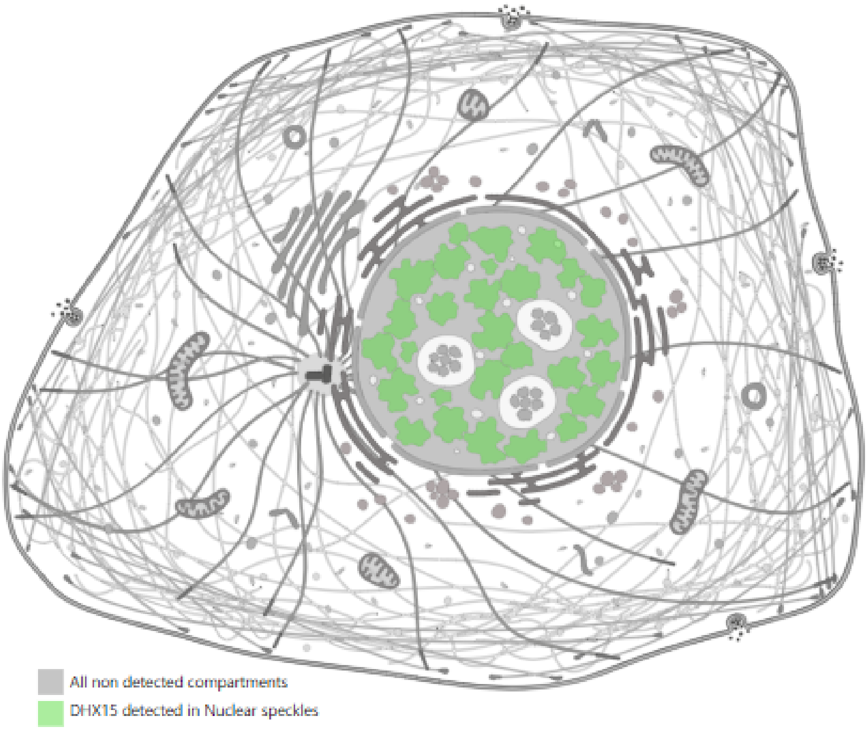

Figure S9. *DHX15* localization in human cells (https://www.proteinatlas.org/search/DHX15). (a) The localization of *DHX15* protein in human cells. Blue: nucleus; Green: *DHX15*; Red: microtubules. (b): Proposed schematic outline of *DHX15* in human cells (The Human Protein Atlas images are licensed under CC BY-SA 3.0 (https://creativecommons.org/licenses/by-sa/3.0).

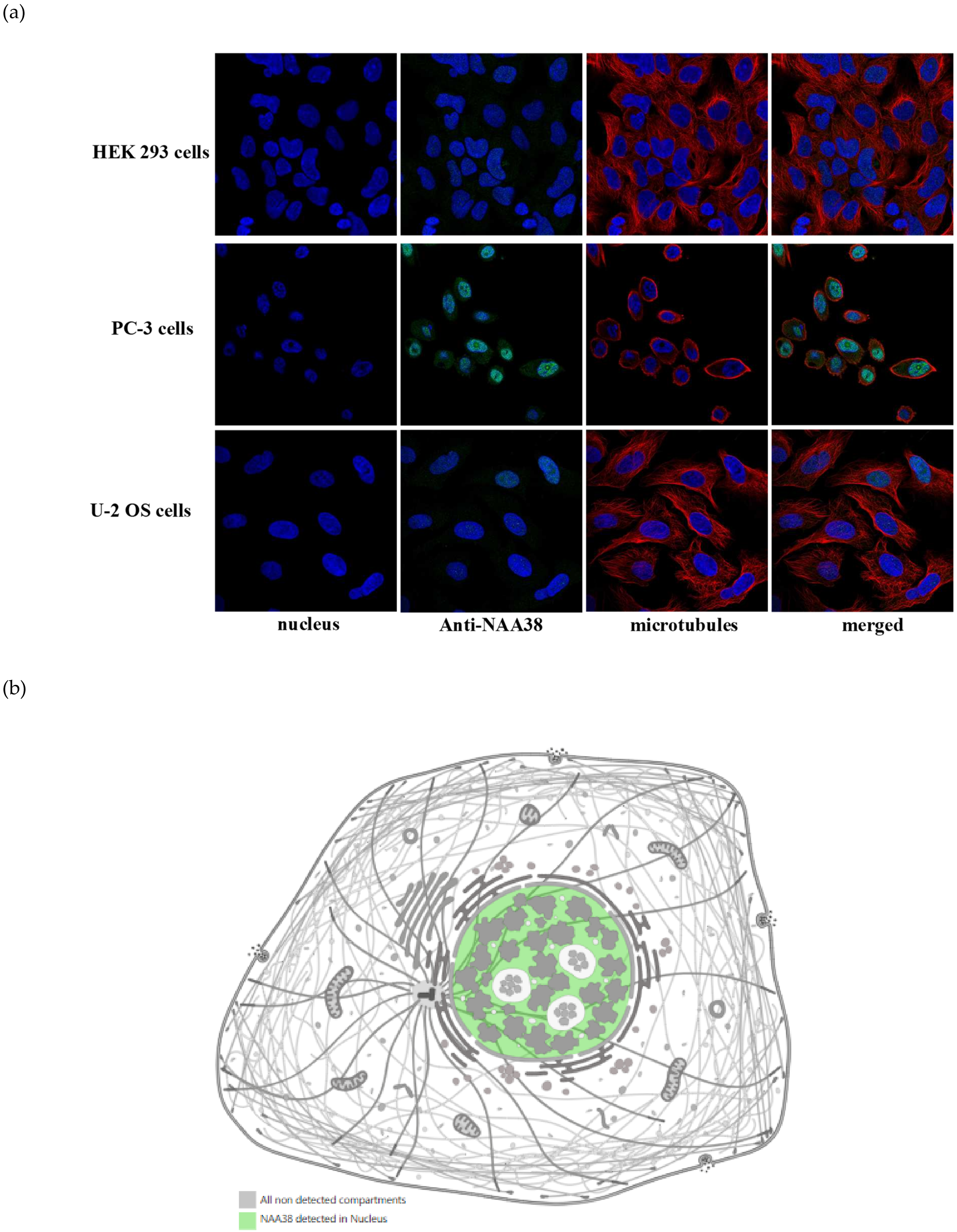

Figure S10. *NAA38* localization in human cells (https://www.proteinatlas.org/search/NAA38). (a) The localization of *NAA38* protein in human cells. Blue: nucleus; Green: *NAA38*; Red: microtubules. (b): Proposed schematic outline of *NAA38* in human cells (The Human Protein Atlas images are licensed under CC BY-SA 3.0 (https://creativecommons.org/licenses/by-sa/3.0).

(a)

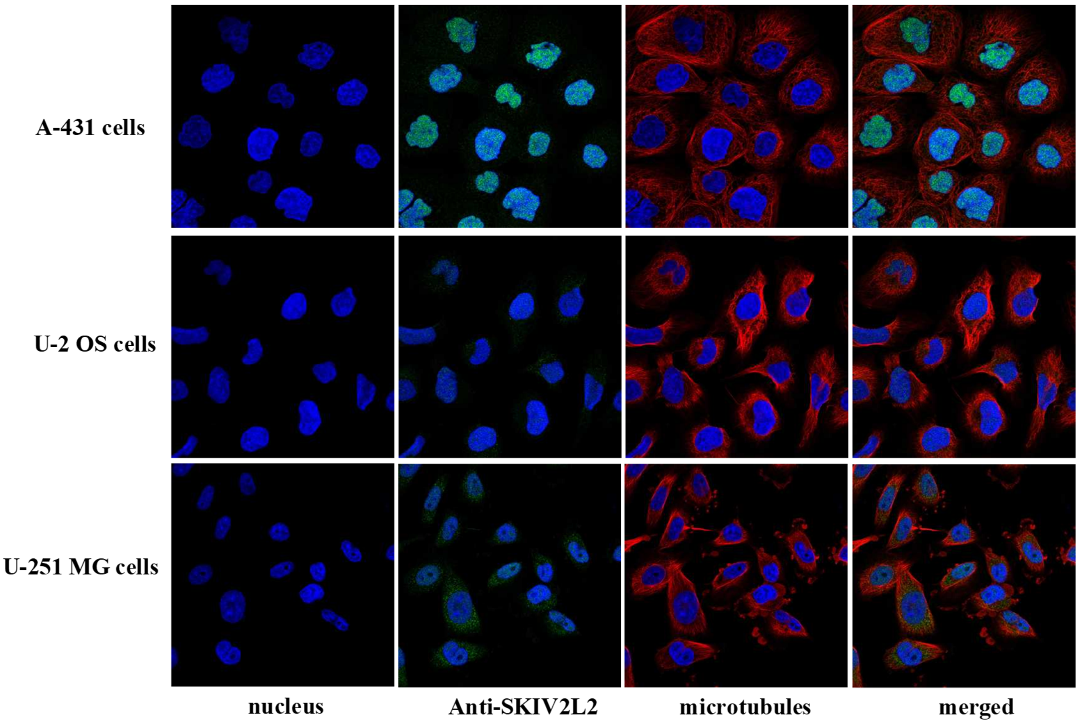

(b)

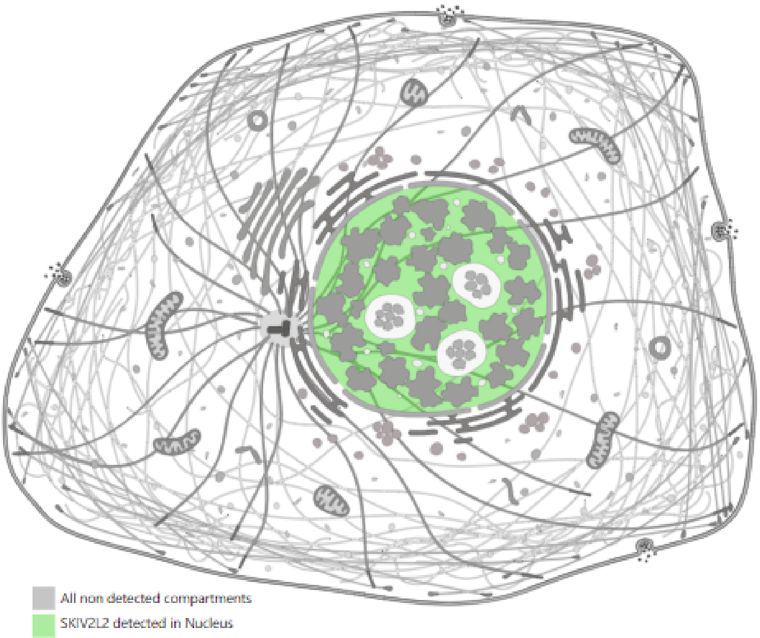

Figure S11. *SKIV2L2* localization in human cells (https://www.proteinatlas.org/search/SKIV2L2). (a) The localization of *SKIV2L2* protein in human cells. Blue: nucleus; Green: *SKIV2L2*; Red: microtubules. (b): Proposed schematic outline of *SKIV2L2* in human cells (The Human Protein Atlas images are licensed under CC BY-SA 3.0 (https://creativecommons.org/licenses/by-sa/3.0).

(a)

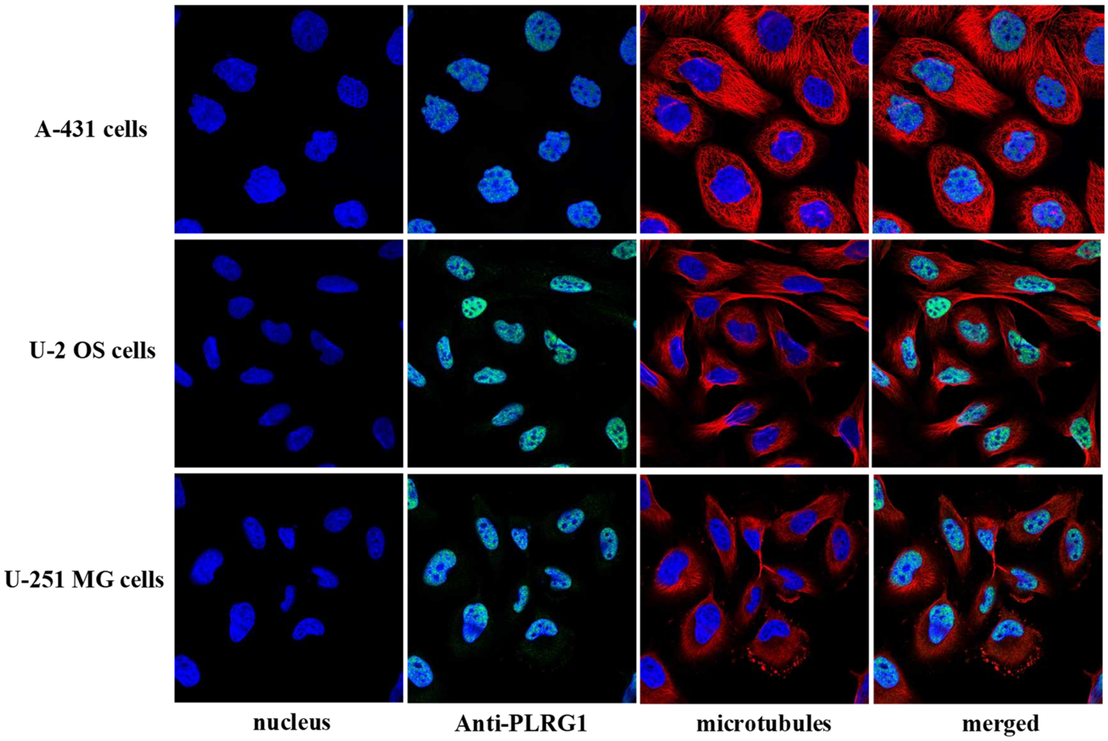

(b)

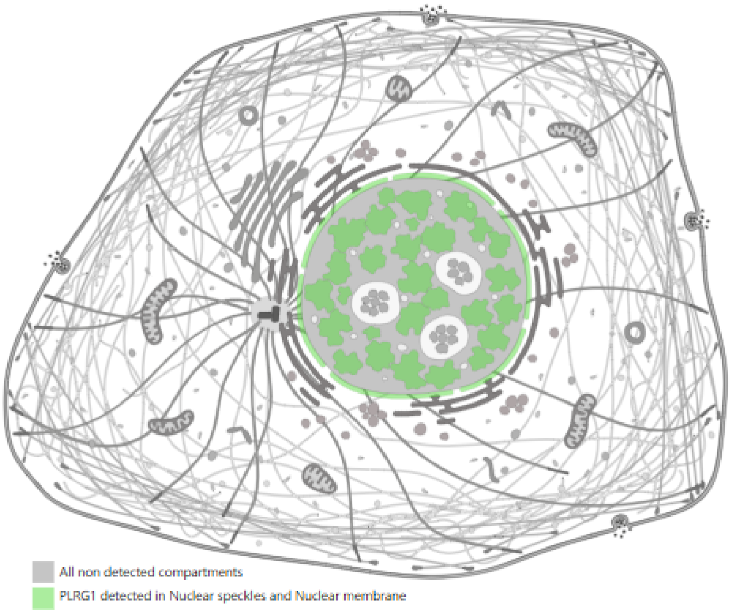

Figure S12. *PLRG1* localization in human cells (https://www.proteinatlas.org/search/PLRG1). (a) The localization of *PLRG1* protein in human cells. Blue: nucleus; Green: *PLRG1*; Red: microtubules. (b): Proposed schematic outline of *PLRG1* in human cells (The Human Protein Atlas images are licensed under CC BY-SA 3.0 (https://creativecommons.org/licenses/by-sa/3.0).

(a)

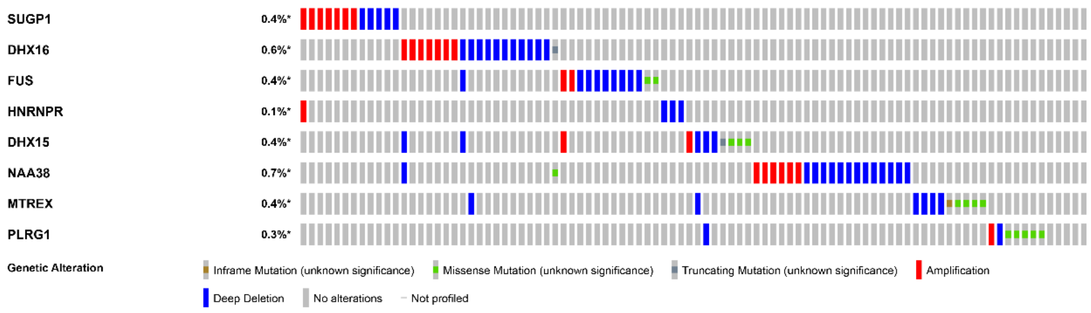
SUGP1
0.4%*
DHX16
0.6%*
FUS
0.4%*
HNRNPR
0.1%*
DHX15
0.4%*
NAA38
0.7%*
MTREX
0.4%*
PLRG1
0.3%*
Genetic Alteration
Inframe Mutation (unknown significance)
Missense Mutation (unknown significance)
Truncating Mutation (unknown significance)
Amplification
Deep Deletion
No alterations
Not profiled

(b)

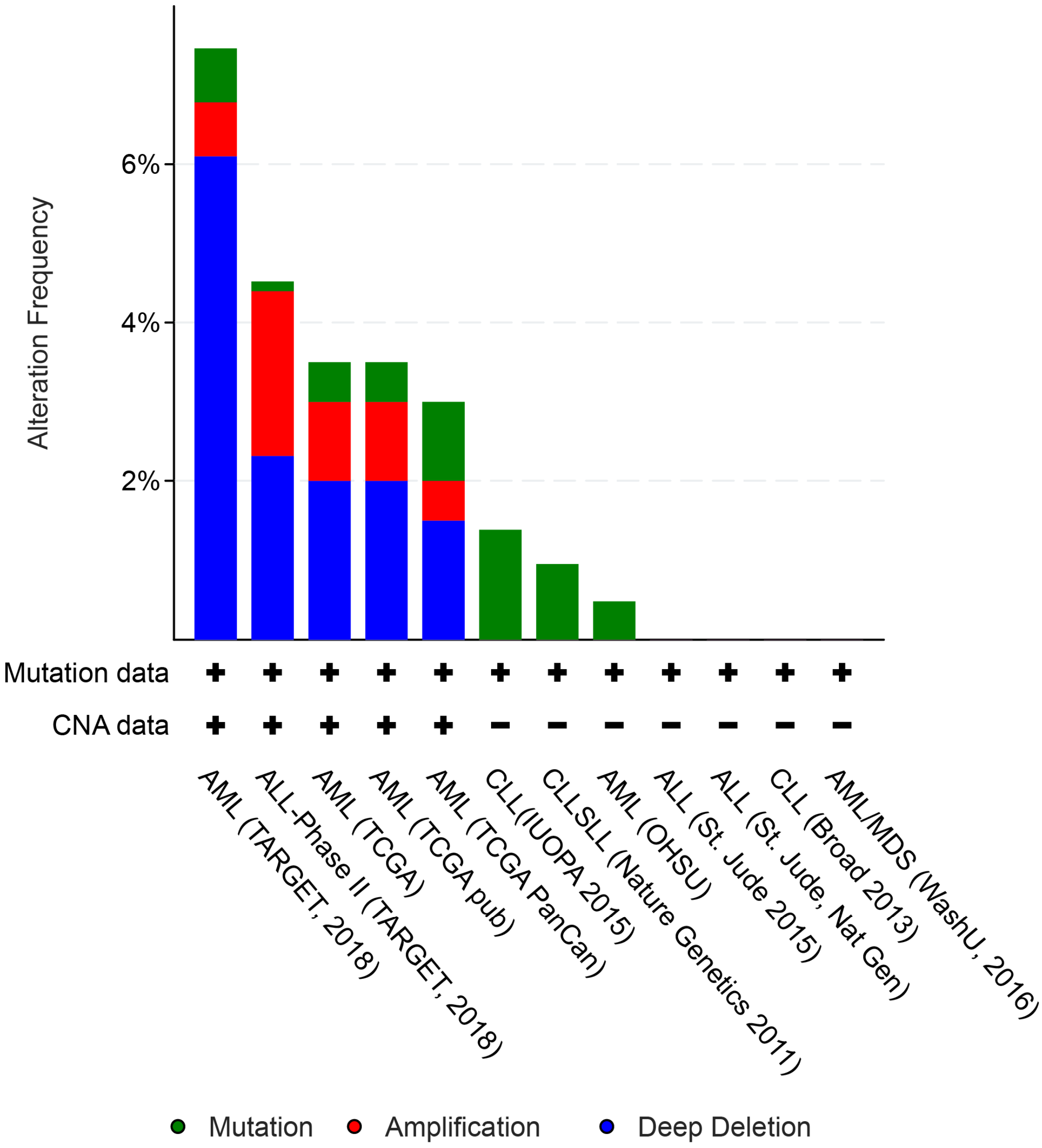
Alteration Frequency
6%
4%
2%
Mutation data
CNA data
AML (TARGET, 2018)
ALL-Phase II (TARGET, 2018)
AML (TCGA)
AML (TCGA pub)
AML (TCGA PanCan)
CLL(IUOPA 2015)
CLLSLL (Nature Genetics 2011)
AML (OHSU)
ALL (St. Jude 2015)
ALL (St. Jude, Nat Gen)
CLL (Broad 2013)
AML/MDS (WashU, 2016)
Mutation
Amplification
Deep Deletion

(c)

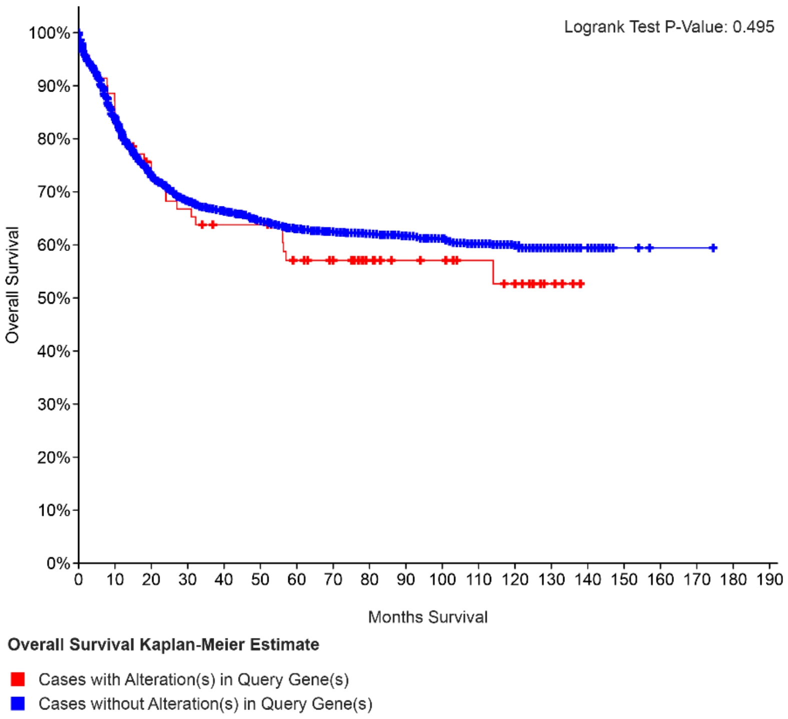
Logrank Test P-Value: 0.495
100%
90%
80%
70%
60%
50%
40%
30%
20%
10%
0%
Overall Survival
0 10 20 30 40 50 60 70 80 90 100 110 120 130 140 150 160 170 180 190
Months Survival
Overall Survival Kaplan-Meier Estimate
Cases with Alteration(s) in Query Gene(s)
Cases without Alteration(s) in Query Gene(s)

(d)

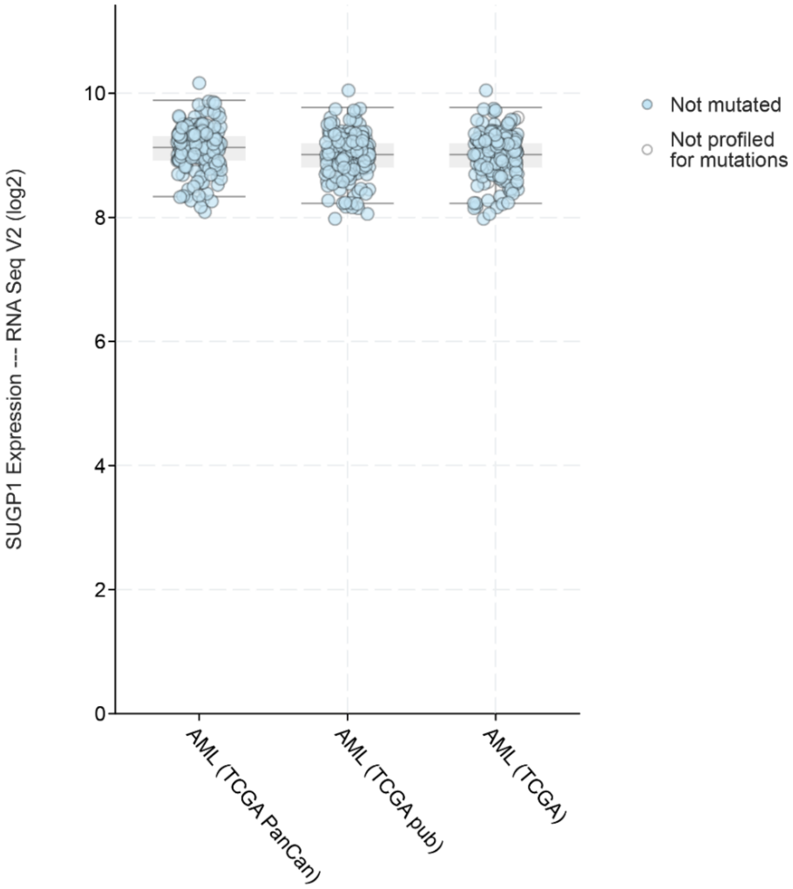
SUGP1 Expression --- RNA Seq V2 (log2)
10
8
6
4
2
0
AML (TCGA PanCan)
AML (TCGA pub)
AML (TCGA)
Not mutated
Not profiled for mutations

(e)

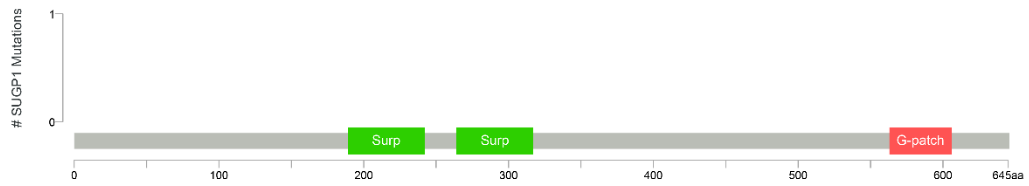

Figure S13. Genetic alterations of the hub genes were analyzed using the cBioPortal. (a) Genetic alterations of the hub genes were analyzed using cBioPortal. Grey bars along a vertical line represent the same sample interrogated for amplification (red), deep deletion (blue), missense mutation (green), truncating mutation (black) or in-frame mutation (brown). (b) The alteration frequency of an 8-gene signature (*SUGP1, DHX16, FUS, HNRNPR, DHX15, NAA38, SKIV2L2* and *PLRG1*) was determined using the cBioPortal (http://www. cbioportal.org). (c) Overall Survival Kaplan–Meier estimate. (d) Relative expression level as a function of relative copy number of *SUGP1* gene was plotted in different database. (e) The distribution of *SUGP1* mutations in leukemia across protein domains.

(a)

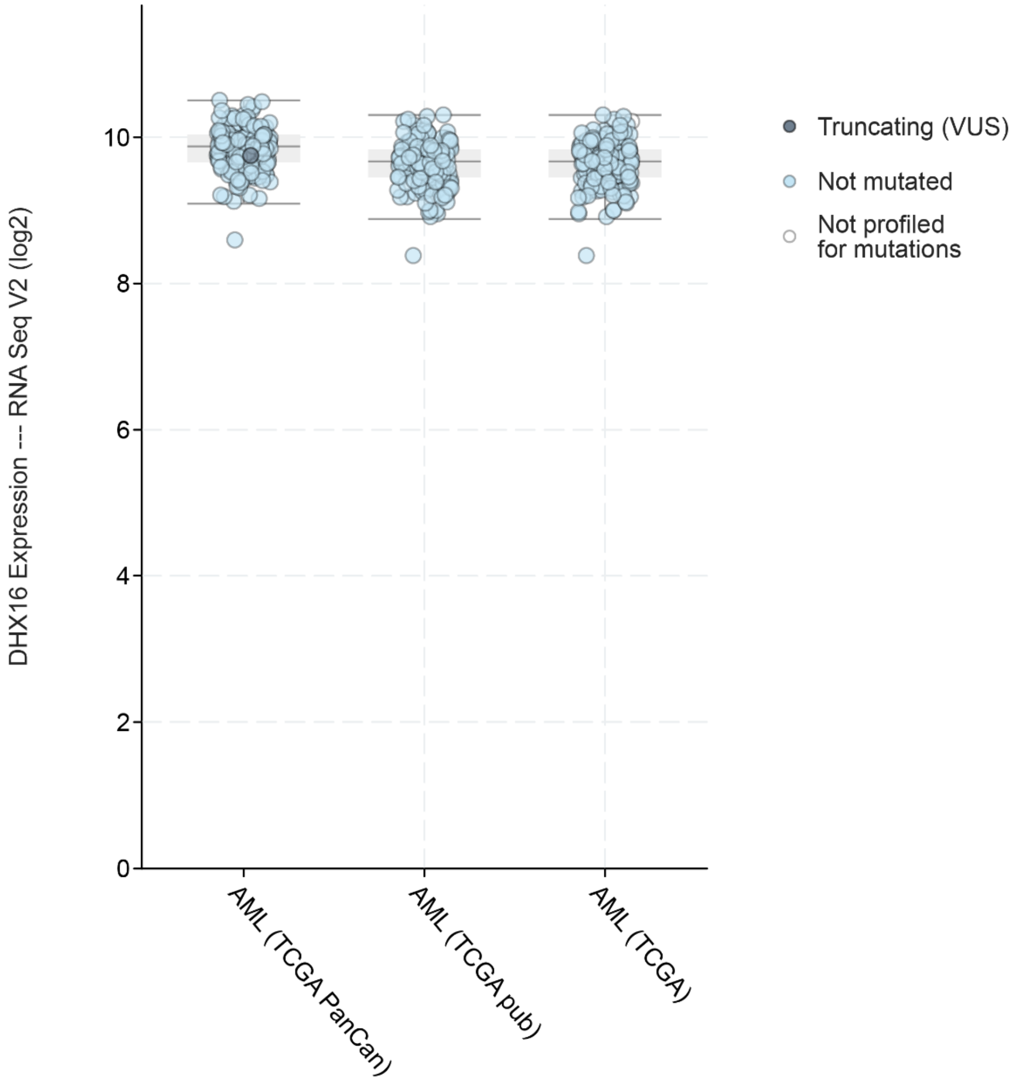

(b)

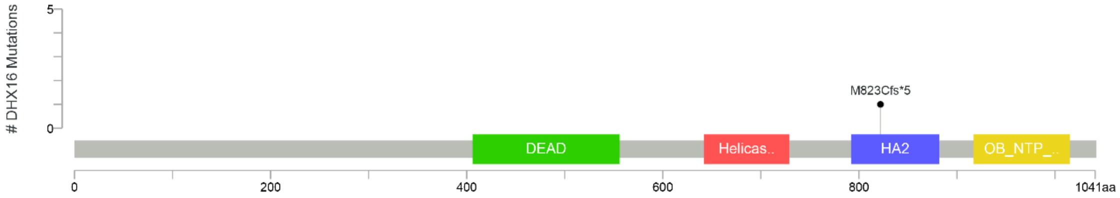

Figure S14. The alterations of *DHX16* gene in cBioportal Web resource online. (a) Relative expression level as a function of relative copy number of *DHX16* gene was plotted in different database. (b) The distribution of *DHX16* mutations in leukemia across protein domains.

(a)

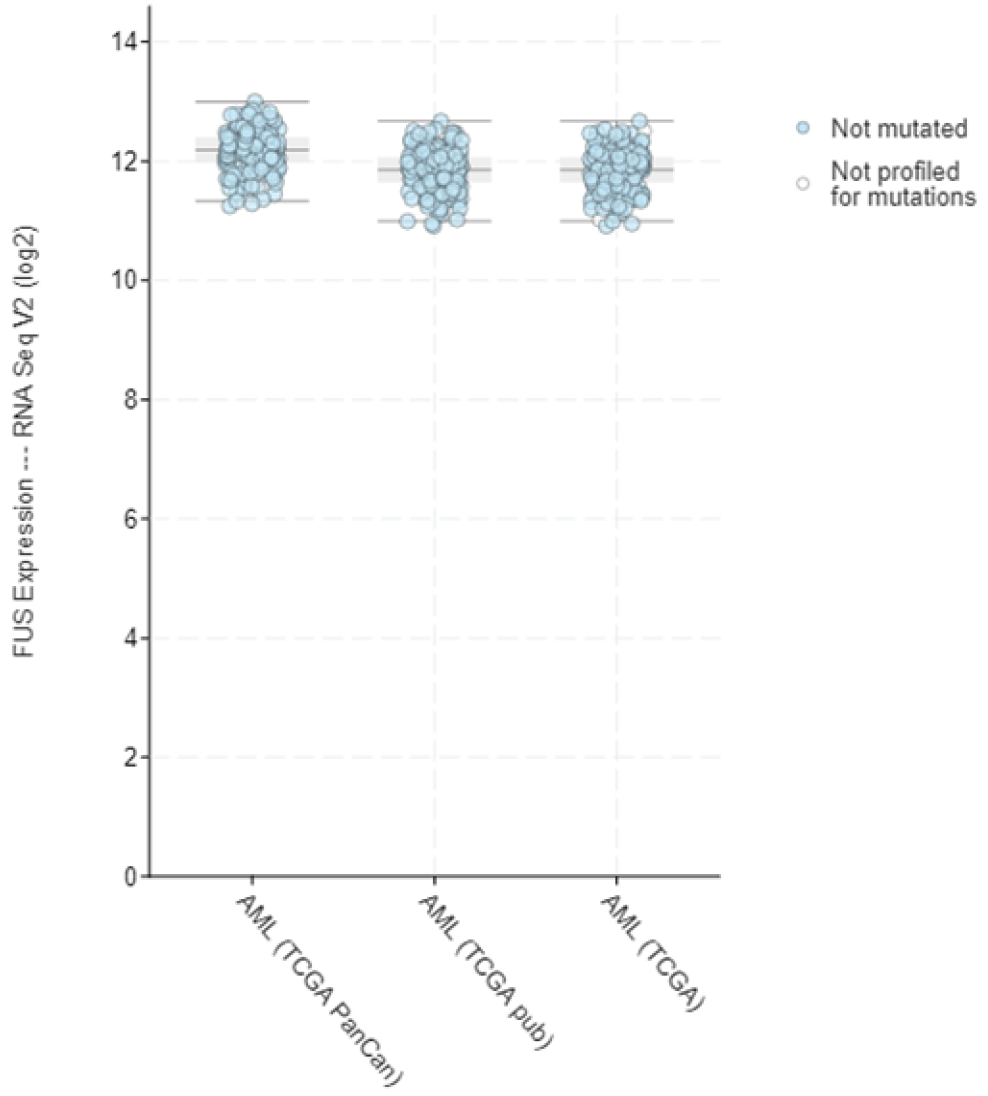

(b)

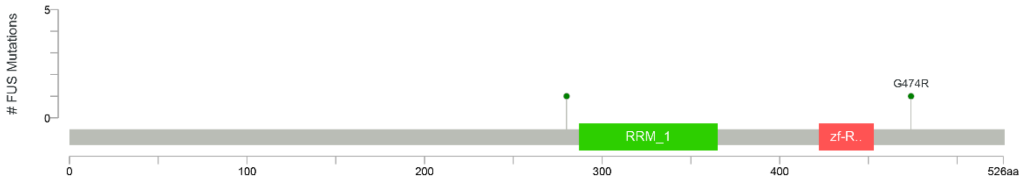

Figure S15. The alterations of *FUS* gene in cBioportal Web resource online. (a) Relative expression level as a function of relative copy number of *FUS* gene was plotted in different database. (b) The distribution of *FUS* mutations in leukemia across protein domains.

(a)

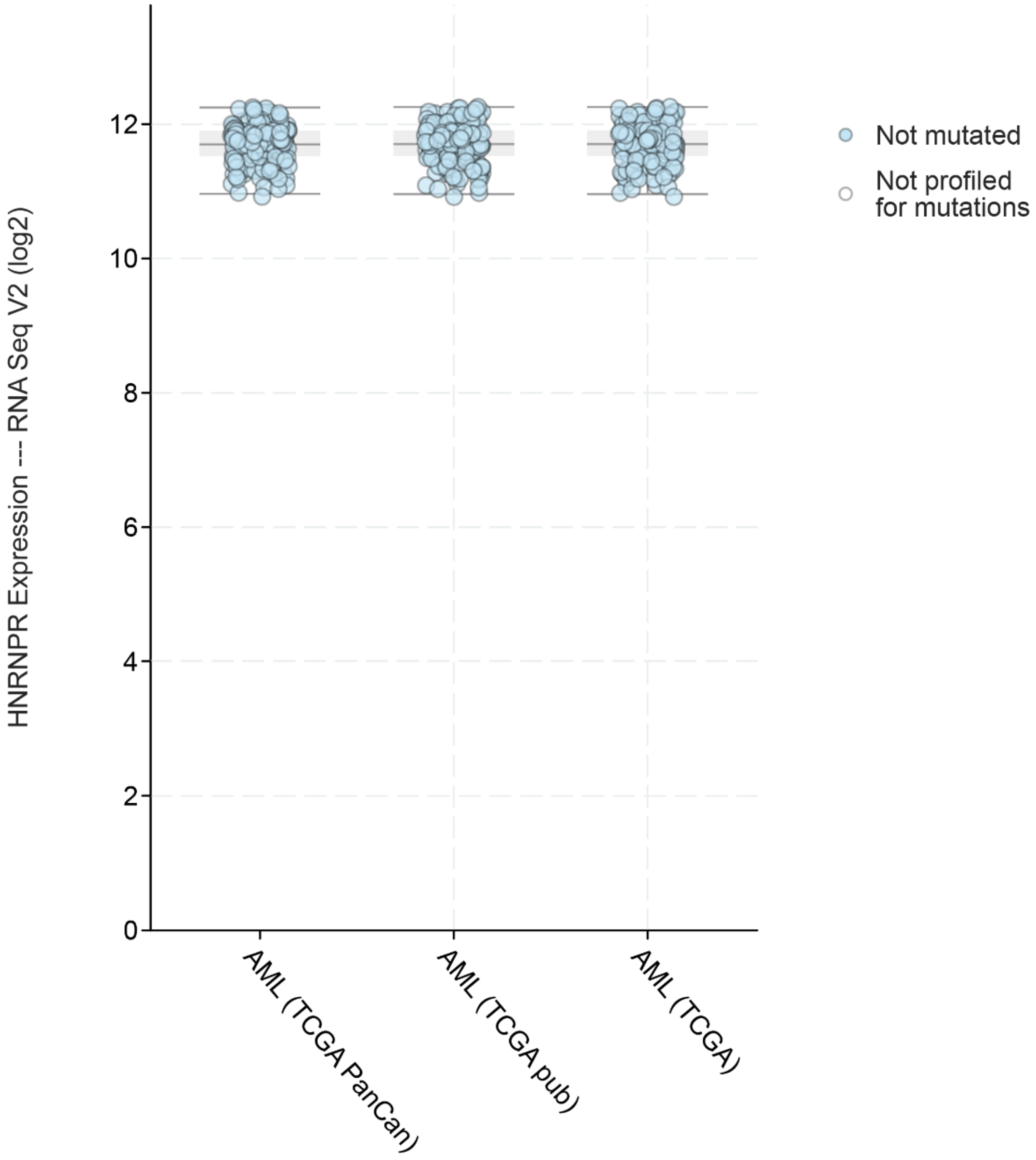

(b)

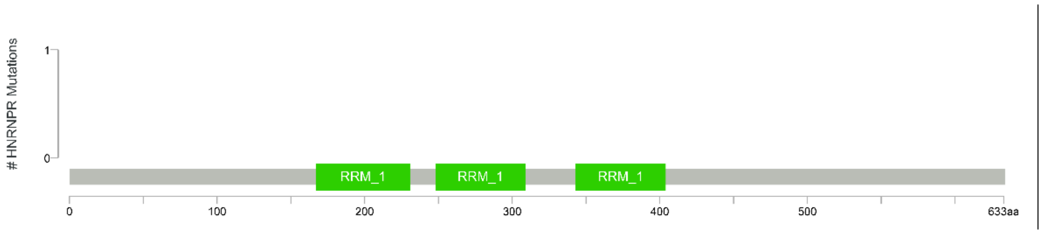

Figure S16. The alterations of *HNRNPR* gene in cBioportal Web resource online. (a) Relative expression level as a function of relative copy number of *HNRNPR* gene was plotted in different database. (b) The distribution of *HNRNPR* mutations in leukemia across protein domains.

(a)

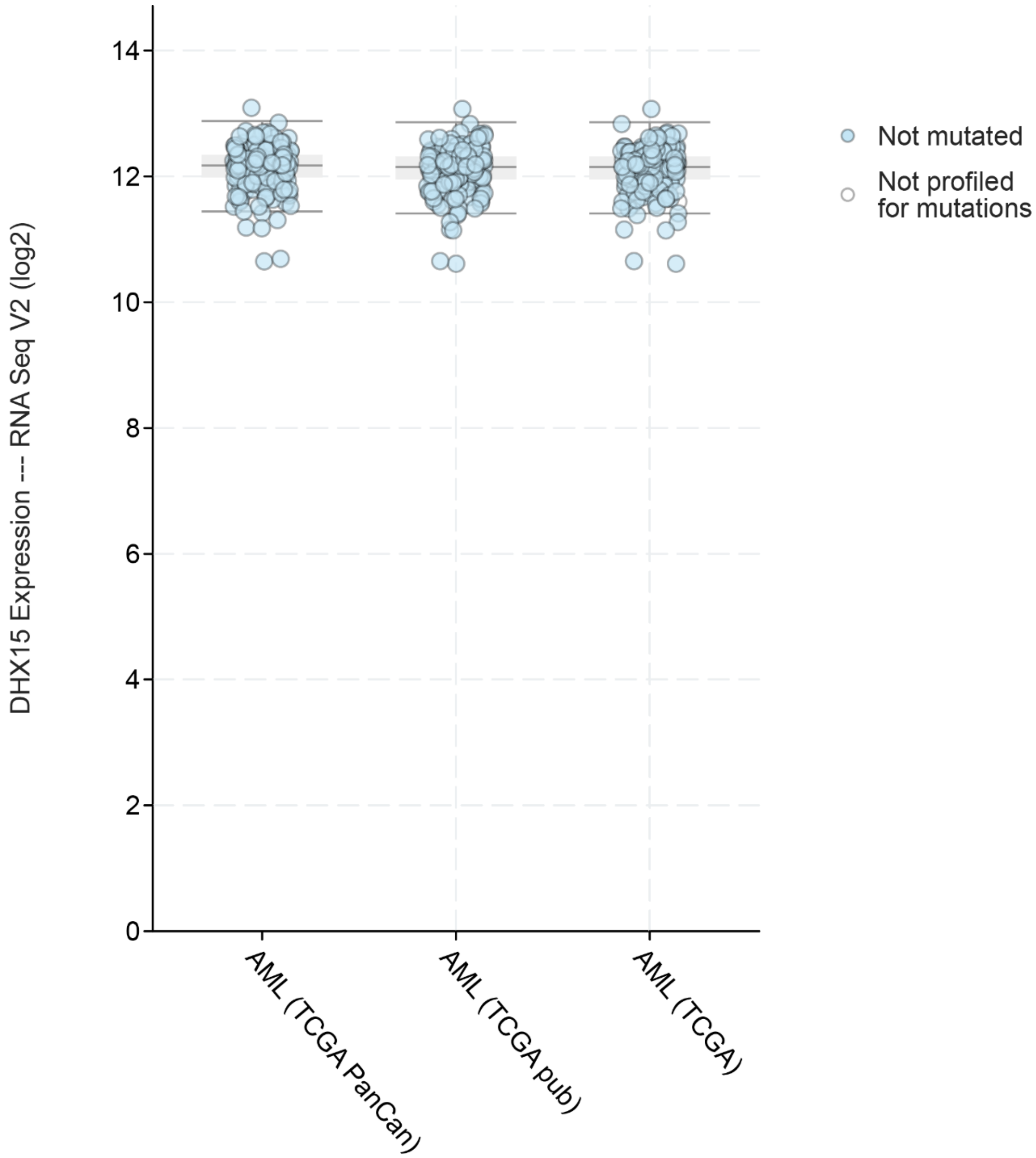

(b)

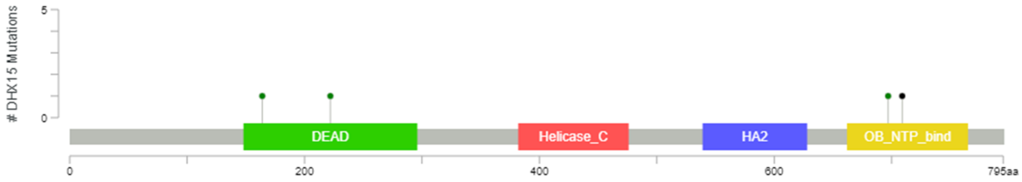

Figure S17. The alterations of *DHX15* gene in cBioportal Web resource online. (a) Relative expression level as a function of relative copy number of *DHX15* gene was plotted in different database. (b) The distribution of *DHX15* mutations in leukemia across protein domains.

(a)

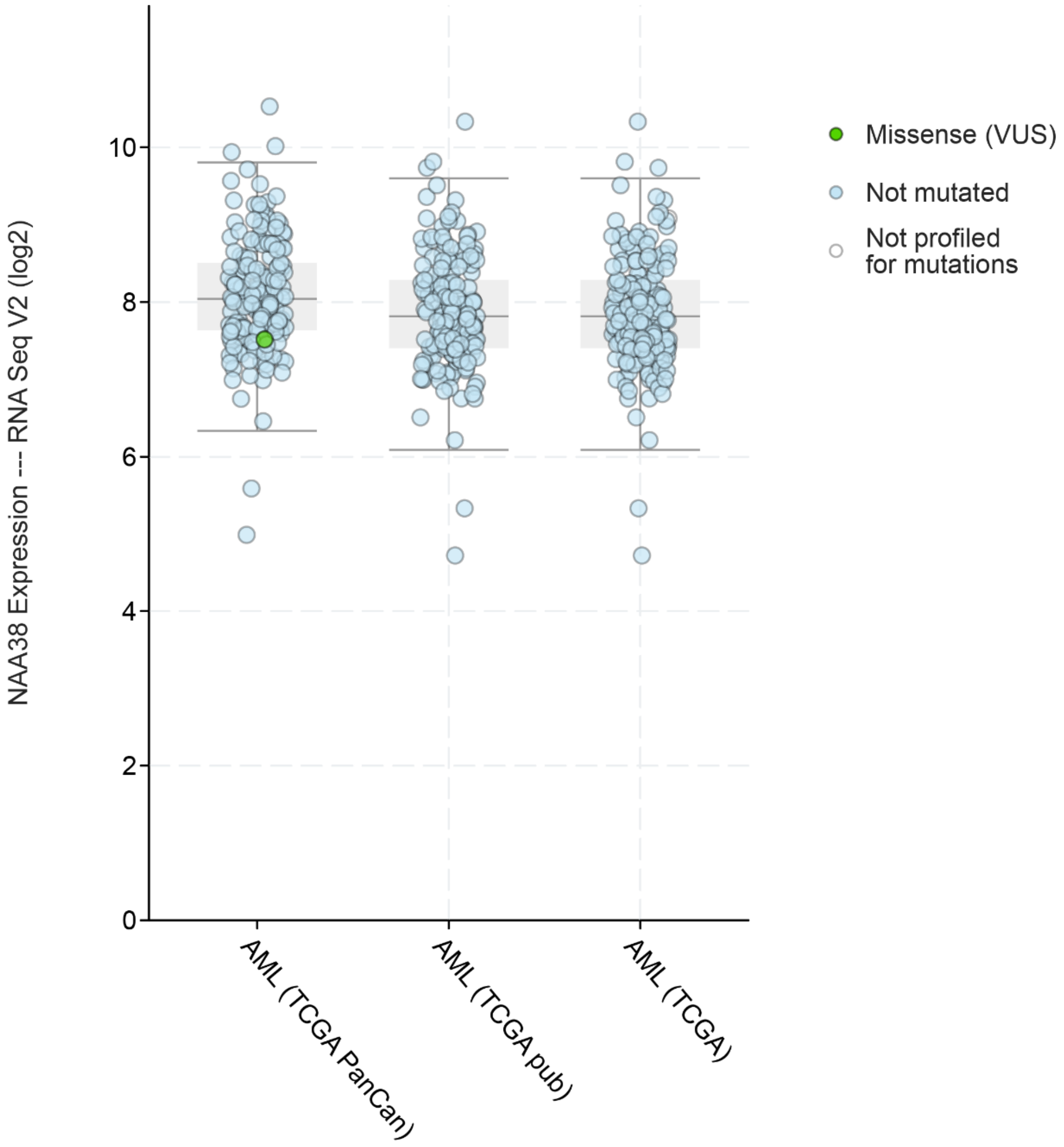

(b)

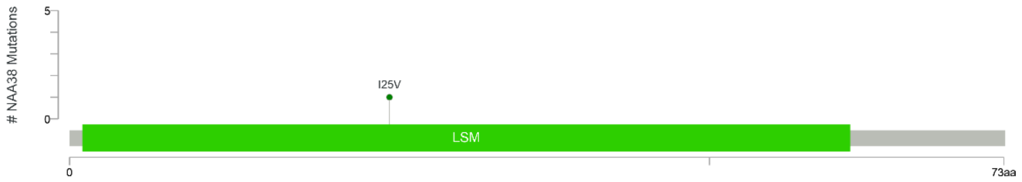

Figure S18. The alterations of *NAA38* gene in cBioportal Web resource online. (a) Relative expression level as a function of relative copy number of *NAA38* gene was plotted in different database. (b) The distribution of *NAA38* mutations in leukemia across protein domains.

(a)

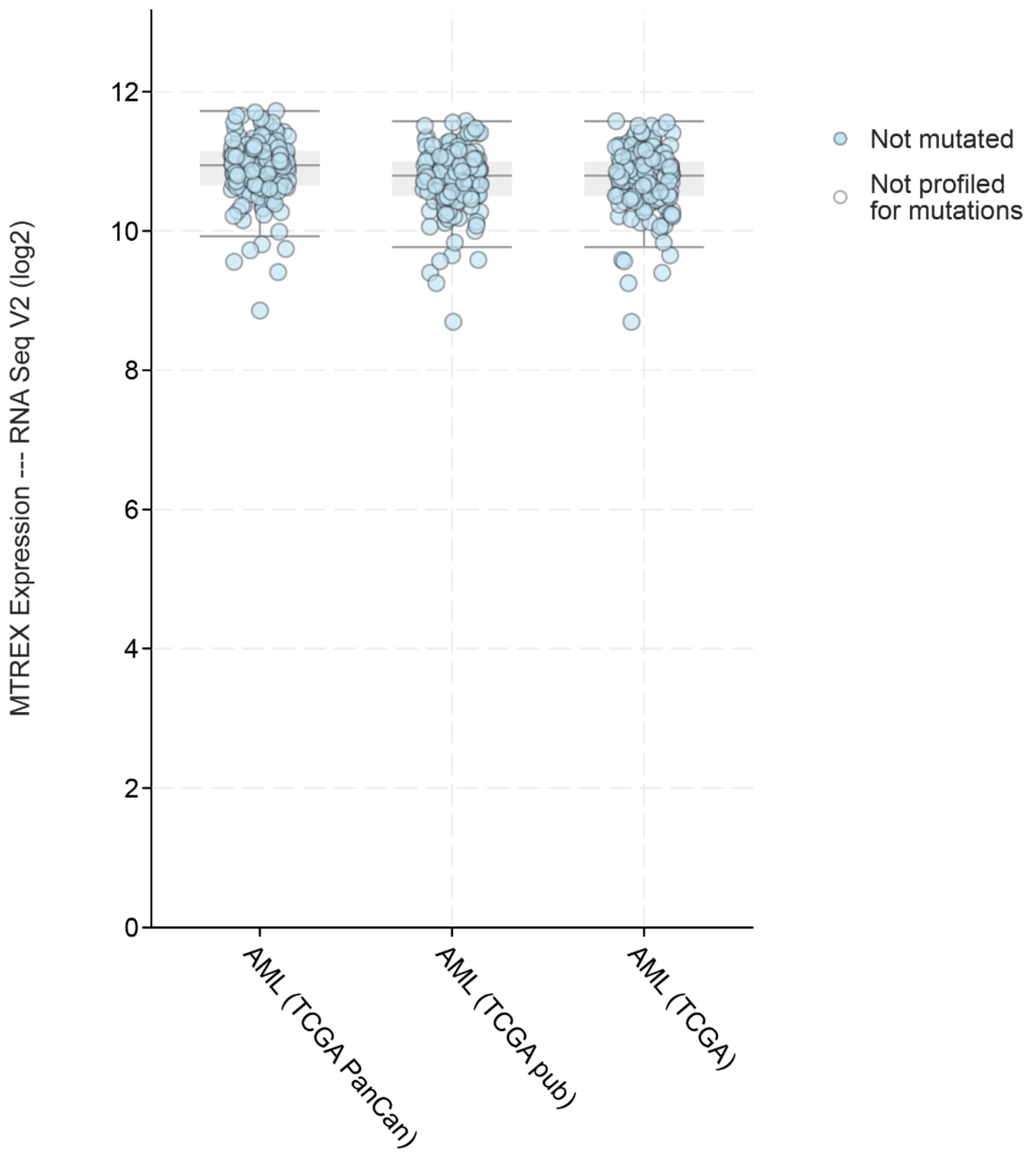

(b)

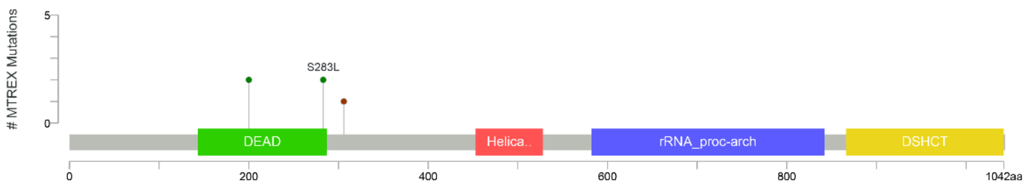

Figure S19. The alterations of *SKIV2L2* gene in cBioportal Web resource online. (a) Relative expression level as a function of relative copy number of *SKIV2L2 (MTREX)* gene was plotted in different database. (b) The distribution of *SKIV2L2 (MTREX)* mutations in leukemia across protein domains.

(a)

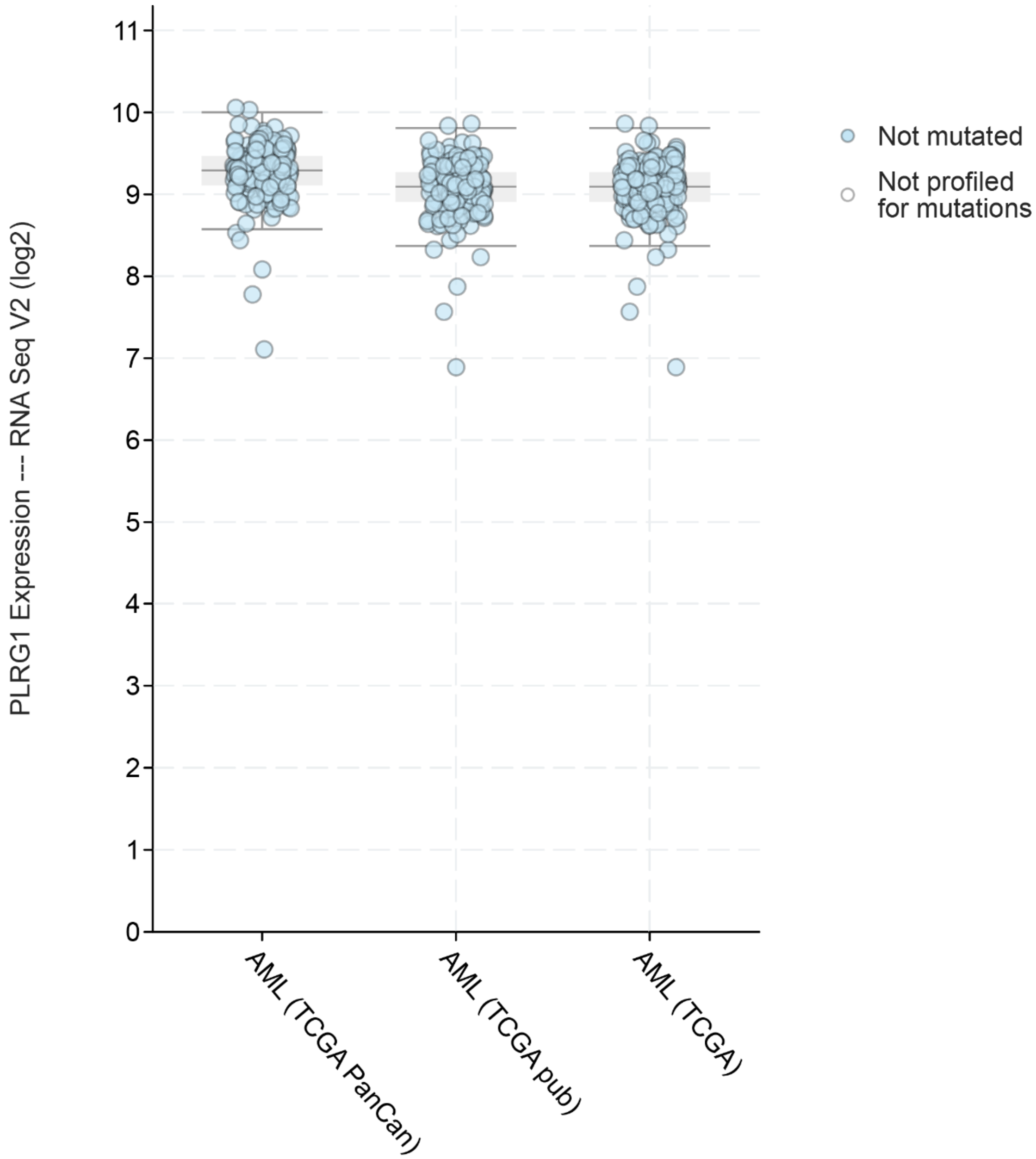

(b)

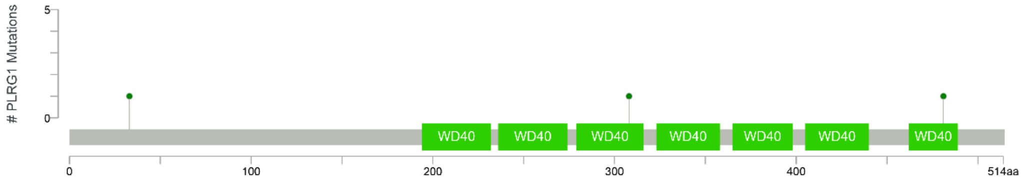

Figure S20. The alterations of *PLRG1* gene in cBioportal Web resource online. (a) Relative expression level as a function of relative copy number of *PLRG1* gene was plotted in different database. (b) The distribution of *PLRG1* mutations in leukemia across protein domains.

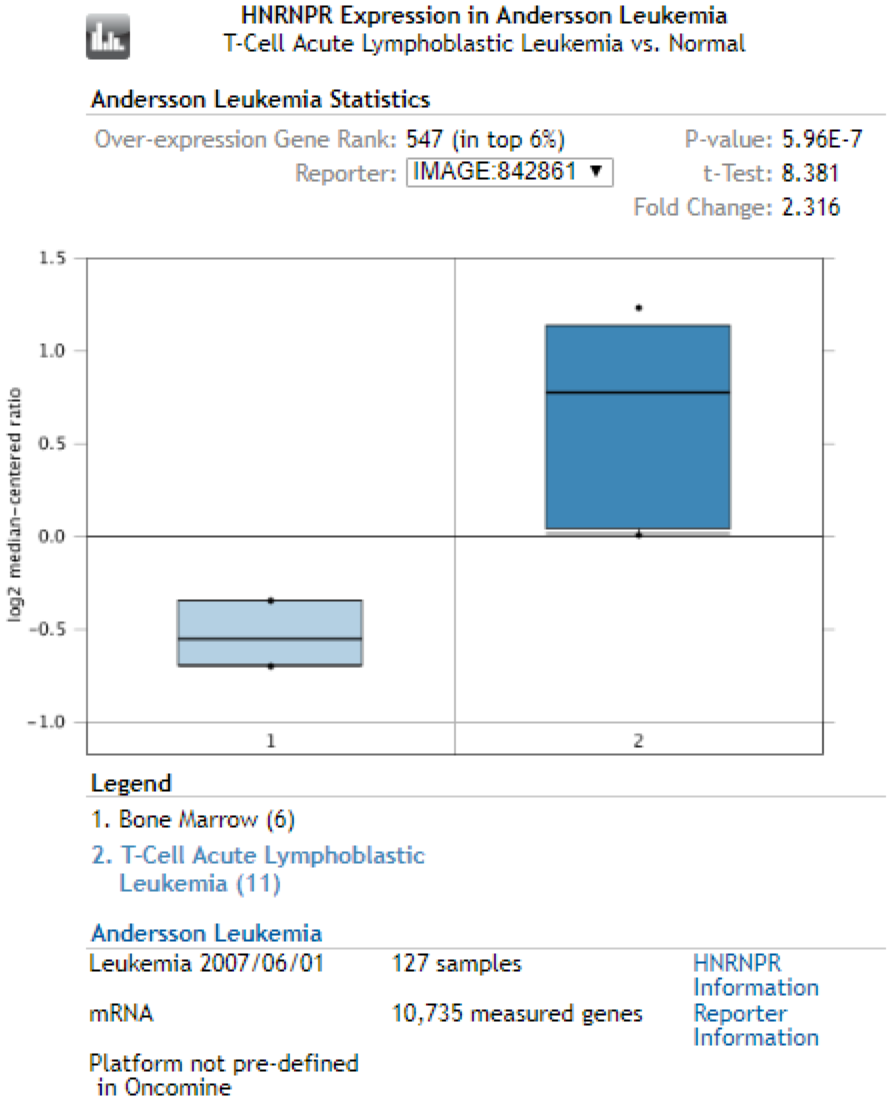

Figure S21. Comparison of the expression of *HNRNPR* in the Valk Leukemia database using Oncomine.

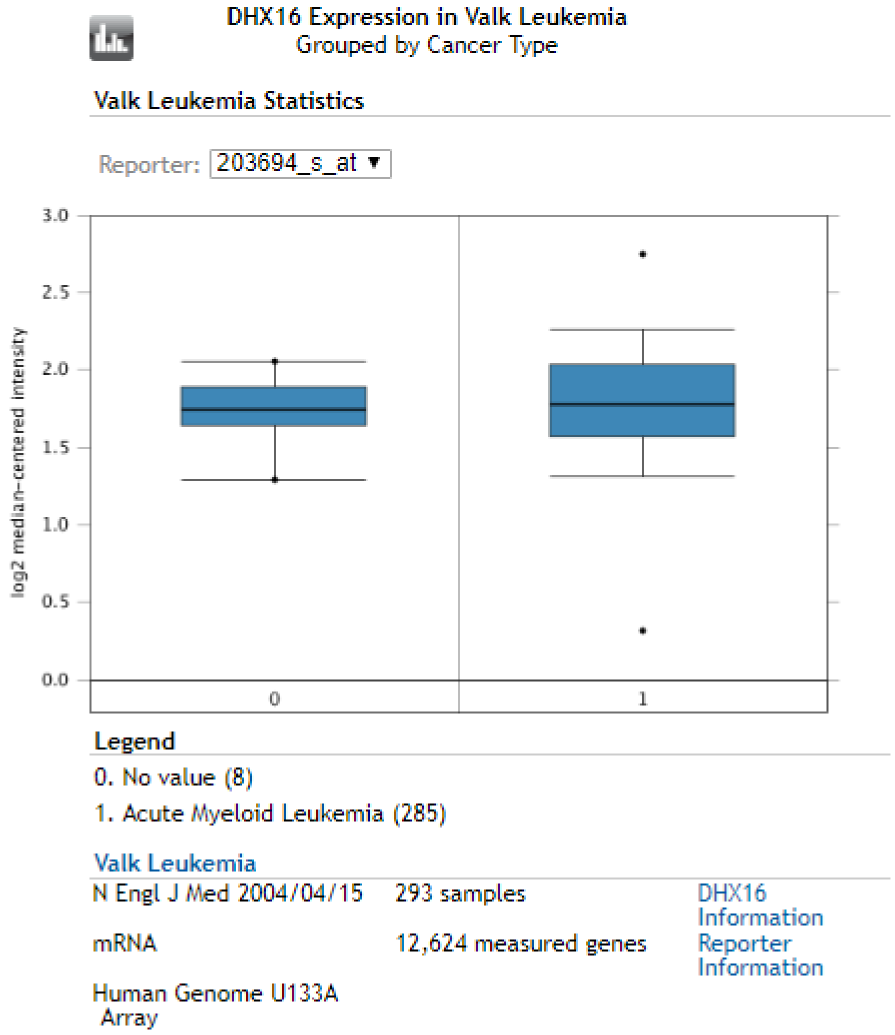

Figure S22. Comparison of the expression of *DHX16* in the Valk Leukemia database using Oncomine.

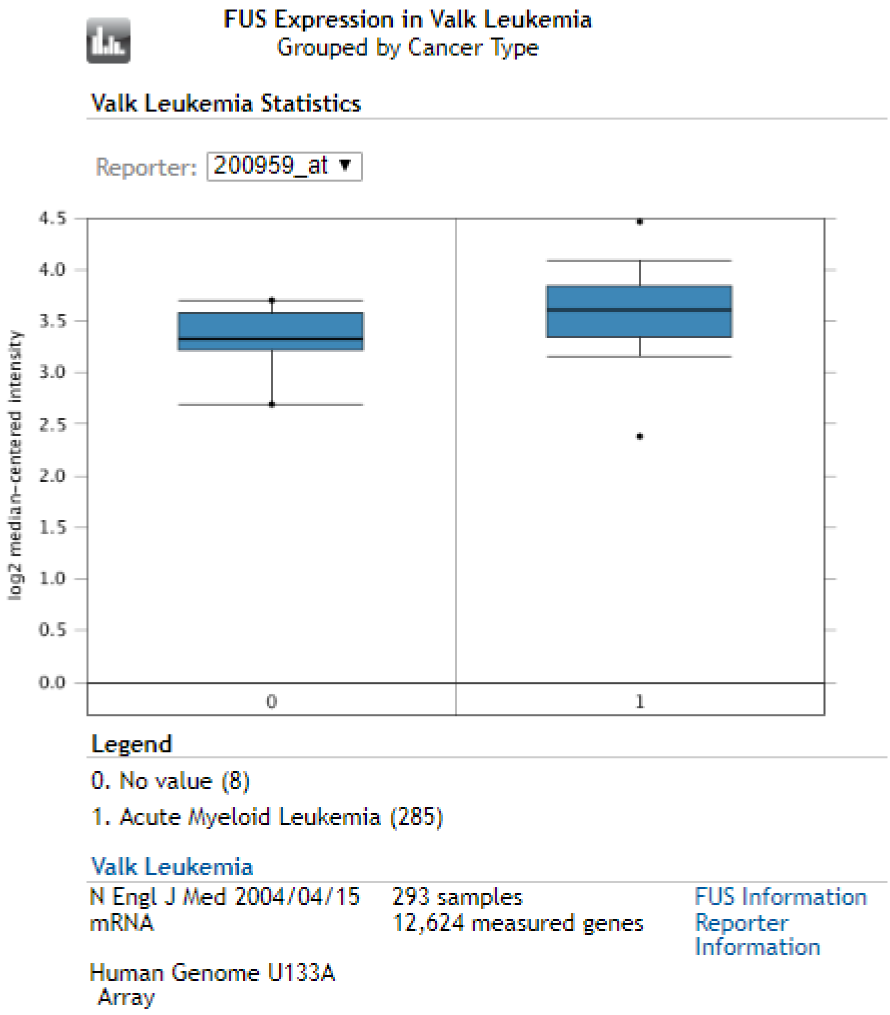

Figure S23. Comparison of the expression of *FUS* in the Valk Leukemia database using Oncomine.

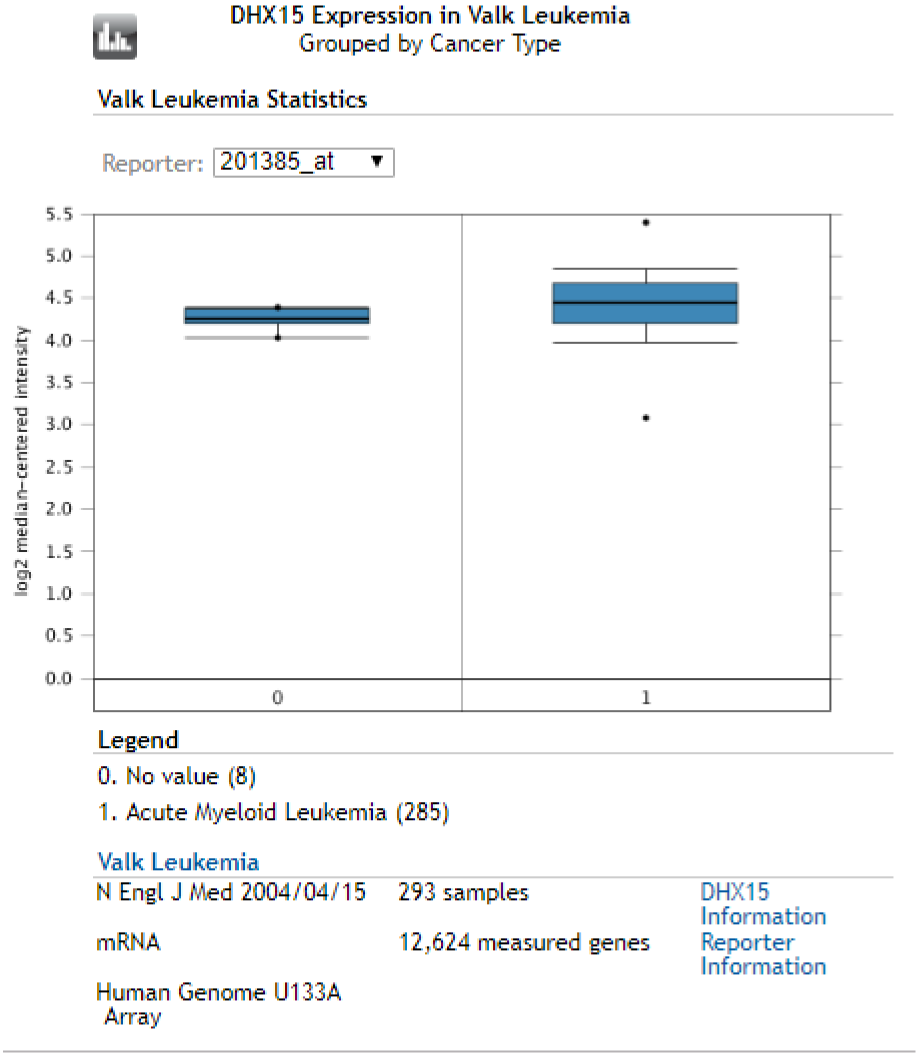

Figure S24. Comparison of the expression of *DHX15* in the Valk Leukemia database using Oncomine.

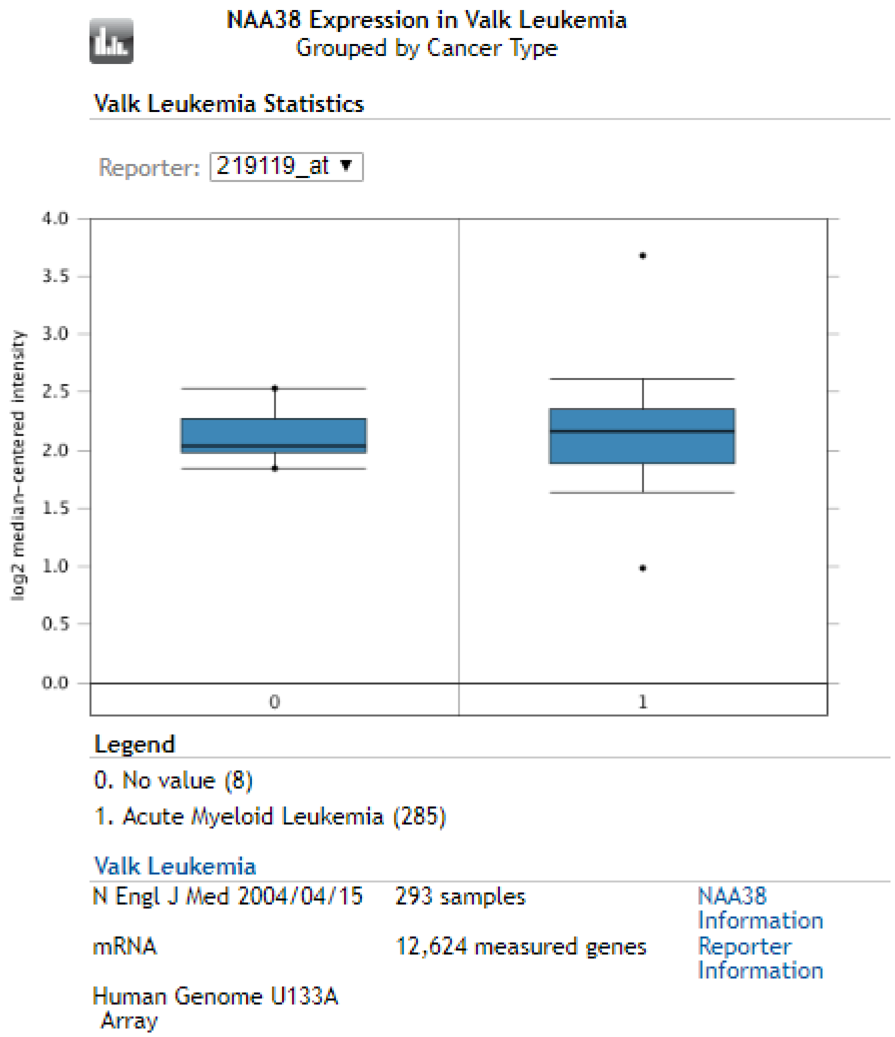

Figure S25. Comparison of the expression of *NAA38* in the Valk Leukemia database using Oncomine.

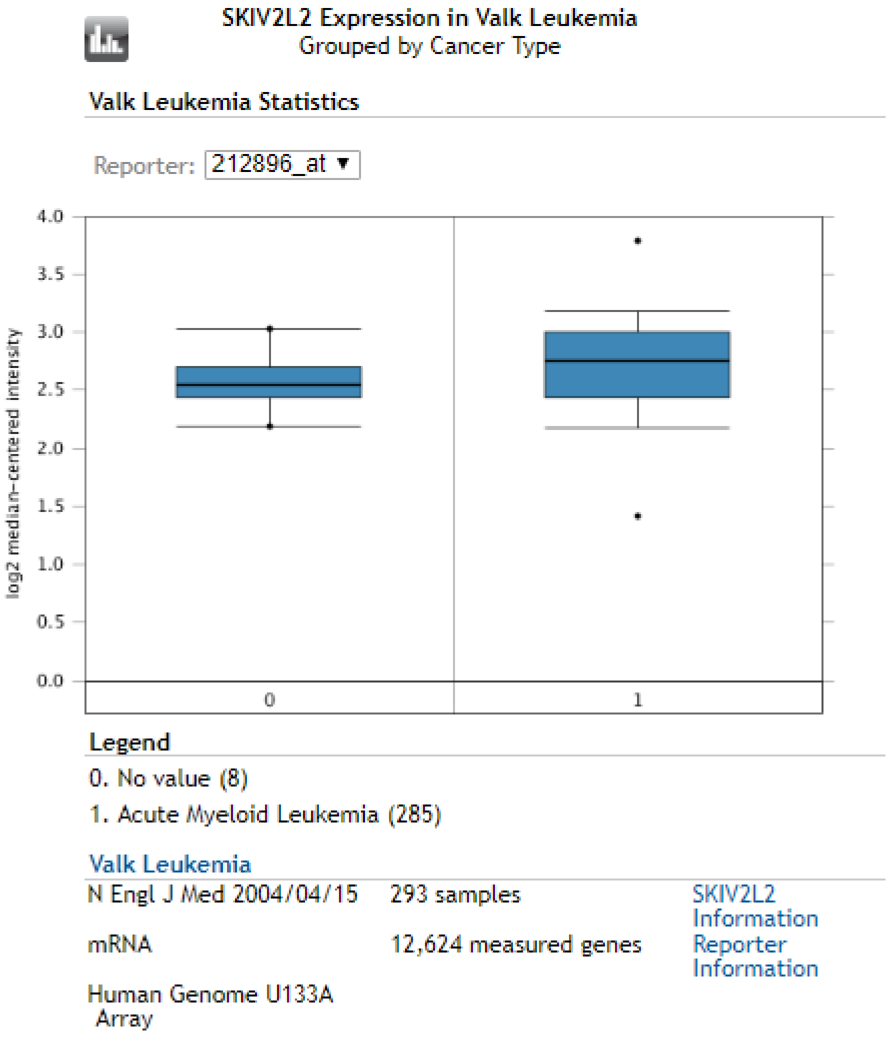

Figure S26. Comparison of the expression of *SKIV212* in the Valk Leukemia database using Oncomine.

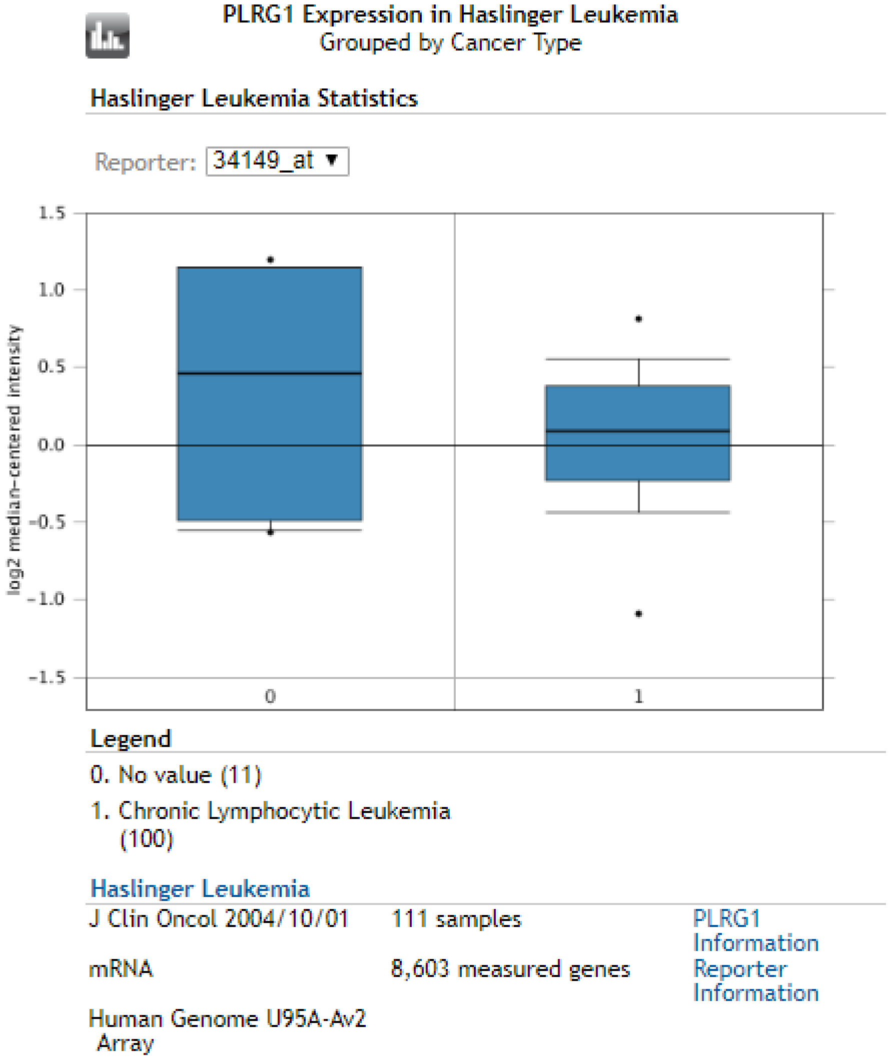

Figure S27. Comparison of the expression of *PLRG1* in the Haslinger Leukemia database using Oncomine.

(a)

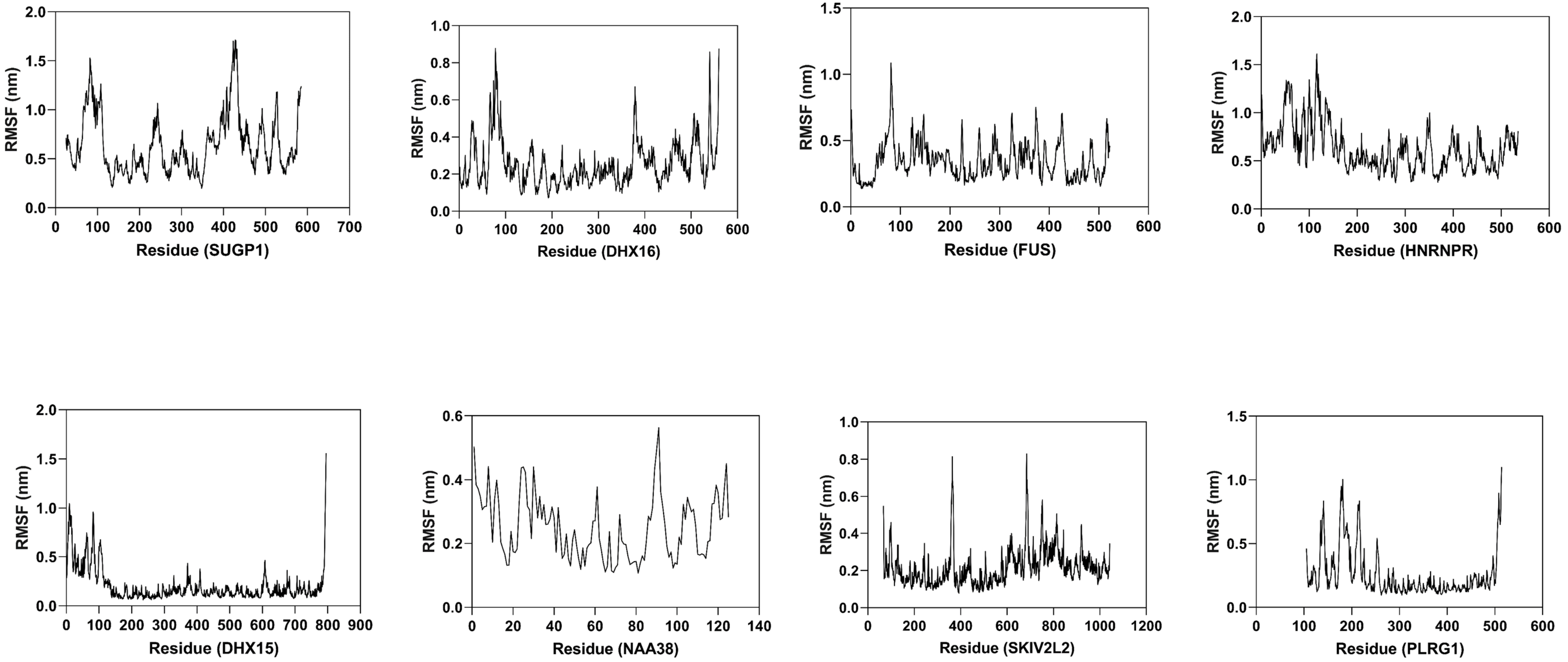

RMSF (nm)
Residue (SUGP1)
RMSF (nm)
Residue (DHX16)
RMSF (nm)
Residue (FUS)
RMSF (nm)
Residue (HNRNPR)
RMSF (nm)
Residue (DHX15)
RMSF (nm)
Residue (NAA38)
RMSF (nm)
Residue (SKIV2L2)
RMSF (nm)
Residue (PLRG1)

(b)

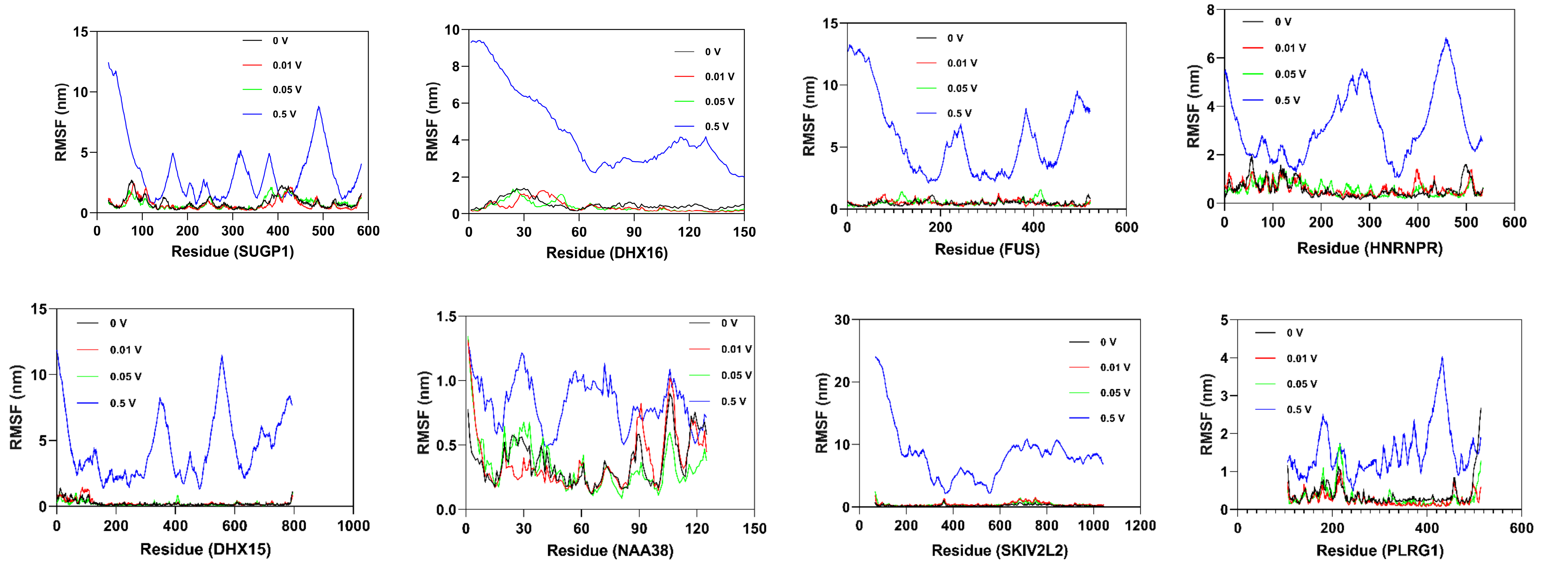

Figure S28. RMSF comparison plots of hub proteins during molecular dynamics simulation (at least > 100 ns). (a) The RMSF of each hub proteins under MD of “protein in water”. (b) The RMSF of selected hub proteins under MD of “protein under nsPEFs”. For each protein, 0v (black), 0.01v (red), 0.05v (green), and 0.5v (blue) are demonstrated on one map.

(a)

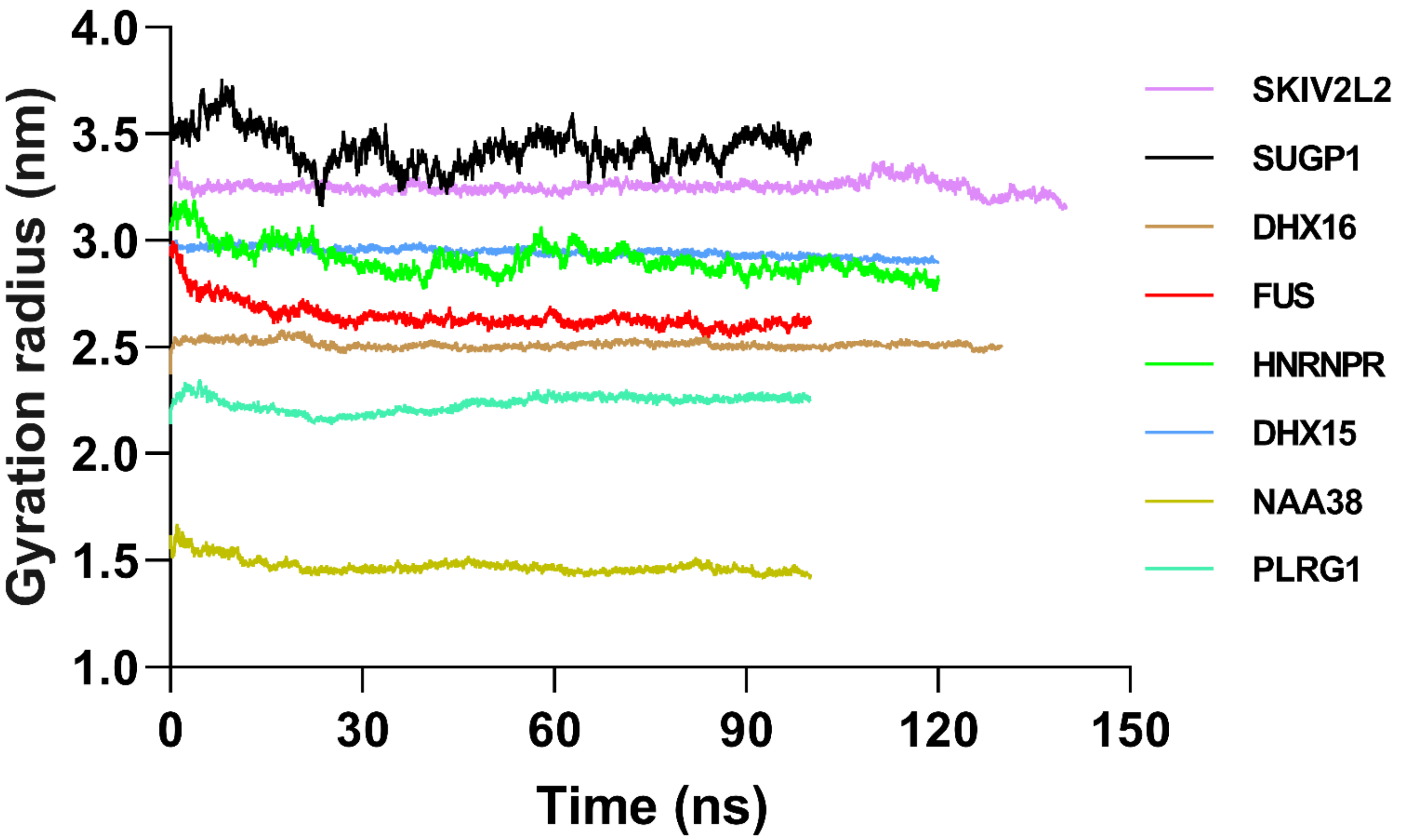

Gyration radius (nm)
4.0
3.5
3.0
2.5
2.0
1.5
1.0
0
30
60
90
120
150
Time (ns)
SKIV2L2
SUGP1
DHX16
FUS
HNRNPR
DHX15
NAA38
PLRG1

(b)

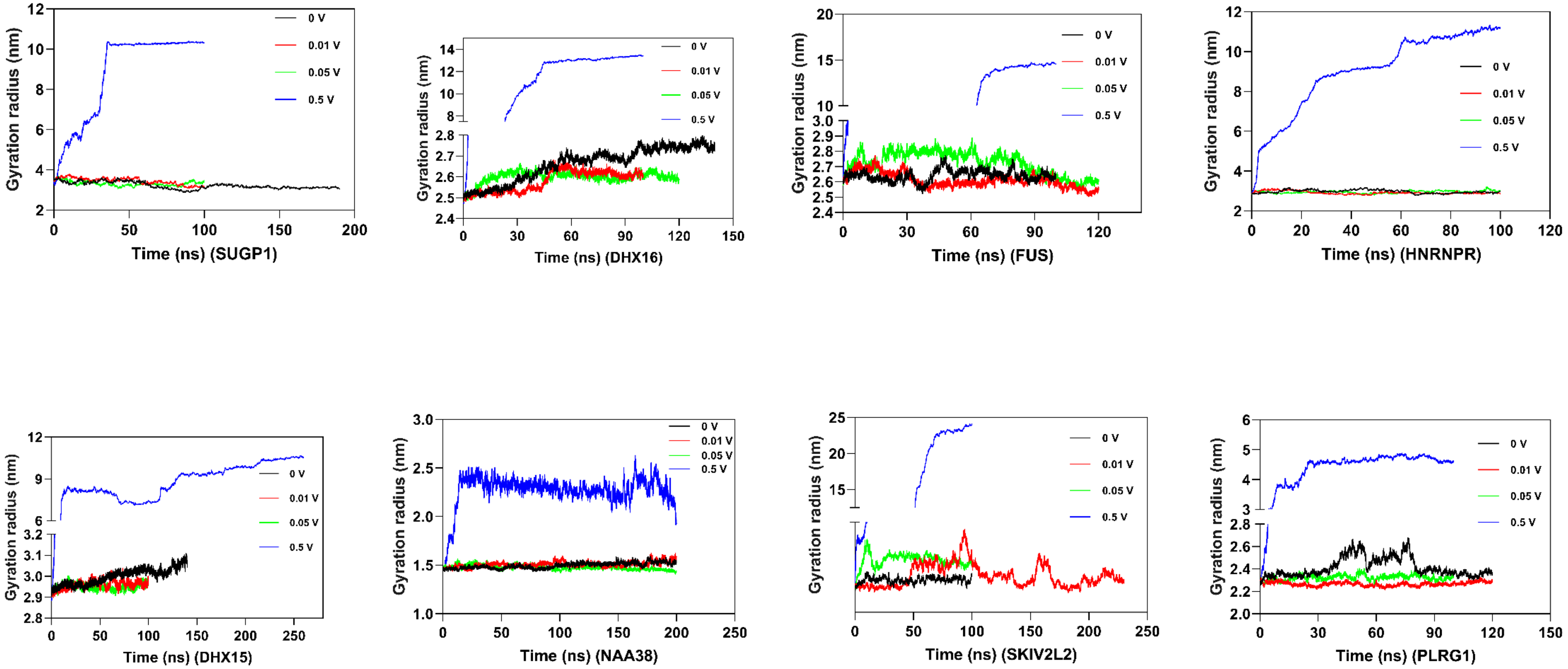

Figure S29. Gyration radius comparison plots of hub protein during molecular dynamics simulation (at least > 100 ns). (a) The gyration radius of each hub proteins under MD of “protein in water”. (b) The gyration radius of selected hub proteins under MD of “protein under nsPEFs”. For each protein, 0v (black), 0.01v (red), 0.05v (green), and 0.5v (blue) are demonstrated on one map.

a

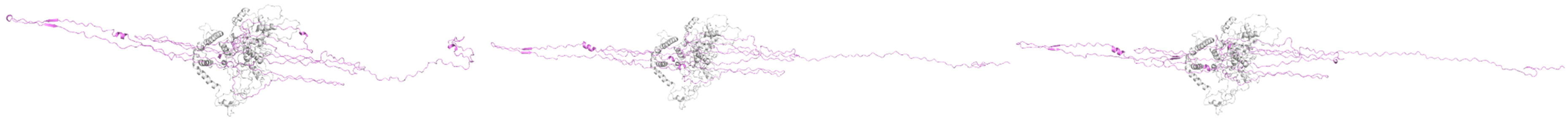

SUGP1_alignment_0.01_0v SUGP1_alignment_0.05_0v SUGP1_alignment_0.5_0v

b

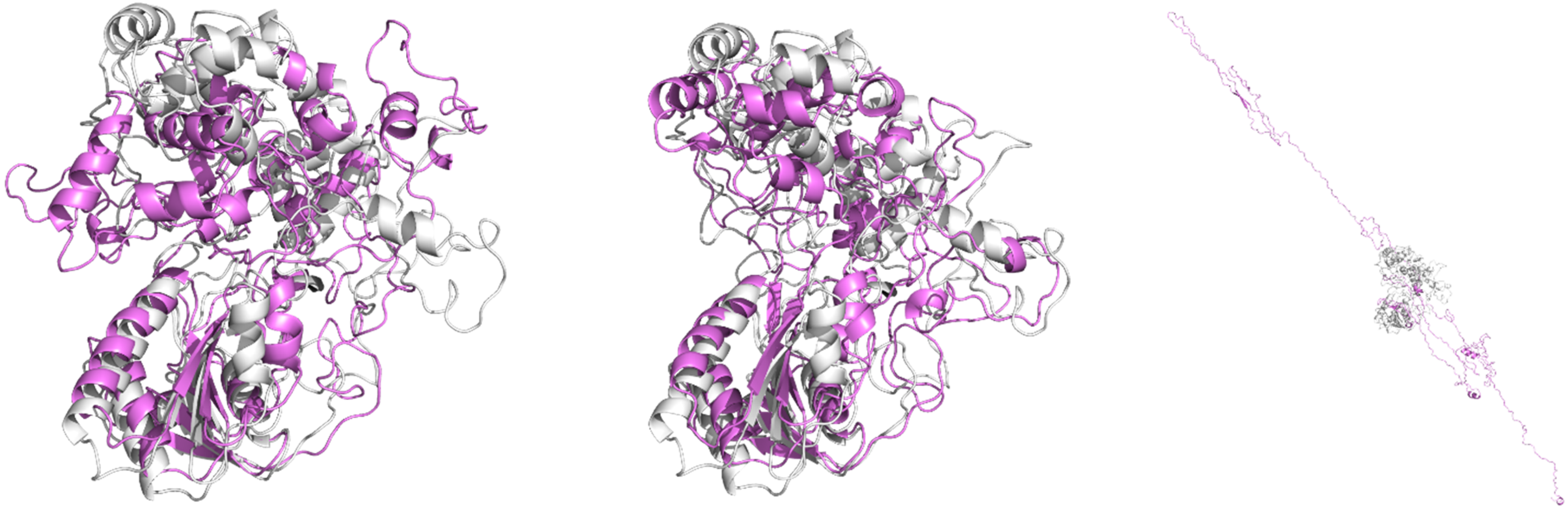

DHX16_alignment_0.01_0v DHX16_alignment_0.05_0v DHX16_alignment_0.5_0v

c

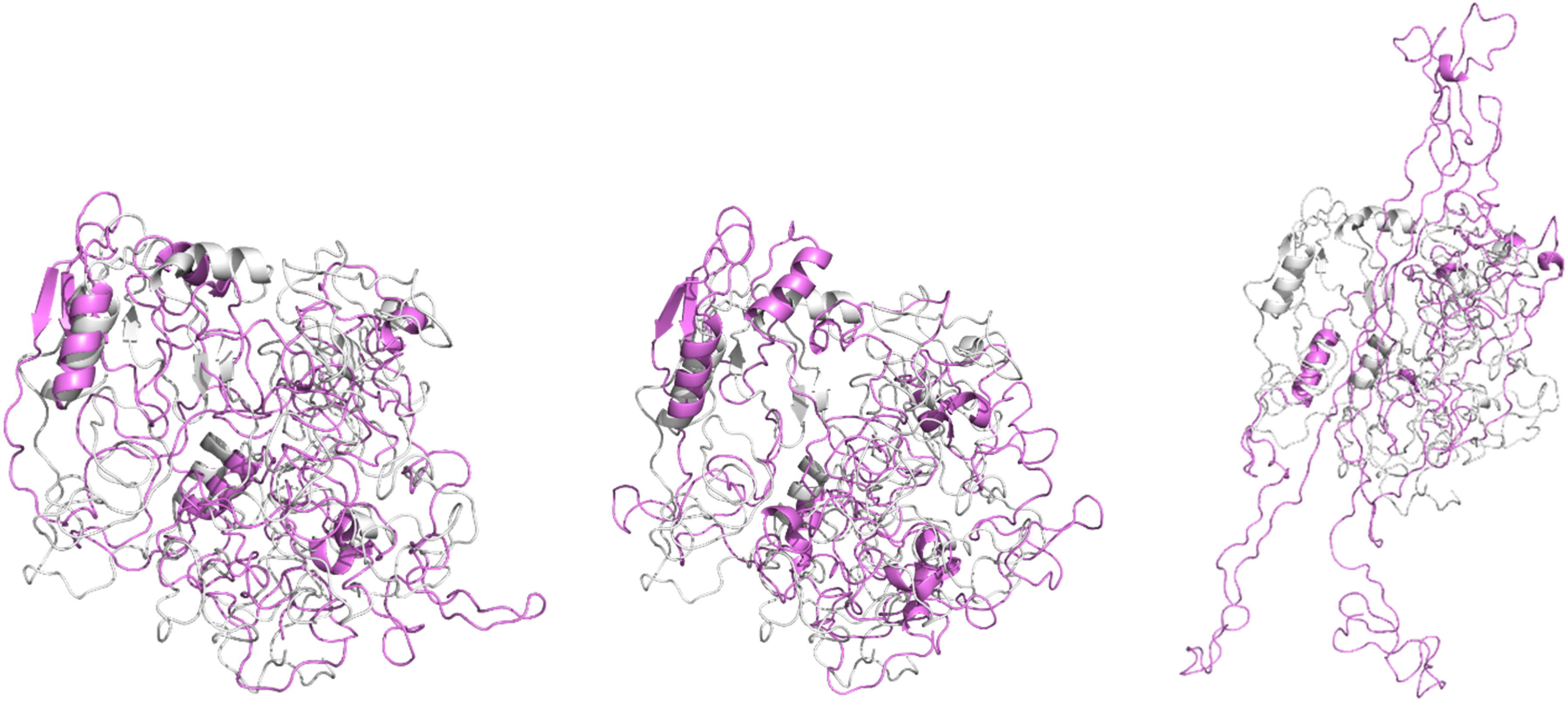

FUS_alignment_0.01_0v FUS_alignment_0.05_0v FUS_alignment_0.5_0v

d

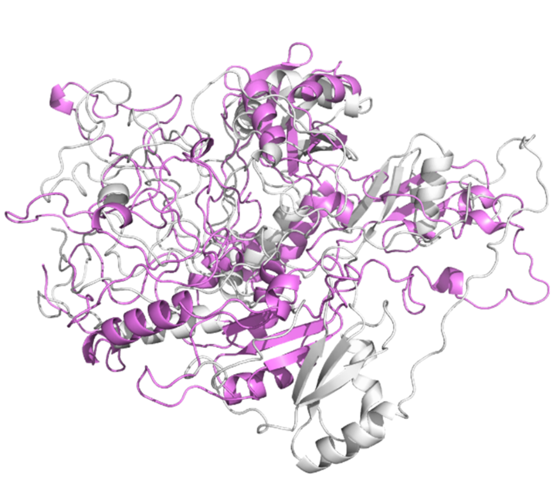

HNRNPR_alignment_0.01v_0v

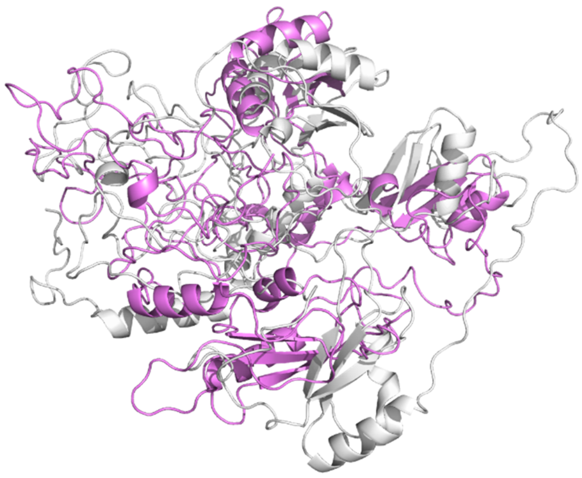

HNRNPR_alignment_0.05v_0v

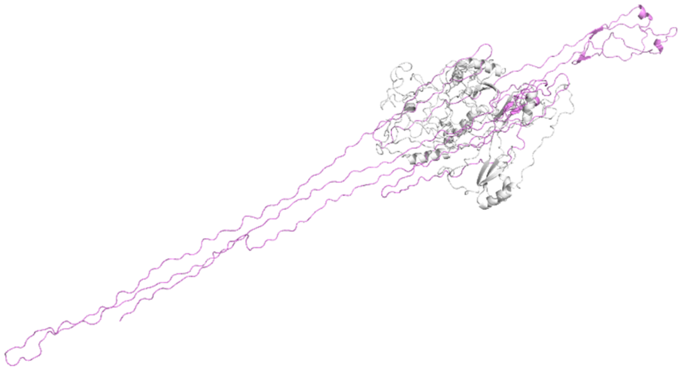

HNRNPR_alignment_0.5v_0v

e

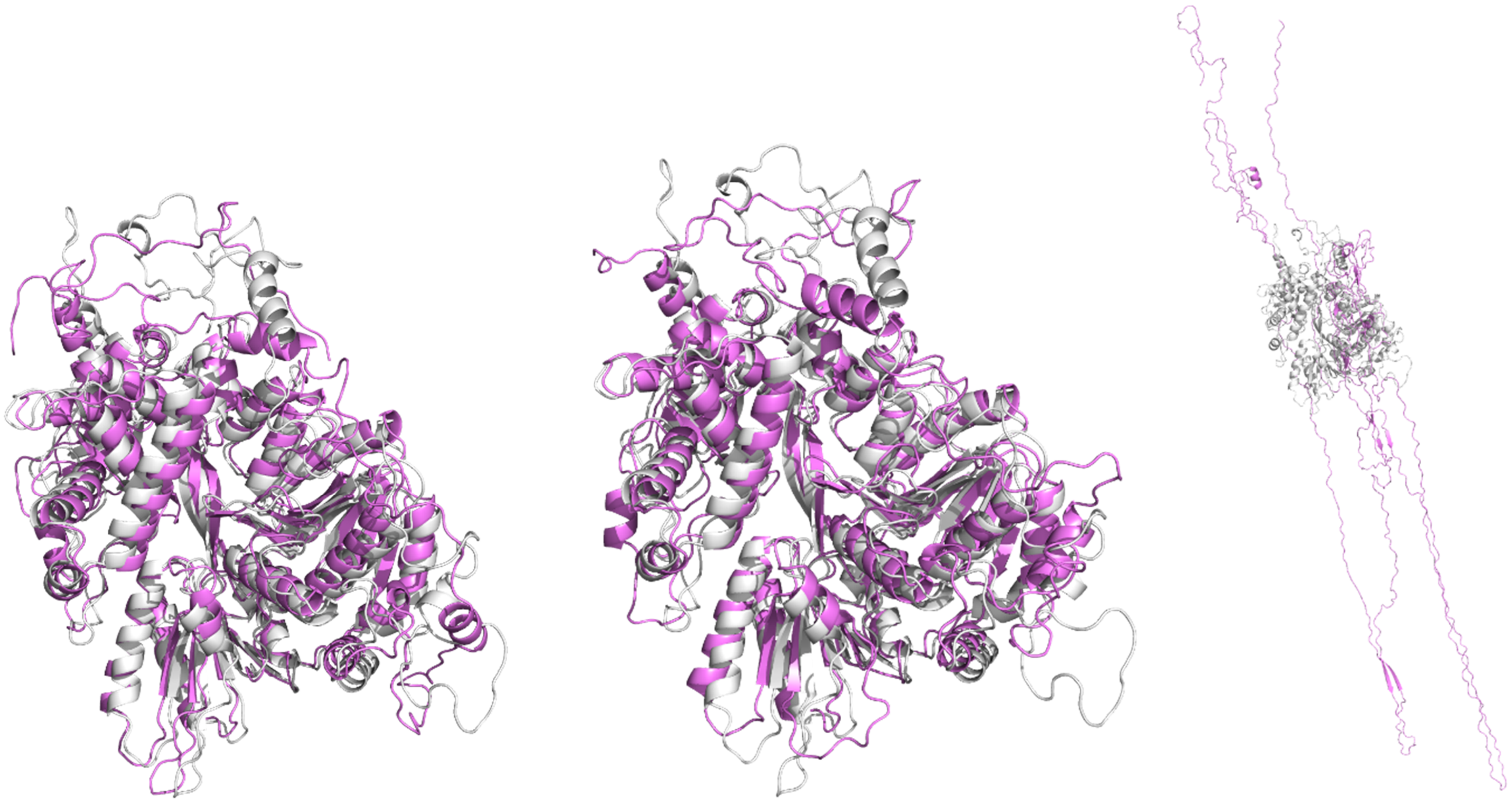

DHX15_alignment_0.01_0v

DHX15_alignment_0.05_0v

DHX15_alignment_0.5_0v

f

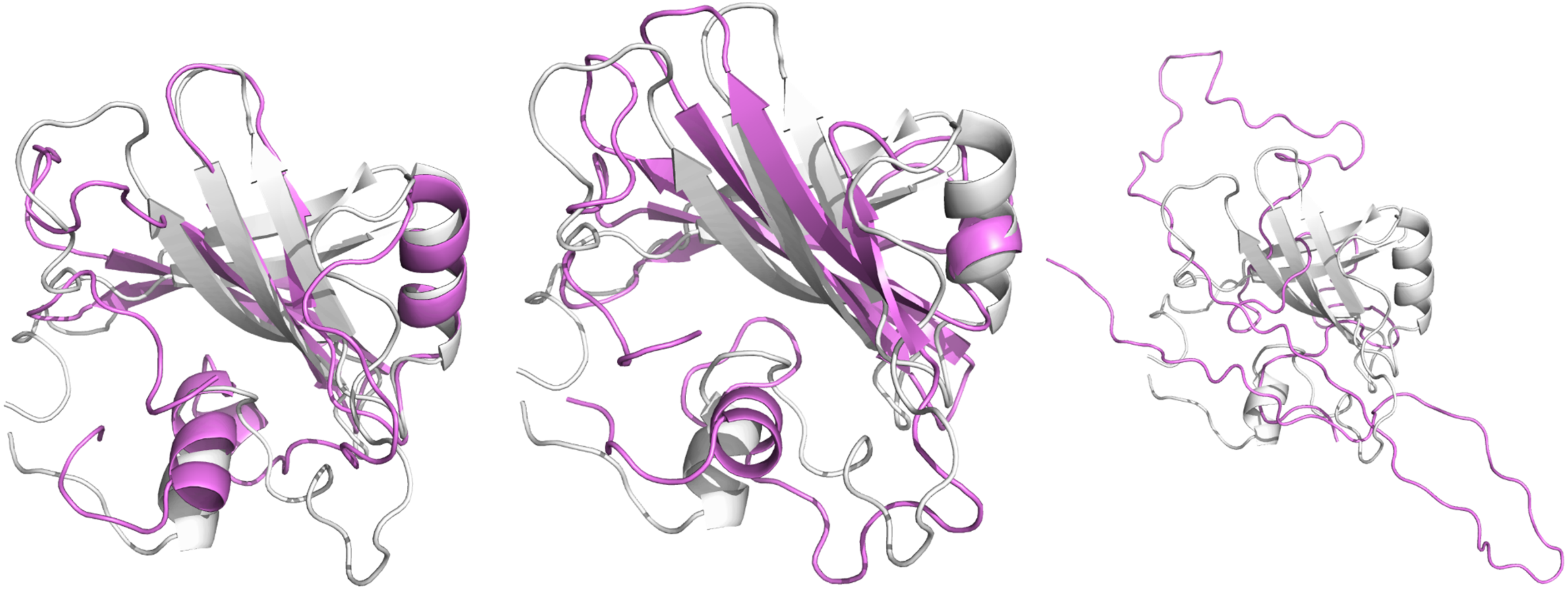

NAA38_alignment_0.01v_0v

NAA38_alignment_0.05v_0v

NAA38_alignment_0.5v_0v

g

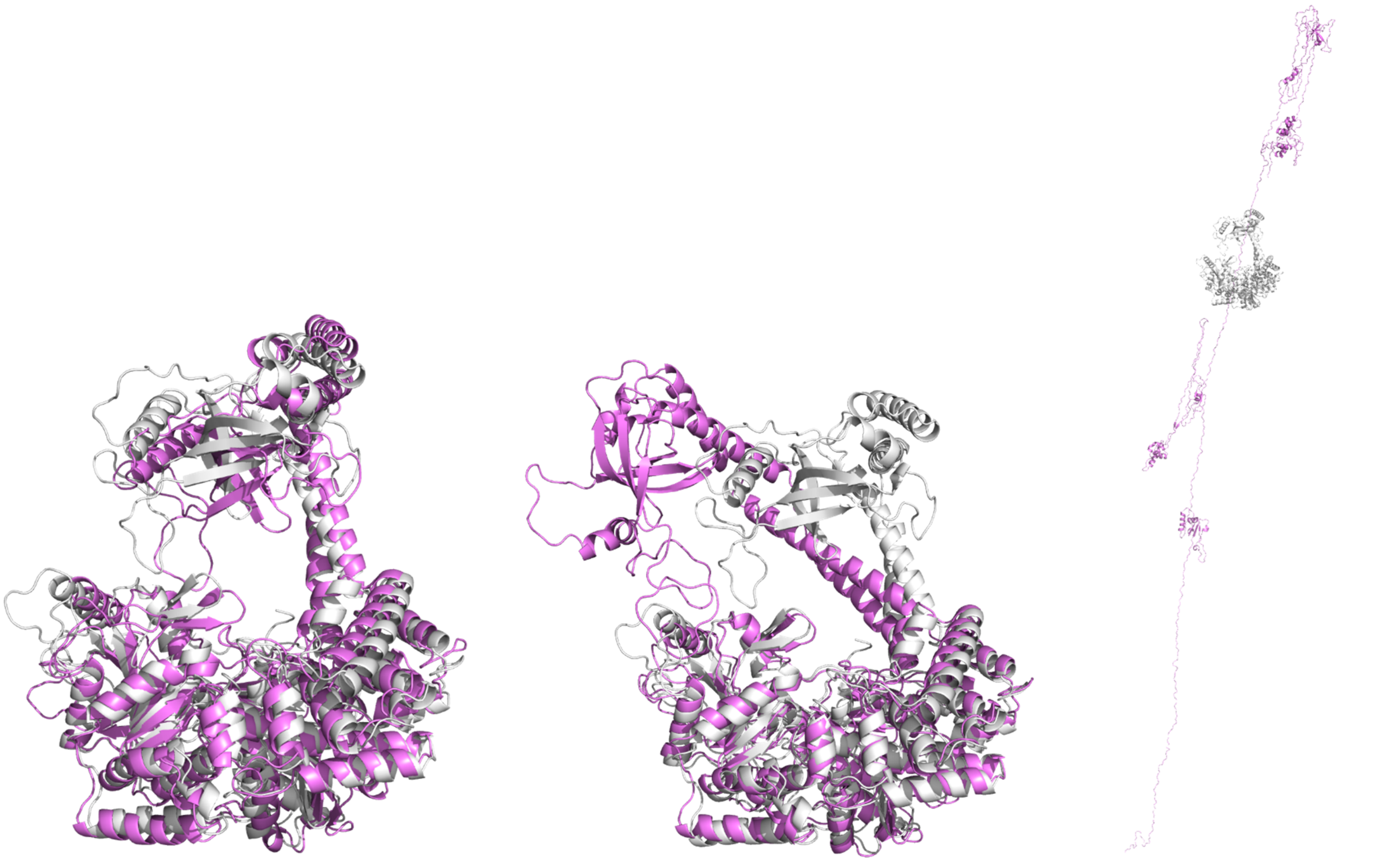

SKIV2L2_alignment_0.01v_0v

SKIV2L2_alignment_0.05v_0v

SKIV2L2_alignment_0.5v_0v

h

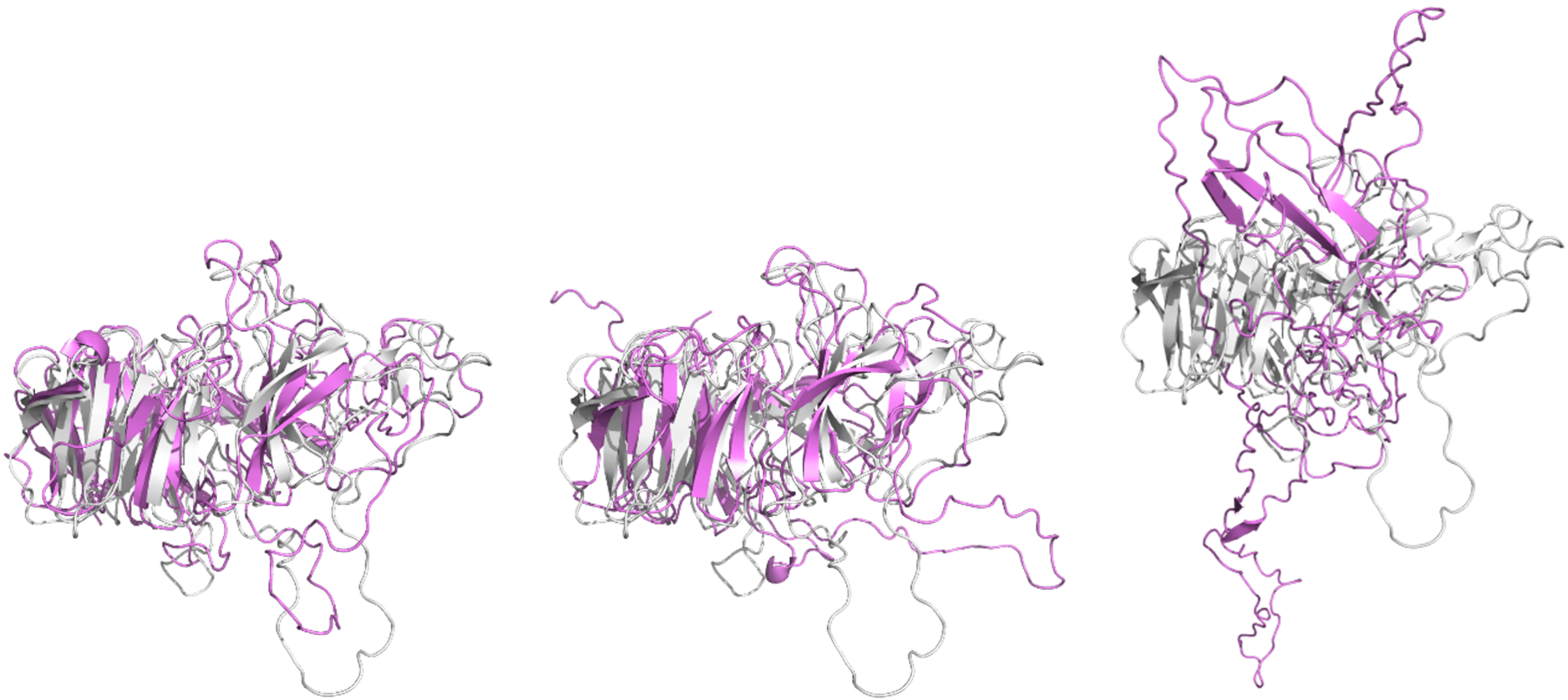

PLRG1_alignment_0.01_0v PLRG1_alignment_0.05_0v PLRG1_alignment_0.5_0v

Figure S30. Superposition of the MD-optimized (0V) protein structure (gray) and the MD-optimized (0.01, 0.05 and 0.5 mV/mm) protein structure (violet).
