# Supplementary material for: Cellular Processes Involved in Jurkat Cells Exposed to Nanosecond Pulsed Electric Field
Source: Int J Mol Sci. 2019 Nov 21;20(23):5847. doi: 10.3390/ijms20235847 (PMC6929111; doi:10.3390/ijms20235847)
Supplement: Supplementary file 1 [file ijms-20-05847-s001.zip › Supplementary file 2 -tables_proof1.pdf]

Supplementary Information

Cellular processes involved in Jurkat cells exposed to nanosecond pulsed electric field

Huijuan Li [1], Shibin Liu [1,*], Xue Yang [1], Yongqian Du [1], Jiezhang Luo [1], Jie Tan [1], and Yulong Sun [2,*]

1 School of Electronics and Information, Northwestern Polytechnical University, Xi'an, 710072, China; lihuijuan@mail.nwpu.edu.cn (H.J.L); liushibin@nwpu.edu.cn (S.B.L); yangx@mail.nwpu.edu.cn (X.Y); duyongqian@nwpu.edu.cn (Y.Q.D); 18829237964@163.com (J.Z.L); jietan@mail.nwpu.edu.cn (J.T)

2 Key Laboratory for Space Biosciences & Biotechnology, School of Life Sciences, Northwestern Polytechnical University, Xi'an, 710072, China; yulongsun@nwpu.edu.cn (Y.L.S)

* Correspondence: liushibin@nwpu.edu.cn (S.B.L); yulongsun@nwpu.edu.cn (Y.L.S);

Tel.: +86 29 88491653 (S.B.L); +86 29 88460332 (Y.L.S)

Address: School of Life Sciences, Northwestern Polytechnical University, 127 Youyi Xilu, Xi' an Shaan Xi Province, 710072, PR China

Tel.: +86 29 88460332, E-mail: yulongsun@nwpu.edu.cn

Table of contents

1. Tables

.

Table S1 RMSD value average analysis

Proteins	Model[a]	0 v[b]	0.01 v[b]	0.05 v[b]	0.5 v[b]
SUGP1	1.761	N.D	N.D	N.D	N.D
DHX16	0.5759	0.6216	0.5533	0.5988	9.31
FUS	1.029	0.9463	0.9185	0.853	7.352
HNRNPR	1.517	N.D	N.D	N.D	N.D
DHX15	0.3965	0.4047	0.5067	0.3902	7.83
NAA38	0.6192	N.D	N.D	N.D	N.D
SKIV2L2	0.4343	N.D	N.D	N.D	N.D
PLRG1	0.6941	0.6674	0.4666	0.7016	3.635

Table S2 RMSF value average analysis

Proteins	Model[a]	0 v[b]	0.01 v[b]	0.05 v[b]	0.5 v[b]
SUGP1	0.6416	N.D	N.D	N.D	N.D
DHX16	0.2550	0.3628	0.2882	0.2725	3.601
FUS	0.3450	0.4874	0.4925	0.5140	5.702
HNRNPR	0.6293	N.D	N.D	N.D	N.D
DHX15	0.2026	0.2220	0.2545	0.1973	4.479
NAA38	0.2504	N.D	N.D	N.D	N.D
SKIV2L2	0.2165	N.D	N.D	N.D	N.D
PLRG1	0.2569	0.3955	0.2617	0.3194	1.531

Note:

a: compared with MD “protein in water”

b: compared with MD “protein in electric field”

N.D: none data

Table S3 R*g* value average analysis

Proteins	Model[a]	0 v[b]	0.01 v[b]	0.05 v[b]	0.5 v[b]
SUGP1	3.436	N.D	N.D	N.D	N.D
DHX16	2.512	2.662	2.579	2.598	10.46
FUS	2.646	2.641	2.611	2.722	8.217
HNRNPR	2.911	N.D	N.D	N.D	N.D
DHX15	2.945	2.999	2.960	2.949	8.709
NAA38	1.473	N.D	N.D	N.D	N.D
SKIV2L2	3.251	N.D	N.D	N.D	N.D
PLRG1	2.235	2.414	2.266	2.322	4.388

Note:

a: compared with MD “protein in water”

b: compared with MD “protein in electric field”

N.D: none data

Table S4 RMSD value of protein model and MD-optimized protein model

Proteins	Model[a]	0.01 v[b]	0.05 v[b]	0.5 v[b]
SUGP1	13.456			
DHX16	4.386	18.3	19.9	99.847
FUS	10.472	8.809	10.604	32.637
HNRNPR	14.815			
DHX15	1.978	2.201	1.797	73.054
NAA38	5.226			
SKIV2L2	1.775			
PLRG1	4.558	3.714	3.332	31.329
